# Enantioselective Amination of 4-Substituted Pyrazolones Catalyzed by Oxindole-Containing Thioureas and by a Recyclable Linear-Polymer-Supported Analogue in a Continuous Flow Process

**DOI:** 10.1021/acs.joc.3c02069

**Published:** 2023-12-14

**Authors:** Rodrigo Sánchez-Molpeceres, Laura Martín, Noelia Esteban, Jesús A. Miguel, Alicia Maestro, José M. Andrés

**Affiliations:** † SintACat, IU CINQUIMA y Departamento de Química Orgánica, Facultad de Ciencias, Universidad de Valladolid, Paseo Belén 7, Valladolid 47011, Spain; ‡ CLiNuMat, IU CINQUIMA y Departamento de Química Física y Química Inorgánica, Facultad de Ciencias, Universidad de Valladolid, Paseo Belén 7, Valladolid 47011, Spain

## Abstract

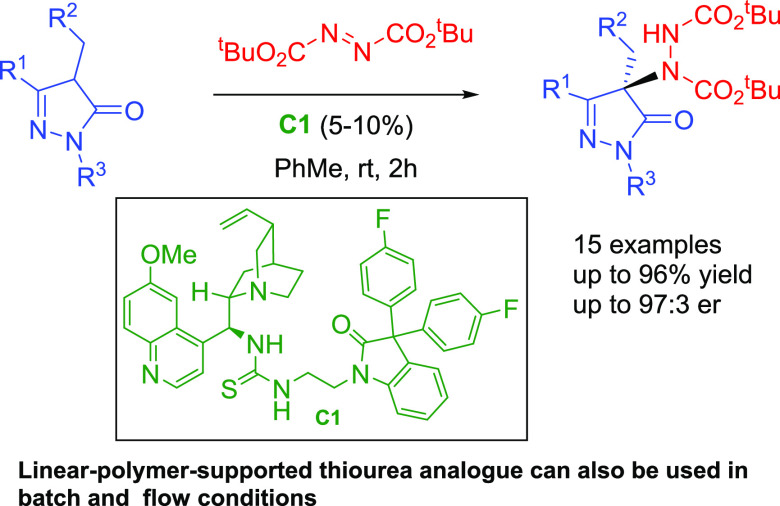

A highly efficient organocatalytic amination of 4-substituted pyrazolones with azodicarboxylates mediated by a novel quinine-derived thiourea with a 3,3-diaryl-oxindole scaffold is reported. This synthetic method furnished 4-amino-5-pyrazolones in high yields and with excellent enantioselectivities (up to 97:3 er) at room temperature in short reaction times. Moreover, a linear-polymer-supported bifunctional thiourea, synthesized by reacting a bifunctional aromatic monomer (biphenyl) with isatin in superacidic media and further derivatization, was proven to be also an efficient heterogeneous organocatalyst for this α-amination reaction. The practical value of this process was demonstrated by the use of the immobilized catalyst in recycling experiments, maintaining the activity without additional reactivation, and in flow processes, allowing the synthesis of 4-amino-pyrazolone derivatives in a gram scale with high yield and enantioselectivity.

## Introduction

**Figure 1:**
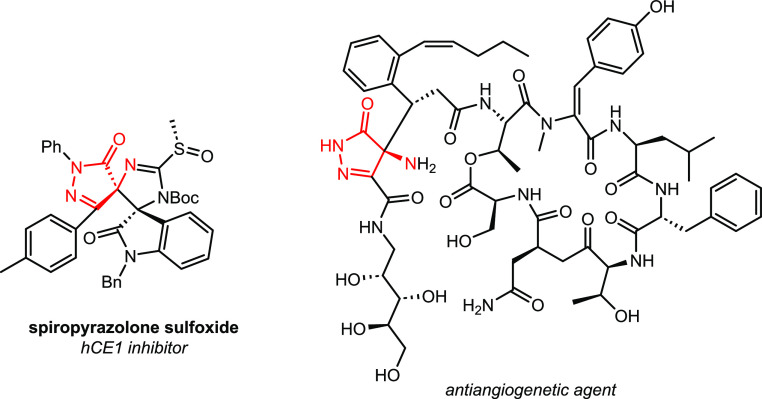
Biologically active 4-amino-5-pyrazolone derivatives.

Pyrazoles and pyrazolones constitute a privileged class of five-membered aza-heterocycles. Although they are not common components of biologically active natural products, they exhibit significant pharmacological activities.^1^ In addition, scaffolds with chiral α-tertiary amines are structural elements of a wide variety of natural products, bioactive molecules, pharmaceuticals, and agrochemicals.^2^ Considering the importance of pyrazolones and chiral α-tertiary amines, the development of new methods for the enantioselective synthesis of hybrid molecules that incorporate these two relevant motifs is expected to provide new compounds with significant biological activity. However, only a few examples of chiral amino-pyrazolones are documented despite their potential (Figure 1).^3^

In recent years, great research efforts have been focused on the development of new strategies for the enantioselective synthesis of chiral 4-amino-5-pyrazolones with a quaternary carbon stereocenter at C-4. Most of these methods utilized *N*-Boc pyrazolinone ketimines synthesized by Enders et al. as electrophiles in asymmetric Strecker,^4^ Mannich,^5^ or aza-Friedel–Crafts^6^ reactions. However, the organocatalytic electrophilic α-amination of 4-substituted pyrazolones is probably the most direct access to these compounds, but this protocol has been scarcely studied. Feng and co-workers reported in 2011 the organometallic enantioselective α-amination of 4-substituted pyrazolones with azodicarboxylates catalyzed by a chiral gadolinium complex.^7^ Later, Rios et al. developed the first organocatalytic amination of pyrazolones with diisopropyl azodicarboxylate catalyzed by quinine.^8^ This procedure requires low temperatures (−40 °C) and 2–3 days of reaction time to achieve high conversions and enantioselectivities, so more efficient organocatalysts would be desirable. All the described procedures have been performed under homogeneous conditions, and the recovery of the catalysts presents problems associated with chromatographic separations. It would be useful to have highly efficient heterogeneous organocatalysts, which would allow a more environmentally friendly approach to the α-amination reaction.^9^ In the literature, some examples of linear polymer-supported bifunctional thioureas are reported in the α-amination reaction of 3-aryl-2-oxindoles with azodicarboxylates in batch and flow conditions.^10^

We report herein our results on the α-amination of 4-substituted pyrazolones with di-*tert*-butyl azodicarboxylate catalyzed by homogeneous quinine-derived organocatalysts containing an oxindole moiety. In addition, as a part of our program directed to the synthesis of easily recoverable and reusable chiral bifunctional organocatalysts,^11^ we summarize here the preparation of a novel linear polymer-supported bifunctional thiourea derived from quinine by functionalization of a linear soluble polymer support formed by the superacid-promoted reaction of biphenyl and isatin and its use in the α-amination reaction. The strategy employed in the synthesis of this polymeric material has been used by Lozano et al. in the preparation of linear polymers (LPs) and porous organic polymers (POPs) used for carbon capture and gas separation applications and as supports for Pd(II) complexes and aminocatalysts.^12^

## Results and Discussion

**Scheme 1:**
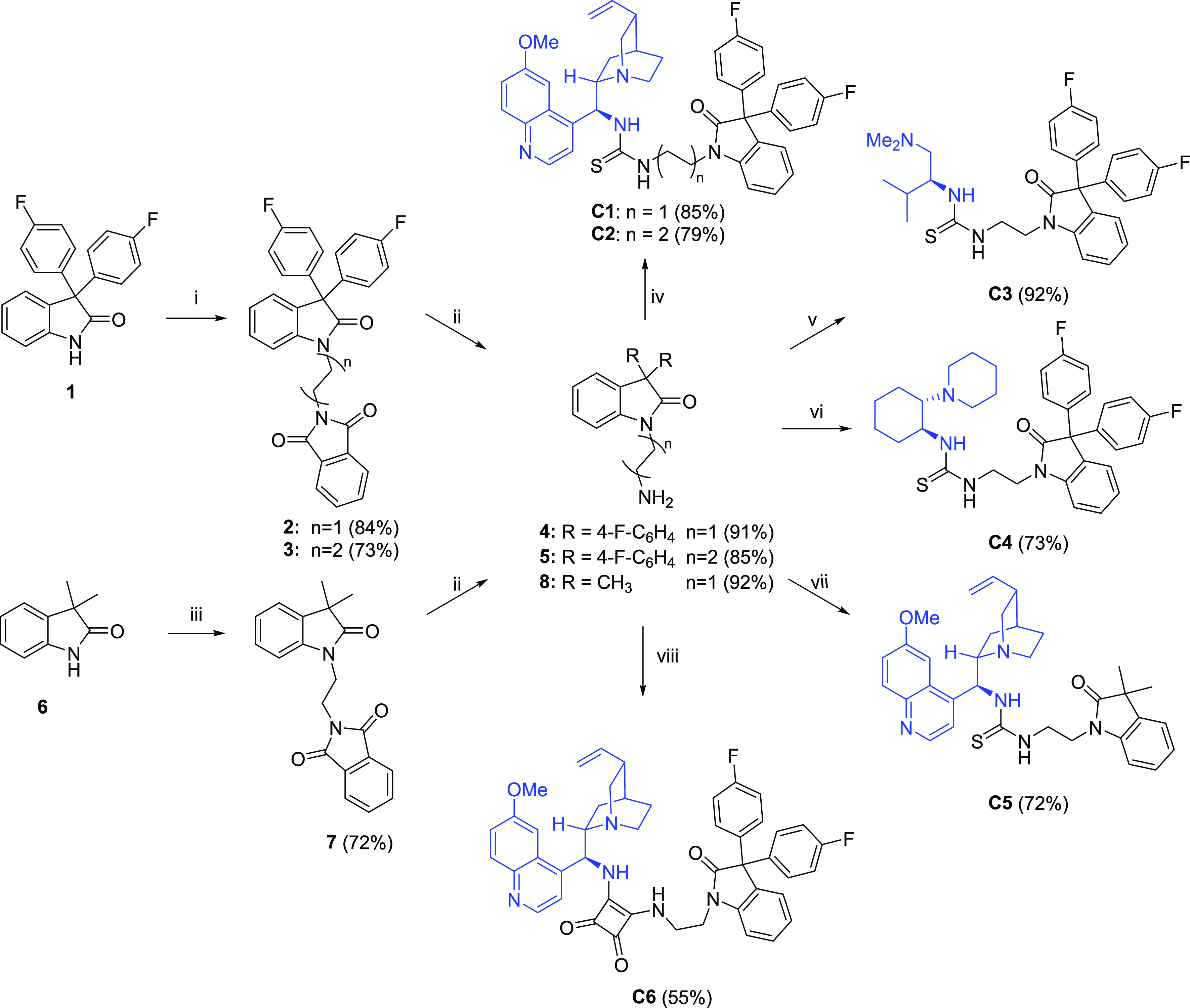
Synthesis of Catalysts **C1**–**C6** Reagents and conditions: (i) *N*-(2-bromoalkyl)-2-phthalimide (1.5 equiv), K_2_CO_3_ (1.5 equiv), DMF, 50 °C, 24 h. (ii) N_2_H_4_ (10.0 equiv), MeOH, 40 °C, 24 h. (iii) *N*-(2-bromoethyl)-2-phtalimide (1.5 equiv), NaH (1.5 equiv), DMF, rt. (iv–vii) R’NCS (1.0 equiv), DCM, rt, 24 h. (viii) QNA-semisquarate (1.0 equiv), MeOH, rt, 24 h.

Initially, a family of homogeneous oxindole-containing thioureas derived from quinine (QN), l-valine, and (1*R*,2*R*)-1, 2-cyclohexanediamine were synthesized (Scheme 1) for their use in the α-amination reaction of 4-substituted pyrazolones. 3,3-Diaryloxindole **1** was prepared by reaction of isatin with fluorobenzene in triflic acid by a modified procedure of Klumpp et al.^13^ Next, the synthesis of *N*-alkylphthalimido isatin derivatives **2** and **3** was accomplished in good yields by S_N_^2^ reaction of **1** with *N*-(2-bromoethyl)phthalimide or *N*-(4-bromobutyl)phthalimide using K_2_CO_3_ as a base in DMF at 50 °C. Subsequent removal of the phthalimide group from **2** and **3** by hydrazinolysis led to aminoalkyl derivatives **4** and **5**, having a two- and four-methylene spacer, respectively, in high yields. In a similar way, the synthesis of **8** was carried out from the commercial 3,3-dimethyl-oxindole (**6**). Finally, chiral bifunctional thioureas **C1**–**C5** were synthesized by condensation of amino derivatives **4**, **5**, and **8** with the appropriate isothiocyanates in DCM at room temperature. For comparative purposes, squaramide **C6** was also synthesized by condensation of **4** with 9-amino (9-deoxy)epi-quinine (QNA)-substituted semisquarate in moderate yield.

With this family of organocatalysts in hand, we tested their ability to promote the enantioselective α-amination reaction of 4-substituted pyrazolones with azodicarboxylates (Table 1).

**Table 1:**
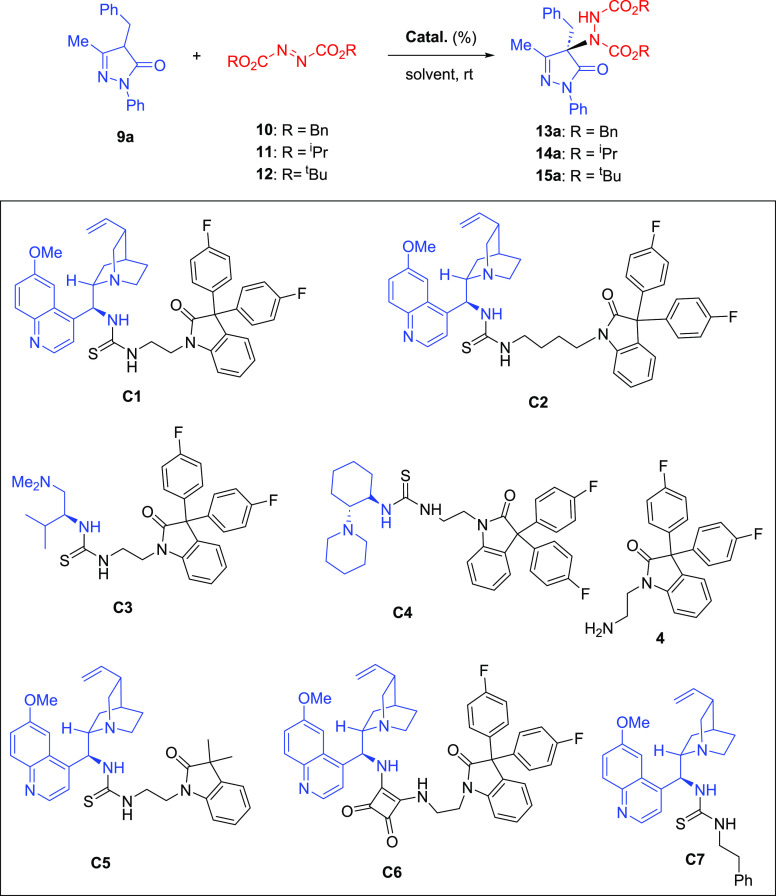
Catalyst Screening and Optimization of the Reaction Conditions

entry^a^	R	catalyst (%)	solvent	*t* (h)	yield^b^ (%)	er^c^
1	Bn	**C1** (10)	PhMe	1	89	84:16 (92:8)^d^
2	^i^Pr	**C1** (10)	PhMe	1	84	93:7 (92:8)^d^
3	^t^Bu	**C1** (10)	PhMe	1	85	95:5 (91:9)^d^
4	Bn	**C1** (5)	PhMe	1	87	82:18
5	^i^Pr	**C1** (5)	PhMe	1	83	93:7
6	^t^Bu	**C1** (5)	PhMe	1	85	94:6
7	^t^Bu	**C1** (10)	DCM	1	86	92:8
8	^t^Bu	**C1** (10)	THF	1	76	83:17
9	^t^Bu	**C1** (10)	2-MeTHF	1	81	86:14
10	^t^Bu	**C1** (10)	Cyrene	1	80	92:8
11^e^	^t^Bu	**C1** (10)	PhMe	4.5	80	94:6
12^f^	^t^Bu	**C1** (5)	PhMe	2	79	94:6
13	^t^Bu	**C2** (10)	PhMe	1	85	90:10
14	^t^Bu	**C3** (10)	PhMe	3.5	84	82:18
15	^t^Bu	**C4** (10)	PhMe	1	78	22:78
16	^t^Bu	**C5** (10)	PhMe	1	78	92:8
17	^t^Bu	**C6** (10)	PhMe	1	82	93:7
18	^t^Bu	**C7** (10)	PhMe	1	79	91:9
19	^t^Bu	**4** (10)	PhMe	1	85	50:50

a Reactions performed with pyrazolone **9a** (0.1 mmol), azodicarboxylate (0.12 mmol, 1.2 equiv), and the catalyst (5–10 mol %) in 1 mL of solvent at rt.

b Isolated yields.

c Determined by chiral HPLC analysis.

d Result obtained by Rios et al.^8a^ with quinine after 48 h at −40 °C.

e Reaction performed at −20 °C.

f Reaction performed with 1.0 equiv azodicarboxylate.

First, we investigated the reaction of 4-benzyl-5-pyrazolone **9a** with dibenzyl azodicarboxylate (**10**, 1.2 equiv) as the model reaction in the presence of 10 mol % of quinine-derived bifunctional thiourea **C1** in toluene at room temperature. The reaction was completed in 1 h, providing adduct **13a** in 89% yield and 84:16 er (entry 1). Interestingly, the use of diisopropyl azodicarboxylate (**11**, 1.2 equiv) for the same reaction produced adduct **14a** in 84% yield and higher enantiomeric ratio (93:7 er, entry 2) after 1 h reaction time. When the bulkier di-*tert-*butyl azodicarboxylate (DBAD) (**12**) was used as the amination reagent in the same reaction conditions, the enantiomeric ratio of **15a** increased up to 95:5 er (entry 3). To our delight, these results clearly improve the performance of the quinine used by Rios et al. that furnishes **14a** and **15a** with 92:8 and 91:9 er, respectively, after 48 h at −40 °C.^8a^ Moreover, the **C1** catalyst loading could be reduced to 5 mol % to achieve similar chemical yields and enantioselectivities after 1 h (entries 4–6). Screening of different solvents including DCM, THF, 2-MeTHF, and Cyrene showed that toluene was the best choice (entry 3 vs entries 7–10). However, it is worth highlighting the good enantiomeric ratio (92:8 er) obtained with Cyrene, an aprotic green alternative to common aprotic polar solvents that are of environmental concern (entry 10). Lowering the reaction temperature to −20 °C resulted in a longer reaction time and no improvement in the value of er (entry 11). The azodicarboxylate amount can also be reduced from 1.2 to 1.0 equiv with little change in either enantioselectivity or reaction time (entry 12).

Next, the performance of the rest of the synthesized organocatalysts in the asymmetric electrophilic amination of **9a** was evaluated. Quinine-derived thiourea **C2**, which contains a four-methylene spacer, afforded the desired product **15a** in good yield but lower enantiomeric ratio (90:10 er, entry 13). A significant decrease in enantioselectivity was also observed by using l-valine-derived thiourea **C3** (82:18 er, entry 14). (1*R*,2*R*)-Cyclohexanediamine-derived thiourea **C4** also effectively catalyzed this reaction but gave the opposite enantiomer of **15a** with a similar yield and lower selectivity (22:78 er, entry 15). Quinine-derived thiourea **C5** with a 3,3-dimethyl-oxindole scaffold did not improve the enantioselectivity either, which highlights the beneficial effect of diaryl geminal substitution at the C-3 position of the oxindole (92:8 er, entry 16). Quinine-derived squaramide **C6** was also less effective than the analogous thiourea **C1** (see entries 3 and 17). Finally, the beneficial effect of the 3,3-diaryloxindole scaffold on the selectivity of the catalyst was further demonstrated in the experiment with the quinine-derived thiourea **C7**, with a phenethyl group, which performed significantly worse than **C1** (compare entries 3 and 18). Interestingly, the achiral ethylamino derivative **4** also catalyzed the reaction and gave the racemic adduct **15a** in good yield, employing the same reaction time. This result showed that, in the presence of the aminoalkyl derivative **4**, the reaction proceeds efficiently in a nonstereoselective manner.

**Scheme 2:**
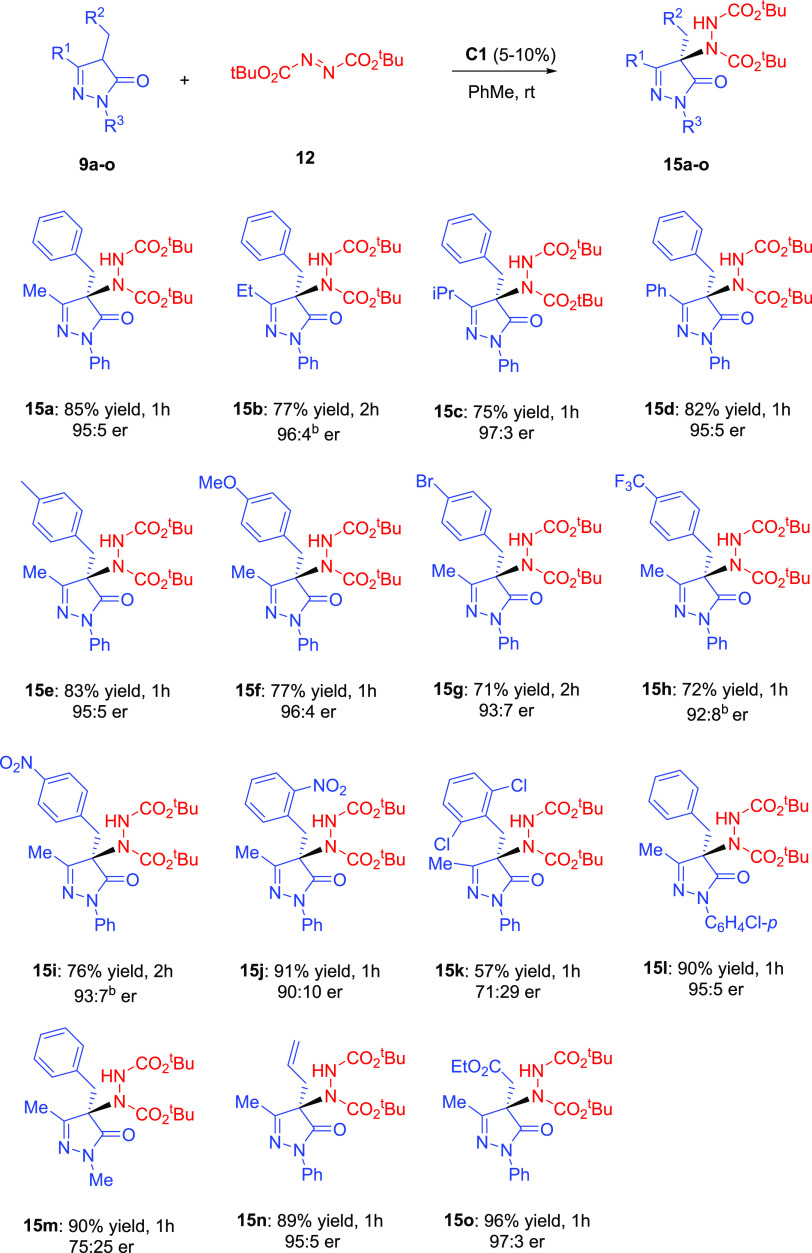
Scope of the Amination of 4-Substituted-5-pyrazolones with Catalyst **C1** Reactions performed with pyrazolone **9** (0.1 mmol), azodicarboxylate **12** (0.12 mmol, 1.2 equiv), and catalyst **C1** (10 mol %) in 1 mL of PhMe at rt. Yields correspond to isolated compound after flash chromatography. The er values were determined by chiral HPLC analysis. Reactions performed with 5 mol % catalyst **C1**.

With the optimized conditions in hand (5–10 mol % **C1** as a catalyst, toluene as a solvent, and room temperature), the substrate scope of the reaction was studied. The results are collected in Scheme 2. 4-Benzyl-5-pyrazolones **9a**–**9d**, with different substituents at the C-3 position (R^1^, Scheme 2), were first evaluated. An increase in the steric bulk at the alkyl substituent at the C-3 position resulted in slightly higher enantioselectivities, obtaining the best result for the isopropyl derivative **15c** (97:3 er). The reaction also tolerates aromatic rings at the C-3 position, and the phenyl-derived adduct **15d** was isolated in high yield and 95:5 er after 1 h of reaction. Next, *para*-substituted 4-benzyl-5-pyrazolones **9e**–**9i** were considered, and gratifyingly, good results were achieved for the products **15e**–**15i**. However, substrates having electron-withdrawing groups afforded the products **15g**–**15i** with somewhat lower enantiomeric ratios. Interestingly, the presence of substituents at the *ortho-*position of the phenyl group barely influences the enantioselectivity of the reaction (**15j**, 90:10 er). However, pyrazolone **9k**, with a sterically demanding 2,6-disubstituted-phenyl group, led to a dramatic decrease in enantioselectivity (71:29 er). When using pyrazolones with a different aryl group at the N-1 position, like a *p*-chlorophenyl group in **9l**, no change was observed in chemical yield and enantiomeric ratio. However, a remarkable decrease in enantioselectivity (75:25 er) was observed when the pyrazolone **9m**, *N*-methyl-substituted, was reacted with DBAD in the same reaction conditions. Finally, pyrazolones **9n** and **9o** bearing an allyl or ethoxycarbonyl methyl group at C-4 also provided the desired products in good yields and high enantioselectivities (95:5 and 97:3 er).

The absolute configuration of product **15a** was established to be *R* by comparison of the sign of the specific rotation and HPLC retention times with those previously described by Rios et al.^8a^ The absolute configuration of products **15b**–**15o** is expected to be the same by analogy assuming a common reaction pathway.

**Scheme 3:**
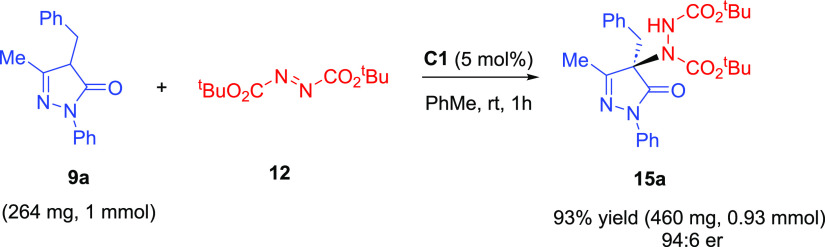
Scale-Up Reaction of **9a** with **12**

The developed protocol is amenable for a scale-up reaction. When 1.0 mmol of the pyrazolinone **9a** was reacted with di-*tert*-butyl azodicarboxylate in the presence of 5 mol % **C1** under the standard conditions, the desired product **15a** was obtained with a slightly improved yield and the same level of enantioselectivity (Scheme 3).

**Scheme 4:**
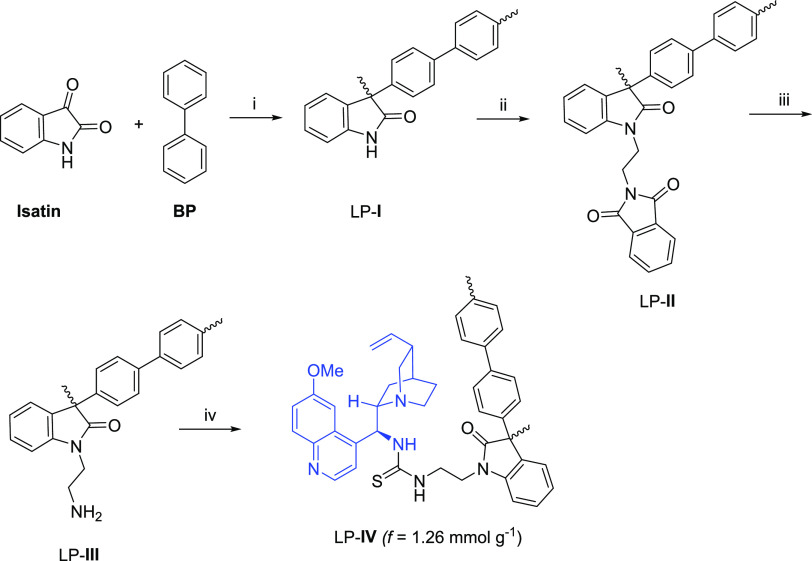
Synthesis of Polymers **I**–**IV** Reagents and conditions: (i) TFSA (10.0 equiv), CHCl_3_, 10 h, rt. (ii) *N*-(2-bromoethyl)-2-phthalimide (1.5 equiv), K_2_CO_3_ (1.5 equiv), NMP, 60 °C, 72 h. (iii) N_2_H_4_· H_2_O, NMP, 40 °C, 24 h. (iv) QN-NCS (1.5 equiv), DMSO, 50 °C, 72 h.

After having explored the scope and limitations of the homogeneous oxindole-containing thiourea organocatalysts, we proceeded to prepare a heterogeneous analogue of catalyst **C1** that would allow its use in the enantioselective amination of pyrazolones in both batch and continuous flow conditions (Scheme 4).

The precursor linear polymer (LP-**I**) was synthesized following Zolotukhin et al.’s methodology^12a,14^ by superacid-catalyzed polymerization of isatin with biphenyl (BP) employing a stoichiometric ratio of functional groups (1:1) and triflic acid (TFSA) as a reaction promoter, as depicted in Scheme 4. The polycondensation reaction proceeded in quantitative yield, and polymer LP-**I** was isolated as white threads. Then, LP-**I** was easily converted to polymer LP-**II** through S_N_^2^ reaction with *N*-(2-bromoethyl)phthalimide using K_2_CO_3_ as a base in NMP at 60 °C. This material was isolated as a white powder functionalized at 65% according to ^1^H NMR experiments. Next, hydrazine hydrate was used to deprotect the phthalimide group in NMP at 40 °C, affording LP-**III** functionalized with aminoethyl groups. Finally, owing to the solubility of LP**-III**, quinine-derived thiourea LP-**IV** was prepared by reaction of LP-**III** with isothiocyanate **QN-NCS** in DMSO at 50 °C. The effective functionalization (*f*) of the immobilized thiourea was 1.26 mmol g^–1^, based on sulfur elemental analysis.

To our delight, the isolated polymers LP-**I**–LP-**IV** were soluble in aprotic polar organic solvents (DMSO and DMAc), their chemical structures were characterized by solution NMR and ATR-FT-IR spectroscopy, and their thermal stabilities were studied via dynamic TGA experiments (see the Supporting Information). Furthermore, the inherent viscosity was determined for both LP-**I** and LP-**IV**. Supported bifunctional thiourea LP-**IV** exhibited excellent chemical and thermal stability due to the absence of chemically labile units and could be used as a heterogeneous catalyst due to its poor solubility in most conventional organic solvents.

Then, the activity of thiourea LP**-IV** as a heterogeneous catalyst was tested in the asymmetric α-amination of pyrazolone **9a** with di-*tert*-butyl azodicarboxylate in toluene at room temperature (Table 2). Satisfyingly, a catalyst loading of 20 mol % of LP-**IV** led to full conversion (determined by ^1^H NMR of the crude reaction mixture) in 2 h, and the product **15a** was isolated in high yield, albeit with somewhat reduced enantioselectivity relative to the homogeneous catalyst **C1** (90%, 88:12 er, entry 1). The catalyst loading could be reduced to 10 mol % to achieve a similar yield of **15a** after 3 h in toluene or in a 1:1 mixture of PhMe-DCM, but a new erosion in the enantioselectivity was observed (entries 2 and 3). As expected, polymer LP-**III** also promoted the amination reaction, leading to the racemic product (entry 4). The decrease in the enantioselectivity in the reaction promoted by the immobilized catalyst may be due to the presence of free aminoethyl groups and also to the different degrees of swelling, which is lower in toluene than in DCM (see the Supporting Information). For comparative purposes, a new assay was performed with 20 mol % of the known polystyrene-supported quinine-derived thiourea **V**(11a) under the same reaction conditions and adduct **15a** was isolated with a lower enantioselectivity (85:15 er, entry 5). This result again highlights the beneficial effect of the polymer structure of LP-**IV** on the enantioselective amination.

**Table 2:**
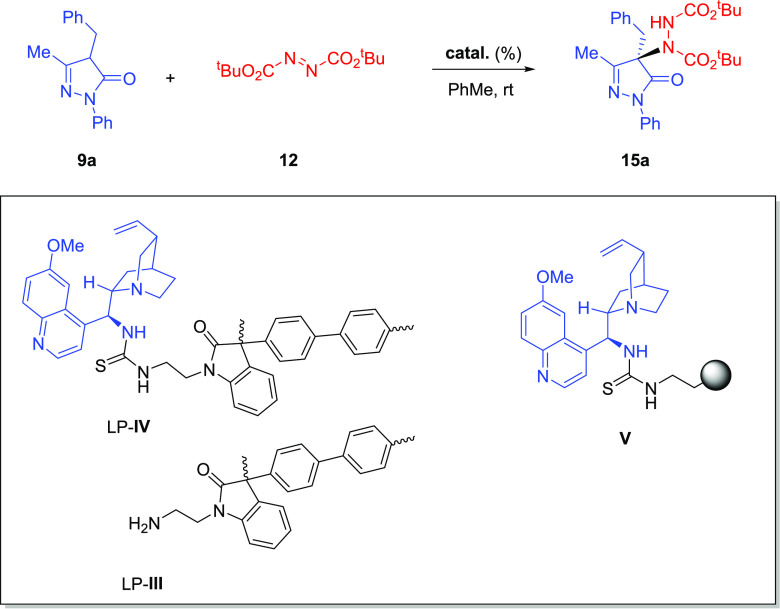
Amination of Pyrazolone **9a** with Supported Catalysts and Recycling Experiments^a^

**entry**	**catalyst (%)**	***t* (h)**	**yield (%)**^b^	**er**^c^
1	LP-**IV** (20)	2	90	88:12
2	LP-**IV** (10)	3	80	84:16
3^d^	LP-**IV** (10)	3	83	83:17
4	LP-**III** (20)	2	85	50:50
5	**V** (20)	6	82	85:15
6^e^	LP-**IV** (20)	2	95	87:13
7^e^	LP-**IV** (20)	2	88	87:13
8^e^	LP-**IV** (20)	2	83	87:13
9^e^	LP-**IV** (20)	2	83	87:13
10^e^	LP-**IV** (20)	2	87	86:14

a Reactions performed with pyrazolone **9a** (0.1 mmol), azodicarboxylate **12** (0.12 mmol, 1.2 equiv), and the catalyst (10–20%) in 1 mL of solvent at rt.

b Isolated yields.

c Determined by chiral HPLC analysis.

d Reaction performed in a 1:1 mixture of PhMe and DCM.

e Entries 6–10 correspond to the recycling experiments (2–6) for entry 1.

The heterogeneous character of the immobilized catalysts permitted their easy recovery and reuse. In particular, thiourea LP-**IV** was recovered by centrifugation, washed with toluene, and reused for six reaction cycles (entries 1 and 6–10), maintaining its activity without any significant loss of enantioselectivity.

To further explore the applicability of the novel immobilized thiourea LP-**IV** as an enantioselective catalyst, we focused our attention on the continuous process. Recently, these processes have attracted great attention within the pharmaceutical industry due to the advantages that they present with respect to the same reactions made in batch conditions, such as increased efficiency and sustainability.^15^ The system for the flow process was composed of an Omnifit chromatography column (6.6 mm ID) packed with supported catalyst LP-**IV** (300 mg, *f* = 1.26 mmol g^–1^) connected to a THALESNano micro HPLC pump (Table 3). Due to the low solubility of pyrazolone **9a** in toluene, a 1:1 mixture of toluene and dichloromethane was flushed for 60 min at 0.2 mL/min flow rate to swell the catalyst, and then an equimolar mixture of **9a** and **12** in the same mixture of solvents (unreactive in the absence of catalyst) was pumped through the reactor at 0.1 mL/min flow rate.

**Table 3:**
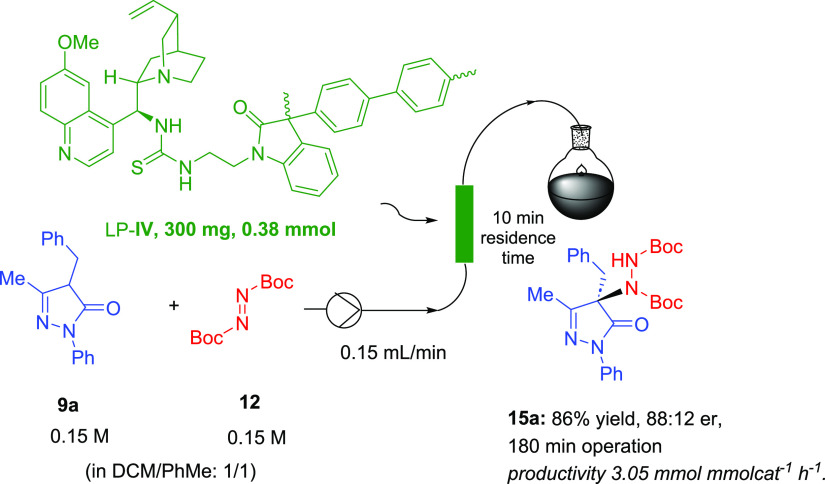
α-Amination of Pyrazolone **9a** with Azodicarboxylate **12** in Flow Conditions

**entry**	**flow**(mL/min)	***t* (min)**	***C***^a^**(M)**	**residence time (min)**	**Conv.**^b^**(yield)**^c^	**er**^d^
1	0.1	60	0.1	15	100 (87)	88:12
2	0.1	60	0.2	10	100 (83)	89:11
3	0.2	75	0.2	8	100 (85)	88:12
4	0.15	180	0.15	10	100 (86)	88:12

a Molar concentration of reactions performed with pyrazolone **9a** (0.1 mmol) and azodicarboxylate **12** (0.1 mmol, 1.0 equiv).

b Determined by ^1^H NMR in the reaction mixture.

c Isolated yield after purification by flash chromatography.

d Determined by HPLC on a chiral column.

First, we studied the influence of the substrate concentration in the continuous flow amination process. To this end, 1:1 mixtures of pyrazolone **15a** and azodicarboxylate **12** of different concentrations (0.1 and 0.2 M) in toluene-DCM (6 mL) were injected (0.1 mL/min, residence time: 10 min) in the column, and product **15a** was collected (Table 3, entries 1 and 2). The solid phase was washed with toluene for 30 min after each injection. Fortunately, increasing the concentration of reagents from 0.1 to 0.2 M did not modify either the conversion or the enantiomeric ratio of amination product **15a**. Then, the effect of the flow rate on the reaction was also studied. Full conversion was achieved, and no change in the enantioselectivity was observed with an increase in flow rate up to 0.2 mL/min (entry 3).

Under the compromise reaction conditions (entry 4 in Table 3), we decided to scale-up the continuous-flow process to prepare enantioenriched **15a** in a gram scale. A mixture of 1.07 g of **9a** and 0.93 g of **12** in 27 mL of toluene/DCM 1:1 (0.15 M) was pumped through the previous column for 3 h (0.15 mL/min). The process was monitored by ^1^H NMR (conversion) and HPLC on a chiral column (enantioselection), and to our delight, both conversion and enantiomeric ratio remained high throughout the process (after 3 h: 100% conversion, 88:12 er). The final mixture was purified by flash chromatography to yield the desired product **15a** in 86% isolated yield (1.72 g, 3.48 mmol) and good enantioselectivity (88:12 er). The data correspond to an effective catalyst loading of 9 mol %, an accumulated TON of 9.1, and a productivity of 3.05 mmol mmolcat^–1^ h^–1^ for the synthesis of **15a**. The residence time, under these flow conditions, was 10 min, in sharp contrast with the reaction time required for full conversion in batch operation (3 h). Moreover, the enantioselection in the flow experiments was better than that obtained in the batch reaction under similar conditions (see entry 3, Table 2).

**Scheme 5:**
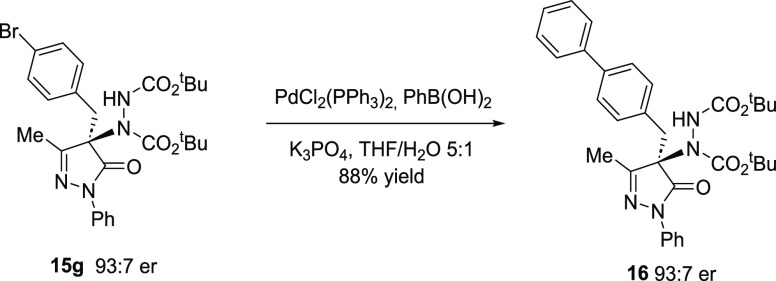
Synthetic Transformation of Pyrazolone Adduct **15g**

To demonstrate the synthetic utility of our method, the preparation of biphenyl derivative **16** was achieved via a Pd-catalyzed Suzuki coupling of bromide **15g** with phenylboronic acid (Scheme 5). The reaction progressed to deliver the desired product in 88% yield with retention of enantiopurity.

## Conclusions

In summary, a new family of homogeneous oxindole-containing catalysts derived from quinine, L-valine, and (1*R*,2*R*)-1,2-cyclohexanediamine was synthesized for their use in the enantioselective amination of 4-substituted pyrazolones with azodicarboxylates at room temperature. The novel quinine-derived bifunctional thiourea **C1** with a 3,3-diaryl-oxindole scaffold was the most promising catalyst, which was able to efficiently catalyze the enantioselective amination to obtain a wide library of aminopyrazolones with very good yields and enantioselection. The immobilized catalyst analogue LP-**IV** has also been prepared and could be recycled and reused without loss of activity in batch (six cycles) and continuous-flow (four runs) conditions. The described protocol constitutes a significant improvement since only a 10 min residence time is required for the preparation of chiral 4-amino-pyrazolones in a gram scale with very good yields and enantioselectivity, showing the potential value of this catalyst.

## Experimental Section

### General Information

^1^H NMR (400 or 500 MHz) and ^13^C{^1^H} NMR (100 or 126 MHz) spectra were recorded in CDCl_3_ or DMSO-*d*_6_ as a solvent. Chemical shifts for protons are reported in ppm from TMS with the residual CHCl_3_ resonance as an internal reference. Chemical shifts for carbons are reported in ppm from TMS and are referenced to the carbon resonance of the solvent. Data are reported as follows: chemical shift, multiplicity (s = singlet, d = doublet, t = triplet, q = quartet, m = multiplet, br = broad), coupling constants in Hertz, and integration.

Specific rotations were measured on a PerkinElmer 341 digital polarimeter using a 1 mL cell with a 1 dm path length and a sodium lamp, and concentration is given in g per 100 mL. Infrared spectra were recorded on a PerkinElmer Spectrum One FT-IR spectrometer and are reported in frequency of absorption (only the structurally most important peaks are given). Flash chromatography was carried out using silica gel (230–240 mesh). TLC analysis was performed on glass-backed plates coated with silica gel 60 and an F254 indicator and visualized by either UV irradiation or by staining with phosphomolybdic acid or potassium permanganate solutions. Melting points were obtained with open capillary tubes and are uncorrected. Chemical yields refer to pure isolated substances.

Chiral HPLC analysis was performed on a JASCO HPLC system (JASCO PU-2089 pump and UV-2075 UV/vis detector) and on a Hewlett-Packard 1090 Series II instrument equipped with a quaternary pump using Phenomenex Lux i-Cellulose-5 and Phenomenex Lux i-Amylose-1 analytical columns (250 × 4.6 mm). Detection was monitored at 254 nm. Elemental analyses were carried out at the Elemental Analysis Center of the Complutense University of Madrid, using an LECO CHNS-932. ESI mass spectra were obtained on an Agilent 5973 inert GC/MS system. Thermogravimetric analysis (TGA) was performed on a TG-Q500 analyzer at the ICTP-CSIC Center using nitrogen gas flow (60 mL/min). The samples were heated at 20 °C/min from 30 to 850 °C using the Hi-Res method, with sensitivity and resolution parameters of 1 and 4. Inherent viscosities were measured at the ICTP-CSIC using a Lauda iVisc device and an Ubbelohde viscometer. The viscosities of the polymers were measured at 30 °C using *N,N*-dimethylacetamide (DMAc) as a solvent at 0.5 g/dL concentration.

Commercially available reagents were used as purchased without further treatment. Solvents were dried and stored over microwave-activated 4 Å molecular sieves. *N*-2-Bromoethyl-2-phftalimide and *N*-2-bromobutyl-2-phftalimide,^16^ 4-substituted pyrazolones,^8^ thiourea **V**(11a) (*f* = 0.75 mmol g^–1^), (8*a*,9*S*)-9-isothiocyanato-6′-methoxycinchonan (QN-NCS),^17^ (2*S*)-2-isothiocyanato-*N,N*,3-trimethyl-1-butanamine,^17^ and 1-[(1*R*,2*R*)-2-isothiocyanatocyclohexyl]piperidine^18^ were prepared as previously described. Racemic reference samples were prepared using an achiral bifunctional thiourea derived from *N*^1^,*N*^1^-dimethylethane-1,2-diamine^19^ as a catalyst.

#### 3,3-Bis(4-fluorophenyl)indolin-2-one (**1**)^13^

In an oven-dried Schlenk equipped with a magnetic stirrer and blanketed by a nitrogen atmosphere, isatin (3.0 g, 20.4 mmol) was dissolved in anhydrous CHCl_3_ (45 mL) and fluorobenzene (4.2 mL, 44.8 mmol, 2.2 equiv) was added. The solution was placed into an ice bath, then TFSA (30 mL) was added dropwise for 30 min, and the mixture was stirred at room temperature for 24 h. The dark solution was poured into cold distilled water, and the white precipitate was collected, washed with warm distilled water, and used without further purification. White solid (5.4 g, 16.9 mmol, 83% yield). ^1^H NMR (500 MHz CDCl_3_): δ 8.24 (s, 1H), 7.28–7.20 (m, 5H), 7.18 (d, *J* = 7.5 Hz, 1H), 7.08 (td, *J* = 7.6, 1.0 Hz, 1H), 7.02–6.95 (m, 5H) ppm. ^13^C{^1^H} NMR (101 MHz, CDCl_3_) δ 180.1, 162.2 (d, ^1^*J*_C–F_ = 247.2 Hz, 2C), 140.1, 137.2 (d, ^4^*J*_C–F_ = 3.3 Hz, 2C), 133.2, 130.1 (d, ^3^*J*_C–F_ = 8.1 Hz, 4C), 128.7, 126.1, 123.1, 115.1 (d, ^2^*J*_C–F_ = 21.5 Hz, 4C), 110.7, 61.5 ppm. ^19^F NMR (376 MHz, CDCl_3_) δ −114.8 ppm.

#### 2-(2-(3,3-Bis(4-fluorophenyl)-2-oxoindolin-1-yl)ethyl)isoindoline-1,3-dione (**2**)

To a suspension of **1** (0.96 g, 3.0 mmol) and anhydrous K_2_CO_3_ (0.62 g, 4.5 mmol, 1.5 equiv) in DMF (25 mL) was added *N*-2-bromoethyl-2-phftalimide (1.14 g, 4.5 mmol, 1.5 equiv), and the mixture was stirred at 50 °C for 24 h. The reaction mixture was poured over water (100 mL) and extracted with dichloromethane (3 × 25 mL). The combined organic layers were dried with anhydrous MgSO_4_ and filtered, and the organic solvent was evaporated under reduced pressure. The residue was purified by flash chromatography (hexane/EtOAc: 4/1) to afford pure product **2** as a green pale solid (1.24 g, 2.5 mmol, 84%). Mp 174–176 °C. ^1^H NMR (400 MHz CDCl_3_) δ 7.69–7.61 (m, 4H), 7.30–7.26 (m, 2H), 7.17 (d, *J* = 7.5 Hz, 1H), 7.13–7.05 (m, 4H), 7.02 (d, *J* = 7.9 Hz, 1H), 6.88 (t, *J* = 8.6 Hz, 4H), 4.14 (t, *J* = 5.7 Hz, 2H), 4.05–4.00 (m, 2H) ppm. ^13^C{^1^H} NMR (101 MHz, CDCl_3_) δ 177.6, 167.8 (2C), 161.9 (d, ^1^*J*_C–F_ = 246.7 Hz, 2C), 141.7, 137.2 (d, ^4^*J*_C–F_ = 3.2 Hz, 2C), 133.8 (2C), 132.3, 131.6, 130.1 (d, ^3^*J*_C–F_ = 8.1 Hz, 4C), 128.7, 126.2, 123.3 (2C), 123.1 (2C), 115.2 (d, ^2^*J*_C–F_ = 21.5 Hz, 4C), 108.5, 60.8, 38.5, 35.2 ppm. ^19^F NMR (470 MHz, CDCl_3_) δ −115.2 ppm. IR (ATR): 2963, 2923, 2853, 1776, 1707, 1604, 1509, 1468, 1318, 1402, 1219, 1164, 831, 710 cm^–1^. HRMS (ESI-QTOF) *m*/*z*: [M + Na]^+^ Calcd for C_30_H_20_F_2_N_2_NaO_3_ 517.1334; Found 517.1342.

#### 2-(4-(3,3-Bis(4-fluorophenyl)-2-oxoindolin-1-yl)butyl)isoindoline-1,3-dione (**3**)

Compound **3** was obtained as described for **2** using **1** (0.96 g, 3.0 mmol), anhydrous K_2_CO_3_ (0.62 g, 4.5 mmol, 1.5 equiv), and *N*-2-bromobutyl-2-phftalimide (1.27 g, 4.5 mmol, 1.5 equiv). Purification by flash chromatography (hexane/EtOAc: 4/1) afforded the pure product as a colorless oil (1.15 g, 2.2 mmol, 73%). ^1^H NMR (400 MHz CDCl_3_,) δ 7.86–7.81 (m, 2H), 7.73–7.69 (m, 2H), 7.31 (td, *J* = 7.7, 1.2 Hz, 1H), 7.21–7.15 (m, 5H), 7.08 (td, *J* = 7.6, 0.8 Hz, 1H), 7.00–6.92 (m, 5H), 3.83 (t, *J* = 6.8 Hz, 2H), 3.71 (t, *J* = 6.8 Hz, 2H), 1.85–1.63 (m, 4H) ppm. ^13^C{^1^H} NMR (101 MHz, CDCl_3_) δ 177.3, 168.3 (2C), 162.1 (d, ^1^*J*_C–F_ = 246.8 Hz, 2C), 142.1, 137.4 (d, ^4^*J*_C–F_ = 3.3 Hz, 2C), 133.9 (2C), 132.7, 132.0, 129.9 (d, ^3^*J*_C–F_ = 8.2 Hz, 4C), 128.6, 126.0 (2C), 123.2, 122.9 (2C), 115.4 (d, ^2^*J*_C–F_ = 21.5 Hz, 4C), 109.0, 61.1, 39.7, 37.3, 26.0, 24.8 ppm. ^19^F NMR (376 MHz, CDCl_3_) δ −115.1 ppm. IR (ATR): 3410, 3059, 2934, 2861, 1769, 1710, 1608, 1509, 1490, 1461, 1439, 1399, 1351, 1227, 1153, 1090, 1043, 827 cm^–1^. HRMS (ESI-QTOF) *m*/*z*: [M + Na]^+^ Calcd for C_32_H_24_F_2_N_2_NaO_3_ 545.1647; Found 545.1661.

#### 2-(2-(3,3-Dimethyl-2-oxoindolin-1-yl)ethyl)isoindoline-1,3-dione (**7**)

To a suspension of **6** (0.48g, 3.0 mmol) and anhydrous NaH in 60% mineral oil (180 mg, 4.5 mmol, 1.5 equiv) in DMF (25 mL) was added *N*-(bromoethyl)-2-phtalimide (4.5 mmol, 1.5 equiv) at room temperature, and the mixture was stirred for 24 h. The reaction mixture was poured over water (100 mL) and extracted with dichloromethane (3 × 25 mL). The combined organic layers were dried with anhydrous MgSO_4_ and filtered, and the organic solvent was evaporated under reduced pressure. Purification by flash chromatography (hexane/EtOAc: 1/1) afforded the pure product as a white solid (0.72 g, 2.2 mmol, 72%). ^1^H NMR (500 MHz CDCl_3_) δ 7.80–7.75 (m, 2H), 7.70–7.65 (m, 2H), 7.17–7.14 (m, 1H), 7.11 (td, *J* = 7.7, 1.2 Hz, 1H), 6.98 (t, *J* = 7.5 Hz, 1H), 6.85 (d, *J* = 7.8 Hz, 1H), 4.06–3.98 (m, 4H), 1.29 (s, 6H) ppm. ^13^C{^1^H} NMR (126 MHz, CDCl_3_) δ 181.5, 168.1 (2C), 141.4, 135.8, 133.99, 133.94 (2C), 131.9, 127.5, 123.4, 123.2, 122.5 (2C), 107.5, 43.9, 38.3, 35.6, 24.1 (2C) ppm. IR (ATR): 1772, 1710, 1703, 1692, 1607, 1487, 1465, 1430, 1393, 1381, 1370, 1139, 1034, 1035, 743 cm^–1^. HRMS (ESI-QTOF) *m*/*z*: [M + Na]^+^ Calcd for C_20_H_18_N_2_NaO_3_ 357.1210; Found 357.1216.

### General Procedure for Hydrazinolysis of Compounds **2**, **3**, and **7**

The *N*-alkylphtalimide derivative (2.0 mmol) was dissolved in MeOH (20 mL), and hydrazine hydrate (20.0 mmol, 10.0 equiv) was added. The solution was heated at 40 °C for 24 h. The reaction mixture was poured over water (50 mL) and extracted with dichloromethane (3 × 25 mL). The combined organic layers were dried over anhydrous MgSO_4_ and filtered, and the organic solvent was evaporated under reduced pressure to afford the crude product, which was used without further purification.

#### 1-(2-Aminoethyl)-3,3-bis(4-fluorophenyl)indolin-2-one (**4**)

Compound **4** was prepared from **2** (0.99 g, 2.0 mmol) according to the general procedure as a yellow oil (0.66 g, 1.82 mmol, 91%). ^1^H NMR (400 MHz CDCl_3_) δ 7.32 (td, *J* = 7.7, 1.2 Hz, 1H), 7.25–7.18 (m, 5H), 7.10 (td, *J* = 7.6, 0.9 Hz, 1H), 7.01–6.94 (m, 5H), 3.86 (t, *J* = 6.4 Hz, 2H), 3.05 (t, *J* = 6.4 Hz, 2H), 1.48 (br s, 2H) ppm. ^13^C{^1^H} NMR (101 MHz, CDCl_3_) δ 177.9, 162.1 (d, ^1^*J*_C–F_ = 247.0 Hz, 2C), 142.2, 137.5 (d, ^4^*J*_C–F_= 3.3 Hz, 2C), 132.7, 130.0 (d, ^3^*J*_C–F_= 8.2 Hz, 4C), 129.9, 128.6, 126.1, 123.0, 115.4 (d, ^2^*J*_C–F_ = 21.5 Hz, 4C), 109.0, 43.4, 39.8 ppm. ^19^F NMR (376 MHz, CDCl_3_) δ −114.9 ppm. IR (ATR): 3381, 3059, 2923, 2857, 1703, 1604, 1509, 1490, 1347, 1223, 1157, 1095, 1010, 827, 812, 747 cm^–1^. HRMS (ESI-QTOF) *m*/*z*: [M + Na]^+^ Calcd for C_22_H_18_F_2_N_2_NaO 387.1279; Found 387.1278.

#### 1-(4-Aminobutyl)-3,3-bis(4-fluorophenyl)indolin-2-one (**5**)

Compound **5** was prepared from **3** (1.04 g, 2.0 mmol) according to the general procedure as a yellow oil (0.67 g, 1.70 mmol, 85%).^1^H NMR (400 MHz CDCl_3_) δ 7.34–7.32 (m, 2H), 7.22–7.16 (m, 4H), 7.12–7.06 (m, 1H), 7.01–6.92 (m, 5H), 3.79 (t, *J* = 7.4 Hz, 2H), 2.72 (t, *J* = 7.0 Hz, 2H), 1.81–1.72 (m, 2H), 1.52–1.43 (m, 2H), 1.38 (br s, 2H) ppm. ^13^C{^1^H} NMR (101 MHz, CDCl_3_) δ 177.2, 162.0 (d, ^1^*J*_C–F_= 246.9 Hz, 2C), 144.2, 137.6 (d, ^4^*J*_C–F_ = 3.2 Hz, 2C), 132.7, 130.0 (d, ^3^*J*_C–F_ = 8.1 Hz, 4C), 128.6, 126.0, 122.9, 115.4 (d, ^2^*J*_C–F_ = 21.5 Hz, 4C), 109.0, 61.1, 41.6, 40.9, 30.8, 24.8 ppm. ^19^F NMR (376 MHz, CDCl_3_) δ −115.0 ppm. IR (ATR): 3048, 2934, 2853, 1710, 1608, 1501, 1487, 1465, 1355, 1223, 1164, 1091, 1014, 907, 827, 812, 729 cm^–1^. HRMS (ESI-QTOF) *m*/*z*: [M + H]^+^ Calcd for C_24_H_23_F_2_N_2_NaO 393.1773; Found 393.1787.

#### 1-(2-Aminoethyl)-3,3-dimethylindolin-2-one (**8**)

Compound **8** was prepared from **7** (0.67 g, 2.0 mmol) according to the general procedure as a yellow oil (0.38 g, 1.84 mmol, 92%). ^1^H NMR (500 MHz, CDCl_3_): δ 7.23–7.15 (m, 2H), 7.02 (t, *J* = 7.5 Hz, 1H), 6.87 (d, *J* = 7.8 Hz, 1H), 3.75 (t, *J* = 6.5 Hz, 2H), 3.00–2.92 (m, 2H), 1.67 (br s, 2H), 1.34 (s, 6H) ppm. ^13^C{^1^H} NMR (126 MHz, CDCl_3_) δ 181.9, 141.8, 135.8, 127.6, 122.5 (2C), 108.3, 44.1, 43.0, 39.8, 24.5 (2C) ppm. IR (ATR): 3366, 2970, 2926, 2864, 1696, 1611, 1486, 1461, 1355, 1388, 1304, 1157, 1124, 937, 758, 743 cm^–1^. HRMS (ESI-QTOF) *m*/*z*: [M + H]^+^ Calcd for C_12_H_17_N_2_O 205.1335; Found 205.1343.

### General Procedure for the Synthesis of Bifunctional Thiourea Catalysts **C1**–**C5**

The *N*-aminoalkyl isatin derivative (**4**, **5**, and **8**) (0.55 mmol, 1.1 equiv) was dissolved in dichloromethane (10 mL), and the chiral amine-NCS (0.50 mmol, 1.0 equiv) was added. The reaction was stirred until the starting products were consumed (monitored by TLC). The solvent was removed under reduced pressure, and residue was purified by flash column chromatography (silica gel, eluent DCM/MeOH: 10/1) to afford the pure catalysts **C1**–**C5** in good yields.

#### 1-(2-(3,3-Bis(4-fluorophenyl)-2-oxoindolin-1-yl)ethyl)-3-((*S*)-(6-methoxyquinolin-4-yl)((1*S*,2*S*,4*S*,5*R*)-5-vinylquinuclidin-2-yl)methyl)thiourea (**C1**)

Catalyst **C1** was prepared according to the general procedure using **4** (200 mg, 0.55 mmol) and QN-NCS (183 mg, 0.5 mmol) as a white solid (310 mg, 0.43 mmol, 85% yield). Mp 155–158 °C.  = −44.1 [(*c* = 0.44, CHCl_3_)]. ^1^H NMR (400 MHz, CDCl_3_) δ 8.72 (m, 1H), 8.02 (m, 1H), 7.93 (m, 1H), 7.58 (m, 1H), 7.40 (dd, *J* = 9.3, 2.5 Hz, 1H), 7.23–7.04 (m, 6H), 6.94 (m, 6H), 6.45 (br s, 1H), 5.72–5.57 (m, 1H), 5.09 (m, 2H), 4.01 (m, 5H), 3.90–3.69 (m, 3H), 3.43 (m, 1H), 3.18 (m, 1H), 2.98 (m, 1H), 2.57 (m, 1H), 1.91 (m, 3H), 1.62 (m, 1H), 1.25 (m, 2H), 1.11 (m, 1H), 0.91–0.76 (m, 1H) ppm. ^13^C{^1^H} NMR (101 MHz, CDCl_3_) δ 183.0, 177.9, 171.1, 162.0 (d, ^1^*J*_C–F_ = 247.1 Hz, 2C), 158.4, 147.8, 144.9, 142.0, 137.3 (d, ^4^*J*_C–F_ = 3.2 Hz), 137.2 (d, ^4^*J*_C–F_’ = 3.2 Hz), 137.0, 132.4, 131.8, 130.14 (d, ^3^*J*_C–F_ = 8.0 Hz, 2C), 130.06 (d, ^3^*J*_C–F_’ = 8.0 Hz, 2C), 129.9, 128.6, 127.8, 125.7, 123.0, 122.4, 117.3, 115.9 (d, ^2^*J*_C–F_ = 21.5 Hz, 2C), 115.3 (d, ^2^*J*_C–F_’ = 21.5 Hz, 2C), 109.5, 102.3, 61.2, 60.4, 56.1, 53.9, 42.2, 39.5, 36.9, 29.7, 26.8, 24.9, 24.1, 14.2 ppm. ^19^F NMR (376 MHz, CDCl_3_) δ −115.0 ppm. IR (ATR): 3219, 2938, 1717, 1604, 1505, 1490, 1468, 1355, 1219, 1161, 1021, 915, 827, 750, 571, 516 cm^–1^. HRMS (ESI-QTOF) *m*/*z*: [M + H]^+^ Calcd for C_43_H_42_F_2_N_5_O_2_S 730.3022; Found 730.3051.

#### 1-(4-(3,3-Bis(4-fluorophenyl)-2-oxoindolin-1-yl)butyl)-3-((*S*)-(6-methoxyquinolin-4-yl)((1*S*,2*S*,4*S*,5*R*)-5-vinylquinuclidin-2-yl)methyl)thiourea (**C2**)

Catalyst **C2** was prepared according to the general procedure using **5** (216 mg, 0.55 mmol) and QN-NCS (183 mg, 0.5 mmol) as a pale-yellow solid (299 mg, 0.395 mmol, 79% yield).  = −33.8 [(*c* = 0.24, CHCl_3_)]. ^1^H NMR (400 MHz, CDCl_3_) δ 8.65 (d, *J* = 4.5 Hz, 1H), 7.98 (d, *J* = 9.2 Hz, 1H), 7.79 (br s, 1H), 7.56 (br s, 1H), 7.42–7.31 (m, 2H), 7.28–7.22 (m, 2H), 7.20–7.13 (m, 5H), 7.06 (m, 1H), 6.93 (m, 5H), 5.63 (m, 1H), 5.01–4.88 (m, 2H), 3.95 (s, 3H), 3.69 (m, 2H), 3.49–3.17 (m, 4H), 3.13–2.99 (m, 1H), 2.75–2.55 (m, 2H), 2.23 (m, 1H), 1.73–1.29 (m, 8H), 1.24 (s, 1H), 0.88 (m, 1H) ppm. ^13^C{^1^H} NMR (101 MHz, CDCl_3_) δ 182.4, 177.4, 162.05 (d, ^1^*J*_C–F_ = 247.0 Hz), 162.04 (d, ^1^*J*_C–F_’ = 247.0 Hz), 158.0, 147.6, 144.8, 141.9, 140.4, 137.5 (d, ^4^*J*_C–F_ = 3.3 Hz), 137.4 (d, ^4^*J*_C–F_’ = 3.3 Hz), 132.7, 131.7, 130.2 (d, ^3^*J*_C–F_ = 8.1 Hz, 2C), 129.9 (d, ^3^*J*_C–F_’ = 8.1 Hz, 2C), 128.9, 128.7, 127.9, 125.9, 123.0, 122.1, 115.45 (d, ^2^*J*_C–F_ = 21.5 Hz, 2C), 115.42 (d, ^2^*J*_C–F_’ = 21.5 Hz, 2C), 115.3, 115.1, 109.2, 102.3, 61.1, 60.7, 55.8, 55.1, 43.9, 41.2, 39.9, 38.9, 29.7, 27.21, 27.17, 26.3, 25.5, 24.7 ppm. ^19^F NMR (376 MHz, CDCl_3_) δ −114.8 ppm. IR (ATR):3223, 3069, 2930, 1707, 1600, 1545, 1505, 1490, 1461, 1355, 1304, 1223, 1164, 1091, 1029, 923, 824, 743 cm^–1^. HRMS (ESI-QTOF) *m*/*z*: [M + H]^+^ Calcd for C_45_H_46_F_2_N_5_O_2_S 758.3335; Found 758.3335.

#### (*S*)-1-(2-(3,3-Bis(4-fluorophenyl)-2-oxoindolin-1-yl)ethyl)-3-(1-(dimethylamino)-3-methylbutan-2-yl)thiourea (**C3**)

Catalyst **C3** was prepared according to the general procedure using **4** (200 mg, 0.55 mmol) and (2*S*)-2-isothiocyanato-*N,N*,3-trimethyl-1-butanamine (86 mg, 0.5 mmol) as a white solid (247 mg, 0.46 mmol, 92% yield). Mp 96–98 °C.  = −16.6 [(*c* = 0.74, MeOH)]. ^1^H NMR (500 MHz, CDCl_3_) δ 10.58 (br s, 1H), 7.40–7.29 (m, 2H), 7.24–7.15 (m, 5H), 7.07 (td, *J* = 7.5, 1.0 Hz, 1H), 6.99–6.93 (m, 4H), 5.93 (br s, 1H), 4.07 (t, *J* = 6.7 Hz, 2H), 4.00–3.83 (m, 2H), 3.73 (m, 1H), 3.28–2.89 (m, 1H), 2.34–1.91 (m, 7H), 1.80 (m, 1H), 0.92 (d, *J* = 6.9 Hz, 3H), 0.90 (d, *J* = 6.8 Hz, 3H) ppm. ^13^C{^1^H} NMR (101 MHz, CDCl_3_) δ 184.0, 177.7, 162.1 (d, ^1^*J*_C–F_ = 247.1 Hz, 2C), 142.4, 137.5 (d, ^4^*J*_C–F_ = 3.3 Hz), 137.4 (d, ^4^*J*_C–F_’ = 3.3 Hz), 132.3, 130.05 (d, ^3^*J*_C–F_ = 8.1 Hz, 2C), 130.00 (d, ^3^*J*_C–F_’ = 8.1 Hz, 2C), 128.8, 125.7, 123.0, 115.42 (d, ^2^*J*_C–F_ = 21.5 Hz, 2C), 115.37 (d, ^2^*J*_C–F_’ = 21.5 Hz, 2C), 110.1, 61.1, 44.8 (2C), 42.9, 39.6, 39.4, 31.7, 29.7, 18.2, 18.0 ppm. ^19^F NMR (376 MHz, CDCl_3_) δ −114.9 ppm. IR (ATR): 3245, 2963, 1714, 1607, 1542, 1505, 1489, 1461, 1355, 1227, 1160, 1095, 1018, 827, 754 cm^–1^. HRMS (ESI-QTOF) *m*/*z*: [M + H]^+^ Calcd for C_30_H_35_F_2_N_4_OS 537.2494; Found 537.2504.

#### 1-(2-(3,3-Bis(4-fluorophenyl)-2-oxoindolin-1-yl)ethyl)-3-((1*R*,2*R*)-2-(piperidin-1-yl)cyclohexyl)thiourea (**C4**)

Catalyst **C4** was prepared according to the general procedure using **4** (200 mg, 0.55 mmol) and 1-[(1*R*,2*R*)-2-isothiocyanatocyclohexyl]piperidine (112 mg, 0.5 mmol) as a white solid (215 mg, 0.365 mmol, 73% yield). Mp 115–120 °C.  = −11.25 [(*c* = 0.80, CHCl_3_)]. ^1^H NMR (500 MHz, CDCl_3_) δ 7.31 (td, *J* = 7.7, 1.2 Hz, 1H), 7.23 (m, 1H), 7.18 (m, 5H), 7.11–7.04 (m, 1H), 6.99–6.91 (m, 4H), 4.18–4.08 (m, 1H), 4.02 (m, 1H), 3.91 (m, 1H), 3.82–3.72 (m, 1H), 2.54 (br s, 2H), 2.36 (br s, 3H), 2.25–2.13 (m, 1H), 1.90–1.80 (m, 1H), 1.76 (m, 1H), 1.62 (m, 1H), 1.51 (m, 2H), 1.47–1.32 (m, 4H), 1.30–1.01 (m, 5H) ppm. ^13^C{^1^H} NMR (101 MHz, CDCl_3_) δ 183.2, 178.3, 162.08 (d, ^1^*J*_C–F_ = 247.2 Hz), 162.05 (d, ^1^*J*_C–F_’ = 247.2 Hz), 142.0, 137.4 (d, ^4^*J*_C–F_ = 3.3 Hz), 137.2 (d, ^4^*J*_C–F_’ = 3.3 Hz), 132.4, 130.1 (2C, d, ^3^*J*_C–F_ = 8.2 Hz), 130.0 (2C, d, ^3^*J*_C–F_’ = 8.2 Hz), 128.9, 125.9, 123.2, 115.45 (2C, d, ^2^*J*_C–F_ = 21.5 Hz), 115.40 (2C, d, ^2^*J*_C–F_’ = 21.5 Hz), 109.6, 68.4, 65.8, 61.1, 55.0, 49.7, 42.9, 39.5, 32.9, 25.9, 25.0, 24.3, 24.1, 23.6, 15.3 ppm. ^19^F NMR (376 MHz, CDCl_3_) δ −114.8 ppm. IR (ATR): 3292, 3058, 2934, 2858, 1705, 1607, 1541, 1504, 1490, 1464, 1355, 1227, 1158, 1103, 946, 829, 745 cm^–1^. HRMS (ESI-QTOF) *m*/*z*: [M + H]^+^ Calcd for C_34_H_39_F_2_N_4_OS 589.2807; Found 589.2810.

#### 1-(2-(3,3-Dimethyl-2-oxoindolin-1-yl)ethyl)-3-((*S*)-(6-methoxyquinolin-4-yl)((1*S*,2*S*,4*S*,5*R*)-5-vinylquinuclidin-2-yl)methyl)thiourea (**C5**)

Catalyst **C5** was prepared according to the general procedure using **8** (112 mg, 0.55 mmol) and QN-NCS (183 mg, 0.5 mmol) as a white solid (205 mg, 0.036 mmol, 72% yield). Mp 130–135 °C.  = −85.3 [(*c* = 1.0, CHCl_3_)]. ^1^H NMR (500 MHz, CDCl_3_) δ 8.71 (d, *J* = 4.5 Hz, 1H), 7.98 (d, *J* = 9.2 Hz, 1H), 7.60 (m, 2H), 7.36 (dd, *J* = 9.2, 2.6 Hz, 1H), 7.10 (d, *J* = 7.0 Hz, 1H), 6.96 (m, 3H), 5.70 (m, 1H), 5.14–5.03 (m, 2H), 4.12 (m, 5H), 4.00 (s, 3H), 3.89–3.60 (m, 3H), 3.46 (s, 1H), 3.23 (m, 1H), 3.07 (m, 1H), 2.64–2.50 (m, 1H), 1.85 (m, 3H), 1.68–1.52 (m, 1H), 1.30 (s, 3H), 1.26 (s, 3H), 1.23 (m, 1H), 1.01 (m, 1H) ppm. ^13^C{^1^H} NMR (126 MHz, CDCl_3_) δ 183.1, 182.5, 158.3, 147.8, 147.5, 144.8, 142.7, 141.4, 137.9, 135.5, 131.6, 128.0, 127.7, 122.8, 122.4, 122.3, 116.8, 108.6, 102.5, 60.5, 56.1, 54.4, 44.3, 42.6, 42.2, 39.2, 37.6, 29.7, 26.9, 25.4, 24.6, 24.5, 24.4 ppm. IR (ATR): 3253, 3059, 2934, 2868, 1692, 1608, 1545, 1508, 1487,1469, 1461, 1431, 1384, 1359, 1300, 1025, 915, 857, 831, 721 cm^–1^. HRMS (ESI-QTOF) *m*/*z*: [M + H]^+^ Calcd for C_33_H_40_N_5_O_2_S 570.2897; Found 570.2894.

#### 3-((2-(3,3-Bis(4-fluorophenyl)-2-oxoindolin-1-yl)ethyl)amino)-4-(((*S*)-(6-methoxyquinolin-4-yl)((1*S*,2*S*,4*S*,5*R*)-5-vinylquinuclidin-2-yl)methyl)amino)cyclobut-3-ene-1,2-dione (**C6**)

The *N*-aminoalkyl isatin derivative **4** (0.55 mmol, 1.1 equiv) was dissolved in MeOH (10 mL), and then QNA-semisquarate (224 mg, 0.50 mmol, 1.0 equiv) was added. The reaction was stirred until the starting products were consumed. The solid was filtered and washed with cold MeOH to afford pure catalyst **C6** as a white solid (210 mg, 0.275 mmol, 55% yield). Mp 287–293 °C.  = −27.0 [(*c* = 0.50, CHCl_3_)]. ^1^H NMR (400 MHz, DMSO-*d*_6_) δ 8.76 (d, *J* = 4.5 Hz, 1H), 8.07–7.97 (m, 1H), 7.95 (d, *J* = 9.2 Hz, 1H), 7.76 (br s, 1H), 7.57 (d, *J* = 4.6 Hz, 1H), 7.41 (dd, *J* = 9.2, 2.5 Hz, 1H), 7.27 (d, *J* = 7.5 Hz, 1H), 7.19 (m, 2H), 7.10–7.02 (m, 5H), 7.02–6.91 (m, 3H), 5.93 (m, 1H), 4.98 (m, 2H), 3.97 (m, 1H), 3.89 (m, 4H), 3.81 (m, 2H), 3.33 (m, 3H) (under the DMSO signal), 3.22–3.08 (m, 2H), 2.71–2.53 (m, 2H), 2.25 (m, 1H), 1.48 (m, 5H), 0.57 (br s, 1H) ppm. ^13^C{^1^H} NMR (101 MHz, DMSO-*d*_6_) δ 183.1, 182.1, 176.8, 168.4, 167.3, 161.69 (d, ^1^*J*_C–F_ = 247.0 Hz), 161.67 (d, ^1^*J*_C–F_’ = 247.0 Hz), 158.3, 148.1, 144.7, 144.2, 142.7, 141.9, 138.10 (d, ^4^*J*_C–F_ = 3.1 Hz), 138.08 (d, ^4^*J*_C–F_’ = 3.1 Hz) 132.2, 131.9, 130.4 (d, ^3^*J*_C–F_ = 8.2 Hz, 2C), 130.3 (d, ^3^*J*_C–F_’ = 8.2 Hz, 2C) 128.9, 127.9, 126.0, 123.3, 122.4, 119.9, 115.7 (d, ^2^*J*_C–F_ = 21.6 Hz, 2C), 115.6 (d, ^2^*J*_C–F_’ = 21.6 Hz, 2C) 114.7, 110.2, 101.9, 60.9, 59.4, 56.1, 41.4, 41.0, 40.7, 40.5, 40.4, 40.2, 40.0, 27.8, 26.7 ppm. ^19^F NMR (470 MHz, DMSO-*d*_6_) δ −115.1 ppm. IR (ATR): 3304, 2941, 1798, 1707, 1651, 1578, 1542, 1505, 1348, 1227, 1157, 1098, 824, 761, 747 cm^–1^. HRMS (ESI-QTOF) *m*/*z*: [M + H]^+^ Calcd for C_46_H_42_F_2_N_5_O_4_ 766.3199; Found 766.3206.

### General Procedure for the Enantioselective α-Amination of 4-Substituted Pyrazolones with Azodicarboxylates Using Homogeneous Catalysts

To a solution of 4-substituted pyrazolone **9a**–**9o** (0.1 mmol) and catalyst **C1** (0.005–0.01 mmol, 0.05–0.1 equiv) in toluene (1 mL), azodicarboxylates **10**–**12** (0.12 mmol, 1.2 equiv) were added at room temperature. The mixture was stirred in a Wheaton vial until the starting materials were consumed (monitored by ^1^H NMR). After the completion of the reaction, the solvent was removed under reduced pressure. The crude reaction mixture was purified by flash column chromatography (hexane/EtOAc mixtures) to afford the corresponding pure α-aminated products **13a**, **14a**,and **15a**–**15o**. The enantiomeric ratio was determined by chiral-phase HPLC analysis using mixtures of hexane/2-propanol as an eluent.

#### Dibenzyl (*R*)-1-(4-Benzyl-3-methyl-5-oxo-1-phenyl-4,5-dihydro-1*H*-pyrazol-4-yl)hydrazine-1,2-dicarboxylate (**13a**)^8a^

Compound **13a** was obtained according to the general procedure using pyrazolone **9a** (26 mg, 0.1 mmol), catalyst **C1** (7 mg, 0.01 mmol), and dibenzyl azodicarboxylate (35 mg, 0.12 mmol). Purification by flash chromatography (hexane/EtOAc: 4/1) afforded the pure product as a brown solid (50 mg, 0.089 mmol, 89%).  = −68.1 (*c* = 1, CHCl_3_). [Lit.^8a^ = −73.4 (*c* = 0.31, CHCl_3_, 84% ee for (*R*) enantiomer)]. ^1^H NMR (500 MHz, CDCl_3_) δ 7.42–7.31 (m, 7H), 7.29–7.20 (m, 7H), 7.15–7.05 (m, 6H), 6.96 (br s, 1H), 5.35–5.05 (m, 4H), 3.38 (d, *J* = 12.5 Hz, 1H), 3.10 (d, *J* = 12.5 Hz, 1H), 2.39 (s, 3H) ppm. HPLC: Lux i-Cellulose-5 column, hexane//i-PrOH 90:10, 0.5 mL/min, λ = 240 nm. Minor enantiomer (*S*): t_R_ = 30.78 min, major enantiomer (*R*): t_R_ = 37.18 min, (84:16 er).

#### Diisopropyl (*R*)-1-(4-Benzyl-3-methyl-5-oxo-1-phenyl-4,5-dihydro-1*H*-pyrazol-4-yl)hydrazine-1,2-dicarboxylate (**14a**)^8a^

Compound **14a** was obtained according to the general procedure using pyrazolone **9a** (26 mg, 0.1 mmol), catalyst **C1** (7 mg, 0.01 mmol), and diisopropyl azodicarboxylate (24 mg, 0.12 mmol). Purification by flash chromatography (hexane/EtOAc: 4/1) afforded the pure product as a yellow oil (39 mg, 0.084 mmol, 84%).  = −95.8 (*c* = 0.6, CHCl_3_). [Lit.^8a^ = −73.4 (*c* = 0.98, CHCl_3_, 84% ee for (*R*) enantiomer)]. ^1^H NMR (500 MHz, CDCl_3_) δ 7.46–7.41 (m, 2H), 7.26 (t, *J* = 8.0 Hz, 2H), 7.17–7.05 (m, 6H), 5.04 (m, 1H), 4.90 (m, 1H), 3.34 (d, *J* = 12.6 Hz, 1H), 3.07 (d, *J* = 12.6 Hz, 1H), 2.38 (s, 3H), 1.36–1.31 (m, 6H), 1.17–1.11 (m, 6H) ppm. HPLC: Lux i-Cellulose-5 column, hexane//i-PrOH 95:5, 1.0 mL/min, λ = 254 nm. Major enantiomer (*R*): *t*_*R*_ = 9.06 min, minor enantiomer (*S*): *t*_*R*_ = 17.17 min, (93:7 er).

#### Di-*tert-*butyl (*R*)-1-(4-Benzyl-3-methyl-5-oxo-1-phenyl-4,5-dihydro-1*H*-pyrazol-4-yl)hydrazine-1,2-dicarboxylate (**15a**)^8a^

Compound **15a** was obtained according to the general procedure using pyrazolone **9a** (26 mg, 0.1 mmol), catalyst **C1** (7 mg, 0.01 mmol), and di-*tert*-butyl azodicarboxylate (28 mg, 0.12 mmol). Purification by flash chromatography (hexane/EtOAc: 8/1) afforded the pure product as a yellow oil (42 mg, 0.085 mmol, 85%).  = −100.4 (*c* = 0.44, CHCl_3_). [Lit.^8a^ = −67.7 (*c* = 0.31, CHCl_3_, 82% ee for (*R*) enantiomer)]. ^1^H NMR (400 MHz, CDCl_3_) δ 7.45–7.39 (m, 2H), 7.25 (t, *J* = 8.0 Hz, 2H), 7.15–7.05 (m, 5H), 6.74 (br s, 1H), 3.34 (d, *J* = 12.5 Hz, 1H), 3.03 (d, *J* = 12.6 Hz, 1H), 2.37 (s, 3H), 1.54–1.47 (m, 9H), 1.35–1.26 (m, 9H) ppm. HPLC: Lux i-Cellulose-5 column, Hexane/i-PrOH 93:7, 1.0 mL/min, λ = 254 nm. Major enantiomer (*R*): *t*_*R*_ = 6.27 min, minor enantiomer (*S*): *t*_*R*_ = 15.74 min, (95:5 er).

#### Di-*tert*-butyl (*R*)-1-(4-Benzyl-3-ethyl-5-oxo-1-phenyl-4,5-dihydro-1*H*-pyrazol-4-yl)hydrazine-1,2-dicarboxylate (**15b**)

Compound **15b** was obtained according to the general procedure using pyrazolone **9b** (28 mg, 0.1 mmol), catalyst **C1** (3.5 mg, 0.005 mmol), and di-*tert*-butyl azodicarboxylate (28 mg, 0.12 mmol). Purification by flash chromatography (hexane/EtOAc: 8/1) afforded the pure product as a yellow oil (39 mg, 0.077 mmol, 77%).  = −95.8 (*c* = 0.6, CHCl_3_). ^1^H NMR (400 MHz, CDCl_3_) δ 7.50–7.43 (m, 2H), 7.30–7.20 (m, 2H), 7.08 (m, 6H), 6.80 (br s, 1H), 3.33 (d, *J* = 12.5 Hz, 1H), 3.03 (d, *J* = 12.5 Hz, 1H), 2.87 (m, 1H), 2.70 (m, 1H), 1.68–1.45 (m, 9H), 1.43–1.34 (m, 9H), 1.29 (t, *J* = 7.3 Hz, 3H) ppm. ^13^C{^1^H} NMR (101 MHz, CDCl_3_) δ 172.5, 163.8, 156.1, 153.0, 137.7, 131.5, 129.7 (2C), 128.4 (2C), 128.0 (2C), 127.6 (2C), 124.8, 119.1, 81.7, 73.8, 39.5, 28.23 (3C), 28.16 (3C), 27.9, 21.6, 8.8 ppm. IR (ATR): 3286, 2978, 2934, 1702, 1598, 1502, 1455, 1392, 1366, 1325, 1245, 1147, 904, 756, 724, 700, 692 cm^–1^. HRMS (ESI-QTOF) *m*/*z*: [M + Na]^+^ Calcd for C_28_H_36_N_4_NaO_5_ 531.2578; Found 531.2579. HPLC: Lux i-Cellulose-5 column, hexane/i-PrOH 93:7, 1.0 mL/min, λ = 254 nm. Major enantiomer (*R*): *t*_*R*_ = 5.97 min, minor enantiomer (*S*): *t*_*R*_ = 15.77 min, (96:4 er).

#### Di-*tert*-butyl (*R*)-1-(4-Benzyl-3-isopropyl-5-oxo-1-phenyl-4,5-dihydro-1*H*-pyrazol-4-yl)hydrazine-1,2-dicarboxylate (**15c**)

Compound **15c** was obtained according to the general procedure using pyrazolone **9c** (29 mg, 0.1 mmol), catalyst **C1** (7 mg, 0.01 mmol), and di-*tert*-butyl azodicarboxylate (28 mg, 0.12 mmol). Purification by flash chromatography (hexane/EtOAc: 8/1) afforded the pure product as a yellow oil (39 mg, 0.075 mmol, 75%).  = −102.1 (*c* = 0.62, CHCl_3_). ^1^H NMR (400 MHz, CDCl_3_) δ 7.51 (d, *J* = 7.9 Hz, 2H), 7.27 (t, *J* = 8.0 Hz, 2H), 7.17–7.01 (m, 6H), 6.80 (br s, 1H), 3.36 (d, *J* = 12.0 Hz, 1H), 3.11 (d, *J* = 12.4 Hz, 1H), 1.73–1.46 (m, 9H), 1.46–1.18 (m, 16H) ppm. ^13^C{^1^H} NMR (101 MHz, CDCl_3_) δ 172.8, 166.2, 156.0, 153.5, 137.7, 131.6, 130.1, 130.0 (2C), 128.5 (2C), 128.4 (2C), 127.9, 127.7, 124.8, 118.6, 81.5, 40.0, 28.6, 28.2 (3C), 27.9 (3C), 22.4, 19.5 (2C) ppm. IR (ATR): 3289, 2978, 2934, 1704, 1597, 1500, 1457, 1367, 1324, 1245, 1148, 1070, 906, 755, 725, 690 cm^–1^. HRMS (ESI-QTOF) *m*/*z*: [M + Na]^+^ Calcd for C_29_H_38_N_4_NaO_5_ 545.2734; Found 545.2729. HPLC: Lux i-Cellulose-5 column, hexane/i-PrOH 93:7, 1.0 mL/min, λ = 254 nm. Major enantiomer (*R*): *t*_*R*_ = 4.92 min, minor enantiomer (*S*): *t*_*R*_ = 10.27 min, (97:3 er).

#### Di-*tert*-butyl (*R*)-1-(4-Benzyl-5-oxo-1,3-diphenyl-4,5-dihydro-1*H*-pyrazol-4-yl)hydrazine-1,2-dicarboxylate (**15d**)

Compound **15d** was obtained according to the general procedure using pyrazolone **9d** (33 mg, 0.1 mmol), catalyst **C1** (7 mg, 0.01 mmol), and di-*tert*-butyl azodicarboxylate (28 mg, 0.12 mmol). Purification by flash chromatography (hexane/EtOAc: 8/1) afforded the pure product as a yellow oil (46 mg, 0.082 mmol, 82%).  = +7.6 (*c* = 0.7, CHCl_3_). ^1^H NMR (400 MHz, CDCl_3_) δ 8.55 (m, 2H), 7.67–7.42 (m, 5H), 7.38–7.23 (m, 2H), 7.15 (t, *J* = 7.4 Hz, 1H), 7.08 (t, *J* = 7.4 Hz, 1H), 7.00 (t, *J* = 7.4 Hz, 2H), 6.95 (m, 1H), 6.81 (d, *J* = 7.1 Hz, 2H), 3.70 (d, *J* = 12.2 Hz, 1H), 3.43 (d, *J* = 12.4 Hz, 1H), 1.77–1.42 (m, 9H), 1.36–1.07(m, 9H) ppm. ^13^C{^1^H} NMR (101 MHz, CDCl_3_) δ 173.2, 157.1, 156.1, 154.2, 137.3, 131.5, 130.4, 130.1, 130.0 (2C), 128.9, 128.6 (2C), 128.5, 127.83, 127.78, 127.6 (2C), 126.8, 126.1, 125.4, 119.4, 84.5, 81.6, 73.1, 40.4, 28.3 (3C), 27.7 (3C) ppm. IR (ATR): 3286, 2978, 2934, 1706, 1596, 1494, 1451, 1392, 1367, 1327, 1243, 1147, 908, 761, 725, 690 cm^–1^. HRMS (ESI-QTOF) *m*/*z*: [M + Na]^+^ Calcd for C_32_H_36_N_4_NaO_5_ 579.2578; Found 579.2599. HPLC: Lux i-Cellulose-5 column, hexane/i-PrOH 95:5, 1.0 mL/min, λ = 254 nm. Major enantiomer (*R*): *t*_*R*_ = 5.00 min, minor enantiomer (*S*): *t*_*R*_ = 7.30 min, (95:5 er).

#### Di-*tert*-butyl (*R*)-1-(3-Methyl-4-(4-methylbenzyl)-5-oxo-1-phenyl-4,5-dihydro-1*H*-pyrazol-4-yl)hydrazine-1,2-dicarboxylate (**15e**)

Compound **15e** was obtained according to the general procedure using pyrazolone **9e** (28 mg, 0.1 mmol), catalyst **C1** (7 mg, 0.01 mmol), and di-*tert*-butyl azodicarboxylate (28 mg, 0.12 mmol). Purification by flash chromatography (hexane/EtOAc: 8/1) afforded the pure product as a yellow oil (42 mg, 0.083 mmol, 83%).  = −98.9 (*c* = 0.8, CHCl_3_). ^1^H NMR (400 MHz, CDCl_3_) δ 7.44 (d, *J* = 7.2 Hz, 2H), 7.30–7.20 (m, 2H), 7.09 (t, *J* = 7.3 Hz, 1H), 6.99–6.87 (m, 4H), 6.77 (br s, 1H), 3.30 (d, *J* = 12.6 Hz, 1H), 3.00 (d, *J* = 12.6 Hz, 1H), 2.36 (s, 3H), 2.18 (s, 3H), 1.68–1.46 (m, 9H), 1.45–1.22 (m, 9H) ppm. ^13^C{^1^H} NMR (101 MHz, CDCl_3_) δ 172.3, 160.3, 156.1, 153.0, 137.4, 137.3, 129.6 (2C), 128.7 (2C), 128.4, 128.2 (2C), 124.9 (2C), 119.2, 83.8, 81.7, 73.6, 38.7, 28.2 (3C), 28.0 (3C), 21.0, 14.4 ppm. IR (ATR): 3286, 2978, 2930, 1704, 1598, 1502, 1367, 1269, 1247, 1149, 878, 757, 731, 690 cm^–1^. HRMS (ESI-QTOF) *m*/*z*: [M + Na]^+^ Calcd for C_28_H_36_N_4_NaO_5_ 531.2578; Found 531.2585. HPLC: Lux i-Cellulose-5 column, hexane/i-PrOH 93:7, 1.0 mL/min, λ = 254 nm. Major enantiomer (*R*): *t*_*R*_ = 6.66 min, minor enantiomer (*S*): *t*_*R*_ = 17.37 min, (95:5 er).

#### Di-*tert*-butyl (*R*)-1-(4-(4-Methoxybenzyl)-3-methyl-5-oxo-1-phenyl-4,5-dihydro-1*H*-pyrazol-4-yl)hydrazine-1,2-dicarboxylate (**15f**)

Compound **15f** was obtained according to the general procedure using pyrazolone **9f** (29 mg, 0.1 mmol), catalyst **C1** (7 mg, 0.01 mmol), and di-*tert*-butyl azodicarboxylate (28 mg, 0.12 mmol). Purification by flash chromatography (hexane/EtOAc: 8/1) afforded the pure product as a yellow oil (40 mg, 0.077 mmol, 77%).  = −106.1 (*c* = 0.72, CHCl_3_). ^1^H NMR (400 MHz, CDCl_3_) δ 7.48–7.40 (m, 2H), 7.25 (t, *J* = 8.0 Hz, 2H), 7.11–7.05 (m, 1H), 6.99 (d, *J* = 8.6 Hz, 2H), 6.80 (br s, 1H), 6.64 (d, *J* = 8.7 Hz, 2H), 3.65 (s, 3H), 3.28 (d, *J* = 12.8 Hz, 1H), 2.98 (d, *J* = 12.8 Hz, 1H), 2.36 (s, 3H), 1.61–1.41 (m, 9H), 1.41–1.19 (m, 9H) ppm. ^13^C{^1^H} NMR (101 MHz, CDCl_3_) δ 172.3, 159.1, 156.1, 153.0, 137.4, 130.8 (2C), 128.4 (2C), 124.9, 123.2, 119.2, 113.5 (2C), 81.7, 73.6, 67.1, 55.1, 38.3, 31.6, 28.2 (3C), 28.0 (3C), 22.6, 14.4 ppm. IR (ATR): 3285, 2978, 2930, 1707, 1600, 1501, 1370, 1249, 1146, 1032, 754, 732, 688 cm^–1^. HRMS (ESI-QTOF) *m*/*z*: [M + Na]^+^ Calcd for C_28_H_36_N4NaO_6_ 547.2527; Found 547.2511. HPLC: Lux i-Cellulose-5 column, hexane/i-PrOH 93:7, 1.0 mL/min, λ = 254 nm. Major enantiomer (*R*): *t*_*R*_ = 9.69 min, minor enantiomer (*S*): *t*_*R*_: 24.39 min, (96:4 er).

#### Di-*tert*-butyl (*R*)-1-(4-(4-Bromobenzyl)-3-methyl-5-oxo-1-phenyl-4,5-dihydro-1*H*-pyrazol-4-yl)hydrazine-1,2-dicarboxylate (**15g**)

Compound **15g** was obtained according to the general procedure using pyrazolone **9g** (34 mg, 0.1 mmol), catalyst **C1** (7 mg, 0.01 mmol), and di-*tert*-butyl azodicarboxylate (28 mg, 0.12 mmol). Purification by flash chromatography (hexane/EtOAc: 8/1) afforded the pure product as a yellow oil (41 mg, 0.071 mmol, 71%).  = −97.7 (*c* = 0.52, CHCl_3_). ^1^H NMR (400 MHz, CDCl_3_) δ 7.47–7.38 (m, 2H), 7.32–7.21 (m, 4H), 7.11 (t, *J* = 7.4 Hz, 1H), 6.94 (d, *J* = 8.3 Hz, 2H), 6.79 (br s, 1H), 3.28 (d, *J* = 12.6 Hz, 1H), 2.98 (d, *J* = 12.7 Hz, 1H), 2.35 (s, 3H), 1.59–1.45 (m, 9H), 1.40–1.24 (m, 9H) ppm. ^13^C{^1^H} NMR (101 MHz, CDCl_3_) δ 172.0, 160.1, 156.1, 152.9, 137.2, 131.4 (2C), 131.2 (2C), 130.5, 128.6 (2C), 125.2, 121.9, 119.2, 81.9, 73.4, 38.4, 29.7, 28.2 (3C), 28.0 (3C), 14.4 ppm. IR (ATR): 3282, 2981, 2927, 2850, 1703, 1597, 1501, 1490, 1366, 1245, 1150, 1010, 758, 736 cm^–1^. HRMS (ESI-QTOF) *m*/*z*: [M + Na]^+^ Calcd for C_27_H_33_BrN_4_NaO_5_ 595.1527; Found 595.1543. HPLC: Lux i-Cellulose-5 column, hexane/i-PrOH 95:5, 1.0 mL/min, λ = 254 nm. Major enantiomer (*R*): *t*_*R*_ = 6.45 min, minor enantiomer (*S*): *t*_*R*_ = 10.75 min, (93:7 er).

#### Di-*tert*-butyl (*R*)-1-(3-Methyl-5-oxo-1-phenyl-4-(4-(trifluoromethyl)benzyl)-4,5-dihydro-1*H*-pyrazol-4-yl)hydrazine-1,2-dicarboxylate (**15h**)

Compound **15h** was obtained according to the general procedure using pyrazolone **9h** (33 mg, 0.1 mmol), catalyst **C1** (3.5 mg, 0.005 mmol), and di-*tert*-butyl azodicarboxylate (28 mg, 0.12 mmol). Purification by flash chromatography (hexane/EtOAc: 8/1) afforded the pure product as a yellow oil (41 mg, 0.072 mmol, 72%).  = −64.8 (*c* = 0.99, CHCl_3_). ^1^H NMR (400 MHz, CDCl_3_) δ 7.41–7.31 (m, 4H), 7.29–7.16 (m, 4H), 7.09 (t, *J* = 7.3 Hz, 1H), 6.86 (br s, 1H), 3.38 (d, *J* = 12.5 Hz, 1H), 3.07 (d, *J* = 12.5 Hz, 1H), 2.38 (s, 3H), 1.68–1.45 (m, 9H), 1.43–1.28 (m, 9H) ppm. ^13^C{^1^H} NMR (101 MHz, CDCl_3_) δ 171.8, 160.1, 156.2, 152.9, 137.1, 135.8, 130.5–129.5 (q, ^2^*J*_C–F_ = 32.5 Hz, 2C) 130.2, 128.5 (2C), 127.9–119.8 (q, ^1^*J*_C–F_ = 272.2 Hz), 125.3, 125.0–124.9 (q, ^3^*J*_C–F_ = 3.7 Hz, 2C), 122.5, 119.2, 81.9, 77.2, 73.5, 38.6, 28.2 (3C), 27.9 (3C), 14.4 ppm. ^19^F NMR (470 MHz, CDCl_3_) δ −62.8 ppm. IR (ATR): 3282, 2978, 2934, 1704, 1598, 1502, 1369, 1325, 1247, 1149, 1125, 1110, 1068, 757, 731, 692 cm^–1^. HRMS (ESI-QTOF) *m*/*z*: [M + Na]^+^ Calcd for C_28_H_33_F_3_N_4_NaO_5_ 585.2295; Found 585.2280. HPLC: Lux i-Cellulose-5 column, hexane/i-PrOH 97:3, 1.0 mL/min, λ = 254 nm. Major enantiomer (*R*): *t*_*R*_ = 4.62 min, minor enantiomer (*S*): *t*_*R*_ = 6.72 min, (92:8 er).

#### Di-*tert*-butyl (*R*)-1-(3-Methyl-4-(4-nitrobenzyl)-5-oxo-1-phenyl-4,5-dihydro-1*H*-pyrazol-4-yl)hydrazine-1,2-dicarboxylate (**15i**)

Compound **15i** was obtained according to the general procedure using pyrazolone **9i** (31 mg.0.1 mmol), catalyst **C1** (3.5 mg, 0.005 mmol), and di-*tert*-butyl azodicarboxylate (28 mg, 0.12 mmol). Purification by flash chromatography (hexane/EtOAc: 4/1) afforded the pure product as a yellow oil (41 mg, 0.076 mmol, 76%).  = −127.5 (*c* = 0.12, CHCl_3_). ^1^H NMR (400 MHz, CDCl_3_) δ 7.98 (d, *J* = 8.7 Hz, 2H), 7.43 (m, 2H), 7.25 (m, 4H), 7.09 (t, *J* = 7.4 Hz, 1H), 6.99 (br s, 1H), 3.43 (d, *J* = 12.5 Hz, 1H), 3.12 (d, *J* = 12.5 Hz, 1H), 2.39 (s, 3H), 1.64–1.42 (m, 9H), 1.46–1.22 (m, 9H) ppm. ^13^C{^1^H} NMR (101 MHz, CDCl_3_) δ 171.7, 160.0, 156.2, 155.8, 152.8, 147.5, 139.4, 137.0, 130.8 (2C), 128.7 (2C), 125.3, 123.1 (2C), 118.7, 84.2, 82.0, 73.3, 38.5, 28.2 (3C), 27.9 (3C), 14.5 ppm. IR (ATR): 3286, 2981, 2930, 1704, 1598, 1523, 1502, 1369, 1347, 1268, 1247, 1147, 853, 757, 727 cm^–1^. HRMS (ESI-QTOF) *m*/*z*: [M + Na]^+^ Calcd for C_27_H_33_N_5_NaO_7_ 562.2272; Found 562.2262. HPLC: Lux i-Cellulose-5 column, hexane/i-PrOH 93:7, 1.0 mL/min, λ = 254 nm. Major enantiomer (*R*): *t*_*R*_ = 14.36 min, minor enantiomer (*S*): *t*_*R*_ = 20.47 min, (92:8 er).

#### Di-*tert*-butyl (*R*)-1-(3-Methyl-4-(2-nitrobenzyl)-5-oxo-1-phenyl-4,5-dihydro-1*H*-pyrazol-4-yl)hydrazine-1,2-dicarboxylate (**15j**)

Compound **15j** was obtained according to the general procedure using pyrazolone **9j** (31 mg, 0.1 mmol), catalyst **C1** (7 mg, 0.01 mmol), and di-*tert*-butyl azodicarboxylate (28 mg, 0.12 mmol). Purification by flash chromatography (hexane/EtOAc: 4/1) afforded the pure product as a yellow oil (49 mg, 0.091 mmol, 91%).  = −191.9 (*c* = 0.84, CHCl_3_). ^1^H NMR (400 MHz, CDCl_3_) δ 7.74 (d, *J* = 7.6 Hz, 1H), 7.50–7.40 (m, 2H), 7.32–7.18 (m, 5H), 7.08 (d, *J* = 7.3 Hz, 1H), 6.89 (br s, 1H), 3.87 (d, *J* = 12.7 Hz, 1H), 3.67 (d, *J* = 13.1 Hz, 1H), 2.27 (s, 3H), 1.68–1.43 (m, 9H), 1.45–1.17 (m, 9H) ppm. ^13^C{^1^H} NMR (101 MHz, CDCl_3_) δ 171.9, 160.8, 155.7, 152.8, 150.3, 137.2, 133.4 (2C), 132.1, 128.8, 128.5 (2C), 126.2, 125.0, 124.8, 118.6, 81.9, 73.5, 34.0, 31.6, 28.2 (3C), 27.9 (3C), 22.6 ppm. IR (ATR): 3288, 2978, 2931, 1705, 1529, 1500, 1369, 1270, 1248, 1149, 758, 737, 725 cm^–1^. HRMS (ESI-QTOF) *m***/***z***:** [M + Na]^+^ Calcd for C_27_H_33_N_5_NaO_7_ 562.2272; Found 562.2273. HPLC: Lux i-Cellulose-5 column, hexane/i-PrOH 90:10, 1.0 mL/min, λ = 254 nm. Major enantiomer (*R*): *t*_*R*_ = 8.00 min, minor enantiomer (*S*): *t*_*R*_ = 12.57 min, (90:10 er).

#### Di-*tert*-butyl (*R*)-1-(4-(2,6-Dichlorobenzyl)-3-methyl-5-oxo-1-phenyl-4,5-dihydro-1*H*-pyrazol-4-yl)hydrazine-1,2-dicarboxylate (**15k**)

Compound **15k** was obtained according to the general procedure using pyrazolone **9k** (33 mg, 0.1 mmol), catalyst **C1** (7 mg, 0.01 mmol), and di-*tert*-butyl azodicarboxylate (28 mg, 0.12 mmol). Purification by flash chromatography (hexane/EtOAc: 8/1) afforded the pure product as a yellow oil (32 mg, 0.057 mmol, 57%).  = −113.7 (*c* = 0.7, CHCl_3_). ^1^H NMR (400 MHz, CDCl_3_) δ 7.56 (d, *J* = 8.3 Hz, 2H), 7.26 (t, *J* = 7.8 Hz, 2H), 7.18 (d, *J* = 8.0 Hz, 2H), 7.08 (t, *J* = 7.3 Hz, 1H), 7.00 (t, *J* = 8.0 Hz, 1H), 6.93 (br s, 1H), 3.90 (d, *J* = 13.8 Hz, 1H), 3.72 (d, *J* = 15.1 Hz, 1H), 2.37 (s, 3H), 1.69–1.43 (m, 9H), 1.42–1.24 (m, 9H) ppm. ^13^C{^1^H} NMR (101 MHz, CDCl_3_) δ 172.4, 161.6, 155.9, 153.0, 137.8 (2C), 136.8, 130.1, 129.3, 128.5 (4C), 124.6, 118.7 (2C), 83.9, 81.8, 72.8, 33.6, 28.2 (3C), 27.9 (3C), 15.7 ppm. IR (ATR): 3289, 2981, 2934, 1706, 1596, 1500, 1367, 1245, 1147, 780, 755, 735 cm^–1^. HRMS (ESI-QTOF) *m*/*z*: [M + Na]^+^ Calcd for C_27_H_32_Cl_2_N_4_NaO_5_ 585.1642; Found 585.1655. HPLC: Lux i-Cellulose-5 column, hexane/i-PrOH 95:5, 1.0 mL/min, λ = 254 nm. Major enantiomer (*R*): *t*_*R*_ = 8.52 min, Minor enantiomer (*S*): *t*_*R*_ = 14.11 min, (70:30 er).

#### Di-*tert*-butyl (*R*)-1-(4-Benzyl-1-(4-chlorophenyl)-3-methyl-5-oxo-4,5-dihydro-1*H*-pyrazol-4-yl)hydrazine-1,2-dicarboxylate (**15l**)

Compound **15l** was obtained according to the general procedure using pyrazolone **9l** (30 mg, 0.1 mmol), catalyst **C1** (7.3 mg, 0.01 mmol), and di-*tert*-butyl azodicarboxylate (28 mg, 0.12 mmol). Purification by flash chromatography (hexane/EtOAc: 8/1) afforded the pure product as a yellow oil (48 mg, 0.090 mmol, 90%).  = −113.7 (*c* = 0.84, CHCl_3_). ^1^H NMR (400 MHz, CDCl_3_) δ 7.51–7.36 (m, 2H), 7.28–7.14 (m, 3H), 7.14–7.07 (m, 2H), 7.06 (m, 2H), 6.85 (br s, 1H), 3.33 (d, *J* = 12.6 Hz, 1H), 3.03 (d, *J* = 12.5 Hz, 1H), 2.37 (s, 3H), 1.68–1.46 (m, 9H), 1.45–1.24 (m, 9H) ppm. ^13^C{^1^H} NMR (101 MHz, CDCl_3_) δ 172.1, 160.6, 156.0, 153.0, 137.8, 136.0, 131.3, 129.7 (2C), 129.0, 128.5 (2C), 128.2, 128.1 (2C), 127.8, 125.3, 120.0, 81.9, 39.0, 28.2 (3C), 28.0 (3C), 14.4 ppm. IR (ATR): 3278, 2978, 2927, 1706, 1593, 1494, 1370, 1267, 1245, 1150 1010, 973, 827, 699 cm^–1^. HRMS (ESI-QTOF) *m*/*z*: [M + Na]^+^ Calcd for C_27_H_33_ClN_4_NaO_5_ 551.2032; Found 551.2041. HPLC: Lux i-Cellulose-5 column, hexane/i-PrOH 93:7, 1.0 mL/min, λ = 254 nm. Major enantiomer (*R*): *t*_*R*_ = 5.09 min, minor enantiomer (*S*): *t*_*R*_ = 8.40 min, (95:5 er).

#### Di-*tert*-butyl (*R*)-1-(4-Benzyl-1,3-dimethyl-5-oxo-4,5-dihydro-1*H*-pyrazol-4-yl)hydrazine-1,2-dicarboxylate (**15m**)

Compound **15m** was obtained according to the general procedure using pyrazolone **9m** (20 mg, 0.1 mmol), catalyst **C1** (7.3 mg, 0.01 mmol), and di-*tert*-butyl azodicarboxylate (28 mg, 0.12 mmol). Purification by flash chromatography (hexane/EtOAc: 4/1) afforded the pure product as a colorless oil (40 mg, 0.093 mmol, 93%).  = −16.2 (*c* = 0.64, CHCl_3_). ^1^H NMR (500 MHz, CDCl_3_) δ 7.26–7.21 (m, 3H), 7.07 (m, 2H), 6.73 (br s, 1H), 3.22 (d, *J* = 12.5 Hz, 1H), 2.94 (d, *J* = 12.5 Hz, 1H), 2.83 (s, 3H), 2.27 (s, 3H), 1.60–1.50 (m, 9H), 1.49–1.36 (m, 9H) ppm. ^13^C{^1^H} NMR (126 MHz, CDCl_3_) δ 173.0, 159.5, 153.0, 131.7, 129.8 (2C), 127.9 (2C), 127.7, 81.7, 72.5, 38.5, 31.6, 30.8, 28.2 (3C), 28.0 (3C), 22.6, 14.3 ppm. IR (ATR): 3263, 2986, 2931, 1698, 1457, 1366, 1329, 1267, 1245, 1150, 1121, 1048, 983, 895, 767, 727, 698 cm^–1^. HRMS (ESI-QTOF) *m*/*z*: [M + Na]^+^ Calcd for C_22_H_32_N_4_NaO_5_ 455.2265; Found. 455.2273. HPLC: Lux i-Amilose-1 column, hexane/i-PrOH 95:5, 1.0 mL/min, λ = 254 nm. Major enantiomer (*R*): *t*_*R*_ = 6.86 min, minor enantiomer (*S*): *t*_*R*_ = 11.57 min, (75:25 er).

#### Di-*tert*-butyl (*R*)-1-(4-Allyl-3-methyl-5-oxo-1-phenyl-4,5-dihydro-1*H*-pyrazol-4-yl)hydrazine-1,2-dicarboxylate (**15n**)

Compound **15n** was obtained according to the general procedure using pyrazolone **9n** (21 mg, 0.1 mmol), catalyst **C1** (7.3 mg, 0.01 mmol), and di-*tert*-butyl azodicarboxylate (28 mg, 0.12 mmol). Purification by flash chromatography (hexane/EtOAc: 8/1) afforded the pure product as a yellow oil (40 mg, 0.089 mmol, 89%).  = −34.4 (*c* = 0.64, CHCl_3_). ^1^H NMR (400 MHz, CDCl_3_) δ 7.86 (d, *J* = 7.9 Hz, 2H), 7.43–7.32 (m, 2H), 7.16 (d, *J* = 7.5 Hz, 1H), 6.71 (br s, 1H), 5.50–5.37 (m, 1H), 5.23–5.16 (m, 1H), 5.09 (d, *J* = 10.0 Hz, 1H), 2.74 (dd, *J* = 12.9, 7.3 Hz, 1H), 2.54 (d, *J* = 7.2 Hz, 1H), 2.27 (s, 3H), 1.59–1.45 (m, 9H), 1.39–1.24 (m, 9H) ppm. ^13^C{^1^H} NMR (101 MHz, CDCl_3_) δ 172.3, 160.6, 156.0, 153.0, 137.9, 129.0, 128.76 (2C), 124.9, 121.4, 118.7, 118.6, 81.7, 72.4, 40.6, 37.3, 28.2 (3C), 27.9 (3C), 13.9 ppm. IR (ATR): 3289, 2981, 2927, 1704, 1598, 1500, 1367, 1269, 1245, 1151, 753, 735, 690 cm^–1^. HRMS (ESI-QTOF) *m*/*z*: [M + Na]^+^ Calcd for C_23_H_32_N_4_NaO_5_ 467.2265; Found 467.2257. HPLC: Lux i-Cellulose-5 column, hexane/i-PrOH 93:7, 1.0 mL/min, λ = 254 nm. Major enantiomer (*R*): *t*_*R*_ = 5.31 min, minor enantiomer (*S*): *t*_*R*_: 6.98 min, (95:5 er).

#### Di-*tert*-butyl (*R*)-1-(4-(Ethoxycarbonyl)-3-methyl-5-oxo-1-phenyl-4,5-dihydro-1*H*-pyrazol-4-yl)hydrazine-1,2-dicarboxylate (**15o**)

Compound **15o** was obtained according to the general procedure using pyrazolone **9o** (26 mg, 0.1 mmol), catalyst **C1** (7.3 mg, 0.01 mmol), and di-*tert*-butyl azodicarboxylate (28 mg, 0.12 mmol). Purification by flash chromatography (hexane/EtOAc: 8/1) afforded the pure product as a yellow oil (47 mg, 0.096 mmol, 96%).  = −18.0 (*c* = 0.92, CHCl_3_). ^1^H NMR (400 MHz, CDCl_3_) δ 7.89 (d, *J* = 7.8 Hz, 2H), 7.42–7.32 (m, 2H), 7.15 (t, *J* = 7.4 Hz, 1H), 6.74 (br s, 1H), 4.08–3.90 (m, 2H), 3.05 (d, *J* = 13.3 Hz, 1H), 2.78 (d, *J* = 13.3 Hz, 1H), 2.31 (s, 3H), 1.55–1.45 (m, 9H), 1.41–1.27 (m, 9H), 1.03 (t, *J* = 7.1 Hz, 3H) ppm. ^13^C{^1^H} NMR (101 MHz, CDCl_3_) δ 171.1, 167.2, 159.9, 155.8, 152.6, 138.0, 128.8 (2C), 124.8, 118.4 (2C), 84.1, 81.9, 69.8, 61.6, 39.0, 28.2 (3C), 27.9 (3C), 13.9, 13.8 ppm. IR (ATR): 3322, 2982, 1739, 1710, 1597, 1505, 1391, 1366, 1329, 1245, 1146, 1039, 981, 893, 754 cm^–1^. HRMS (ESI-QTOF) *m*/*z*: [M + Na]^+^ Calcd for C_24_H_34_N_4_NaO_7_ 513.2320; Found 513.2334. HPLC: Lux i-Cellulose-5 column, hexane/i-PrOH 95:5, 1.0 mL/min, λ = 254 nm. Major enantiomer (*R*): *t*_*R*_ = 8.28 min, minor enantiomer (*S*): *t*_*R*_ = 12.13 min, (97:3 er).

#### Synthesis of Linear Polymer LP-**I**^14^

An oven-dried three-neck 250 mL bottom flask, equipped with a mechanical stirrer and blanketed by nitrogen, was charged with the dried monomers, BP (6.29 g, 40.8 mmol, 1.0 equiv), and isatin (6.00 g, 40.8 mmol, 1.0 equiv). The mixture was dissolved in anhydrous chloroform (30 mL), stirred at room temperature for 15 min, and cooled at 0 °C. Then, cold TFSA (60 mL, 679.6 mmol, 16.6 equiv) was added dropwise, and the mixture was left to warm up to room temperature and maintained with mechanical stirring for 10 h. Then, the viscous solution was poured into a 2:1 mixture of MeOH/water. The white threads were neutralized in basic water (pH around 10) and washed sequentially with distilled water, warm distilled water, methanol, and warm methanol. The product was dried at 150 °C under a 60 mbar dynamic vacuum for 24 h. The material was obtained as white threads in quantitative yield (11.50 g, 99.6%). ^1^H NMR (400 MHz, DMSO-*d*_6_) δ 10.82 (s, 1H), 7.65–7.50 (m, 4H), 7.36–7.17 (m, 6H), 7.05–6.93 (m, 2H). ^13^C NMR (101 MHz, DMSO-*d*_6_) δ 178.0, 141.4, 141.0 (2C), 138.5 (2C), 132.9 (2C), 128.6 (4C), 126.8 (4C), 126.1, 122.1, 110.1, 61.7. IR (ATR): 3392, 1716, 1620, 1599, 1495, 1471, 1391, 1320, 1189, 1005, 809, 746 cm^–1^. Inherent viscosity (DMAc, 5 mL/mg): 0.834 dL/g.

### Preparation of Polymer LP-**II**

To a solution of LP-**I** (3.0 g, 10.59 mmol) in dry NMP (50 mL), with magnetic stirring and nitrogen blanketed, at 65 °C was charged K_2_CO_3_ (2.2 g, 16.09 mmol). The mixture was let to react for 2 h, and subsequently, 2-(2-bromoethyl)isoindoline-1,3-dione (4.1 g, 16.14 mmol) was added and the mixture was stirred for 72 h. Afterward, when the mixture reached room temperature, distilled water was added and the suspension was filtered. The filtered product was washed sequentially with distilled water, a 1:1 mixture of H_2_O/MeOH, MeOH, and acetone. The functionalized polymer LP-**II** was dried at 60 °C under 60 mbar vacuum for 16 h, obtaining a white powder (3.7 g) functionalized at 65% according with ^1^H NMR experiments (see the SI). ^1^H NMR (400 MHz, DMSO-*d*_6_) δ 10.84 (s, 1H, 35% free NH (LP-**I**), 7.81–6.82 (m, 22H), 4.26–3.79 (m, 4H, 65% LP-**II**)). ^13^C{^1^H} NMR (101 MHz, DMSO-*d*_6_) δ 178.0 (LP-**I**), 176.6, 167.5, 141.7, 141.4 (LP-**I**), 141.0 (LP-**I**), 140.5, 138.3 (LP-**I**), 134.2, 133.0 (LP-**I**), 131.9, 131.3, 128.9 (LP-**I**), 128.6, 126.8 (LP**-I**), 126.5, 126.1, 123.0, 122.1, 110.1, 109.3, 61.7 (LP-**I**), 61.1, 38.2, 34.9. IR (ATR): 2985, 1772, 1714, 1608, 1469, 1391, 1355, 1193, 1142, 1098, 1003, 813, 747, 721 cm^–1^.

### Preparation of Polymer LP-**III**

To a solution of polymer LP-**II** (3.2 g, 8.1 mmol) in dry NMP (50 mL), with magnetic stirring and nitrogen blanketed, at 40 °C was charged hydrazine hydrate (2.5 mL, 80.1 mmol, 10.0 equiv). The mixture was stirred at 40 °C for 24 h. Afterward, the reaction mixture was poured over distilled water and the suspension was filtered. The solid was washed sequentially with distilled water, a warmed 1:1 mixture of H_2_O/MeOH, MeOH, and acetone. The polymer LP-**III** was dried at 50 °C under 60 mbar vacuum for 16 h, obtaining a white powder (2.4 g, 65% *N*-ethyl-amino LP-**III**). ^1^H NMR (500 MHz, DMSO-*d*_6_, 60 °C) δ 10.82 (br s, 1H, estimated remained 35% free NH of LP-**I**), 7.95–6.71 (m, 16H), 3.75 (br s, 2H), 2.80 (br s, 2H). ^13^C{^1^H} NMR (126 MHz, DMSO-*d*_6_, 60 °C) δ 177.7, 176.3, 142.3, 141.3, 140.8, 140.6, 138.35, 138.32, 138.2, 132.8, 132.0, 128.4, 126.5, 125.8, 125.6, 122.3, 121.8, 109.9, 109.3, 61.5, 61.1, 42.8. IR (ATR): 3649, 2977, 2919, 1706, 1607, 1494, 1472, 1355, 1248, 1190, 1007, 809, 750 cm^–1^.

### Preparation of Polymer LP-**IV**

To a solution of LP-**III** (0.5 g, 1.61 mmol, 65% of NH_2_ groups) in dry DMSO (50 mL), with magnetic stirring and nitrogen blanketed, at 60 °C was charged QN-NCS (0.9 g, 2.46 mmol, 1.5 equiv). The mixture was stirred for 72 h at 50 °C. Afterward, the reaction mixture was poured over iced distilled water and the suspension was filtered. The solid was washed sequentially with distilled water, a warmed 1:1 mixture of H_2_O/MeOH, MeOH, and acetone. The polymer LP-**IV** was dried at 50 °C under 60 mbar vacuum for 16 h, obtaining a white powder (0.46 g). ^1^H NMR (500 MHz, DMSO-*d*_6_, 60 °C) δ 10.86 (br s, 1H, estimated remained 35% free NH of LP-**I**), 8.65 (s, 1H), 8.14 (br s; 1H), 7.97–7.83 (m, 2H), 7.80–6.85 (m, 16H),5.97 (br s, 1H), 5.81–5.61 (m, 2H), 5.01–4.72 (m, 2H), 3.88 (s, 3H), 4.20–3.48 (m, 3H), 3.29–3.00 (m 3H), 2.41–2.07 (m, 2H), 1.81–0.53 (m, 6H). ^13^C{^1^H} NMR (126 MHz, DMSO-*d*_6_, 60 °C) δ 182.8, 178.0, 176.5, 157.2, 147.6, 145.9, 144.3, 142.6, 141.8, 141.6, 141.1, 140.83, 140.78, 138.6, 133.1, 132.1, 131.2, 128.7, 128.1, 126.8, 126.1, 126.0, 125.8, 122.6, 122.1, 121.1, 120.6, 114.2, 110.2, 109.5, 103.6, 61.8, 61.3, 59.6, 55.7, 55.4, 41.8, 41.1, 38.9, 32.0, 29.7, 27.4, 27.2, 25.5. IR (ATR): 3315, 2934, 1710, 1622, 1509, 1490, 1468, 1351, 1226, 1193, 1032, 1006, 919, 820, 747 cm^–1^. Inherent viscosity (DMAc, 5 mL/mg): 0.749 dL/g. The effective functionalization, *f* = 1.26 mmol g^–1^, was calculated based on sulfur elemental analysis: C: 70.74, H: 5.84, N: 8.36, S: 4.03.

### General Procedure for the Enantioselective Amination of Pyrazolone **9a** with Di-*tert*-butyl-azodicarboxylate using Heterogeneous Catalysts in Batch Conditions

A 20 mol % suspension of the heterogeneous catalyst (LP**-III**, LP**-IV**, or **V**) in toluene (1 mL) was stirred at room temperature for 20 min, and then pyrazolone **9a** (0.1 mmol) and di-*tert*-butyl-azodicarboxylate **12** (0.12 mmol, 1.2 equiv) were sequentially added. The mixture was stirred until the reaction was finished (TLC). The catalyst was collected by centrifugation (4500 rpm) and washed with toluene (2 × 0.5 mL). The filtrate was concentrated under reduced pressure, and the residue was purified by flash column chromatography (silica gel, hexane/EtOAc: 8/1) to give the pure amination product **13a**. In the recycled experiments with LP**-IV** (entries 6–10 in Table 2), the catalyst was washed with toluene, dried under vacuum at 50 °C until constant weight, and reused in the next reaction. The enantiomeric ratio was determined by chiral-phase HPLC analysis using mixtures of hexane/i-PrOH as an eluent.

### Experimental Setup for the Continuous Flow Amination of Pyrazolone **9a** with Di-*tert*-butyl-azodicarboxylate using the Heterogeneous Catalysts LP-**IV**

For the continuous flow experiments, the instrumental setup is schematized in Table 3. The packed bed reactor consisted of a vertically mounted Omnifit column (6.6 internal diameter and 50 mm length) containing the LP-**IV** (300 mg, *f* = 1.26 mmol g^–1^, 0.38 mmol). The reactor inlet was connected to a THALESNano micro HPLC pump. First, a 1:1 mixture of toluene/DCM was flushed for 60 min at 0.2 mL/min flow rate to swell the catalyst. After that, the channel was fed with a solution of pyrazolone **9a** (1.07 g, 4.0 mmol, 1.0 equiv, 0.15 M) and di-*tert*-butyl azodicarboxylate (**12**) (0.93 g, 4.0 mmol, 1.0 equiv, 0.15 M) in toluene/DCM 1:1 (27 mL), which was pumped through the reactor at 0.15 mL/min flow rate. The reactor outlet was connected to a flask, where the product was collected. The system was running for 3 h, and the catalyst was washed with toluene for 60 min at 0.2 mL/min flow rate. The sample was collected, and the solvent was removed under reduced pressure. The crude was purified by flash column chromatography on silica gel (hexane/EtOAc: 8/1) to afford the final pure product **13a** in 86% isolated yield (1.72 g, 3.48 mmol, er 88:12). Productivity: 3.05 mmol prod mmol cat^–1^ h^–1^; TON: 9.1, residence time: 10 min.

### Di-*tert*-butyl (*R*)-1-(4-([1,1′-Biphenyl]-4-ylmethyl)-3-methyl-5-oxo-1-phenyl-4,5-dihydro-1*H*-pyrazol-4-yl)hydrazine-1,2-dicarboxylate (**16**)

To a solution of **15g** (57 mg, 0.1 mmol), phenylboronic acid (18 mg, 0.15 mmol), and K_3_PO_4_ (43 mg, 0.2 mmol) in THF/H_2_O: 5/1 (1.5 mL) under a N_2_ atmosphere, PdCl_2_(PPh_3_)_2_ (8 mg, 0.01 mol) was added. After refluxing for 3 h, the solvent was removed under reduced pressure and the crude mixture was purified by flash chromatography (hexane/EtOAc: 8/1) affording the pure compound as a pale-yellow oil (50 mg, 0.088 mmol, 88% yield).  = −172.4 (*c* = 0.5, CHCl_3_). ^1^H NMR (400 MHz, CDCl_3_) δ 7.46–7.27 (m, 9H), 7.23 (t, *J* = 7.9 Hz, 2H), 7.14 (d, *J* = 8.1 Hz, 2H), 7.07 (t, *J* = 7.4 Hz, 1H), 6.81 (br s, 1H), 3.38 (d, *J* = 12.6 Hz, 1H), 3.08 (d, *J* = 12.6 Hz, 1H), 2.40 (s, 3H), 1.59–1.47 (m, 9H), 1.43–1.21 (m, 9H) ppm. ^13^C{^1^H} NMR (101 MHz, CDCl_3_) δ 172.1, 160.3 (2C), 156.1, 153.0, 140.6, 137.3, 130.4, 130.1 (2C), 128.6 (2C), 128.5 (2C), 127.2 (2C), 127.0 (2C), 126.8 (2C), 125.1, 119.4, 81.8, 73.6, 38.7, 31.6, 28.2 (3C), 28.0 (3C), 14.5 ppm. IR (ATR): 3282, 2981, 2934, 1703, 1597, 1501, 1487, 1366, 1329, 1245, 1150, 1113, 977, 849, 758, 743 cm^–1^. HRMS (ESI-QTOF) *m*/*z*: [M + H]^+^ Calcd for C_33_H_39_N_4_O_5_ 571.2915; Found 571.2926. HPLC: Lux i-Cellulose-5 column, hexane/i-PrOH 97:3, 1.0 mL/min, λ = 254 nm. Major enantiomer (*R*): *t*_*R*_ = 9.58 min, minor enantiomer (*S*): *t*_*R*_ = 18.02 min, (93:7 er).

## Data Availability

The data underlying this study are available in the published article and its Supporting Information.

## Supporting Information Available

Copies of ^1^H and ^13^C NMR spectra for new compounds and polymers, IR spectra, TGA of polymers, swelling ratio of polymer LP-**IV**, and HPLC profiles of all new compounds (PDF)

## References

[ref1] Zhao Z.; Dai X.; Li C.; Wang X.; Tian J.; Feng Y.; Xie J.; Ma C.; Nie Z.; Fan P.; Qian M.; He X.; Wu S.; Zhang Y.; Zheng X. Pyrazolone Structural Motif in Medicinal Chemistry: Retrospect and Prospect. Eur. J. Med. Chem. 2020, 186, 111893–111918. 10.1016/j.ejmech.2019.111893. 31761383 PMC7115706

[ref2] aChauhan P.; Mahajan S.; Enders D. Asymmetric Synthesis of Pyrazoles and Pyrazolones Employing the Reactivity of Pyrazolin-5-one Derivatives. Chem. Commun. 2015, 51, 12890–12907. 10.1039/C5CC04930J. 26178319

[ref3] aWang W.; Wei S.; Bao X.; Nawaz S.; Qu J.; Wang B. Enantioselective [3 + 2] Annulation of 4-Isothiocyanato Pyrazolones and Alkynyl Ketones under Organocatalysis. Org. Biomol. Chem. 2021, 19, 1145–1154. 10.1039/D0OB02423F. 33449059

[ref4] Mahajan S.; Chauhan P.; Kaya U.; Deckers K.; Rissanen K.; Enders D. Enantioselective Synthesis of Pyrazolone α-Aminonitrile Derivatives via an Organocatalytic Strecker Reaction. Chem. Commun. 2017, 53, 6633–6636. 10.1039/C7CC02874A. 28585622

[ref5] Chauhan P.; Mahajan S.; Kaya U.; Peuronen A.; Rissanen K.; Enders D. Asymmetric Synthesis of Amino-Bis-Pyrazolone Derivatives via an Organocatalytic Mannich Reaction. J. Org. Chem. 2017, 82, 7050–7058. 10.1021/acs.joc.7b01113. 28541704

[ref6] aKaya U.; Chauhan P.; Mahajan S.; Deckers K.; Valkonen A.; Rissanen K.; Enders D. Squaramide-Catalyzed Asymmetric aza-Friedel-Crafts/N,O-Acetalization Domino Reactions Between 2-Naphthols and Pyrazolinone Ketimines. Angew. Chem., Int. Ed. 2017, 56, 15358–15362. 10.1002/anie.201709224. 29044902

[ref7] Yang Z.; Wang Z.; Bai S.; Liu X.; Lin L.; Feng X. Asymmetric α-Amination of 4-Substituted Pyrazolones Catalyzed by a Chiral Gd(OTf)3/N,N′-Dioxide Complex: Highly Enantioselective Synthesis of 4-Amino-5-pyrazolone Derivatives. Org. Lett. 2011, 13, 596–599. 10.1021/ol102804p. 21214254

[ref8] aŠimek M.; Remeš M.; Veselý J.; Rios R. Enantioselective Organocatalytic Amination of Pyrazolones. Asian J. Org. Chem. 2013, 2, 64–68. 10.1002/ajoc.201200168.

[ref9] aBenaglia M.; Puglisi A. Catalyst immobilization: Methods and Applications. Wiley-VCH, 2020 10.1002/9783527817290.

[ref10] aKasaplar P.; Ozkal E.; Rodríguez-Escrich C.; Pericàs M. Enantioselective α-Amination of 1,3-Dicarbonyl Compounds in Batch and Flow with Immobilized Thiourea Organocatalysts. Green Chem. 2015, 17, 3122–3129. 10.1039/C5GC00496A.

[ref11] aRodríguez-Rodríguez M.; Maestro A.; Andrés J. M.; Pedrosa R. Supported Bifunctional Chiral Thioureas as Catalysts in the Synthesis of 3-Amino-2-Oxindoles through Enantioselective aza-Friedel-Crafts Reaction: Application in Continuous Flow Processes. Adv. Synth. Catal. 2020, 362, 2744–2754. 10.1002/adsc.202000238.

[ref12] aMatesanz-Niño L.; Esteban N.; Webb M. T.; Martínez-Gómez A.; Suárez-García F.; González-Ortega A.; Miguel J. A.; Palacio L.; Galizia M.; Álvarez C.; Lozano A. E. Polymer Materials Derived from the SEAr Reaction for Gas Separation Applications. Polymer 2023, 267, 125647–125658. 10.1016/j.polymer.2022.125647.

[ref13] Klumpp D. A.; Yeung K. Y.; Surya Prakash G. K.; Olah G. A. Preparation of 3,3-Diaryloxindoles by Superacid-Induced Condensations of Isatins and Aromatics with a Combinatorial Approach. J. Org. Chem. 1998, 63, 4481–4484. 10.1021/jo980588g.

[ref14] aCruz A. R.; Hernandez M. C. G.; Guzmán-Gutiérrez M. T.; Zolotukhin M. G.; Fomine S.; Morales S. L.; Kricheldorf H.; Wilks E. S.; Cárdenas E. J.; Salmón M. Precision Synthesis of Narrow Polydispersity, Ultrahigh Molecular Weight Linear Aromatic Polymers by A2 + B2 Nonstoichiometric Step-Selective Polymerization. Macromolecules 2012, 45, 6774–6780. 10.1021/ma301691f.

[ref15] aBaumann M.; Moody T. S.; Smyth M.; Wharry S. A Perspective on Continuous Flow Chemistry in the Pharmaceutical Industry. Org. Process Res. Dev. 2020, 24, 1802–1813. 10.1021/acs.oprd.9b00524.

[ref16] Yu X.-M.; Ramiandrasoa F.; Guetzoyan L.; Pradines B.; Quintino E.; Gadelle D.; Forterre P.; Cresteil T.; Mahy J.-P.; Pethe S. Synthesis and Biological Evaluation of Acridine Derivatives as Antimalarial Agents. ChemMedChem. 2012, 7, 587–605. 10.1002/cmdc.201100554. 22331612

[ref17] Lai Q.; Li Y.; Gong Z.; Liu Q.; Wei C.; Song Z. Novel Chiral Bifunctional L-Thiazoline-Thiourea Derivatives: Design and Application in Enantioselective Michael Reactions. Chirality 2015, 27, 979–988. 10.1002/chir.22540. 26427336

[ref18] Andrés J. M.; Maestro A.; Rodríguez-Ferrer P.; Simón I.; Pedrosa R. Short Synthesis of Novel Recyclable Chiral Bifunctional Thioureas from Aminoalkyl Polystyrene and their use as Organocatalysts in Stereoselective aza-Henry Reaction. ChemistrySelect 2016, 1, 5057–5061. 10.1002/slct.201601213.

[ref19] Opalka S. M.; Steinbacher J. L.; Lambiris B. A.; McQuade D. T. Thiourea/Proline Derivative-Catalyzed Synthesis of Tetrahydrofuran Derivatives: A Mechanistic View. J. Org. Chem. 2011, 76, 6503–6517. 10.1021/jo200838v. 21657272

