# Supplementary material for: Enantioselective Amination of 4-Substituted Pyrazolones Catalyzed by Oxindole-Containing Thioureas and by a Recyclable Linear-Polymer-Supported Analogue in a Continuous Flow Process
Source: J Org Chem. 2023 Dec 14;89(1):330–44. doi: 10.1021/acs.joc.3c02069 (PMC10777414; doi:10.1021/acs.joc.3c02069)
Supplement: Supplementary file 1 — jo3c02069_si_001.pdf [file jo3c02069_si_001.pdf]

Supporting Information

Enantioselective Amination of 4-Substituted Pyrazolones Catalyzed by Oxindole-Containing Thioureas and by a Recyclable Linear-Polymer-Supported Analogue in a Continuous Flow Process

Rodrigo Sánchez-Molpeceres,[a] Laura Martín,*[a] Noelia Esteban,[b] Jesús A. Miguel,[b] Alicia Maestro[a] and José M. Andrés*[a]

[a]SintACat, IU CINQUIMA y Departamento de Química Orgánica, Facultad de Ciencias, Universidad de Valladolid, Paseo Belén 7, 47011 Valladolid, Spain.

[b]CLiNuMat, IU CINQUIMA y Departamento de Química Física y Química Inorgánica, Facultad de Ciencias, Universidad de Valladolid, Paseo Belén 7, 47011 Valladolid, Spain.

E-mail: jmandres@uva.es, laura.martinm@uva.es

List of Content

1. NMR Spectra for New Compounds and Polymers…………………………. S2-S33

2. IR Spectra of Polymers LP I-IV………………………………………………… S35

3. TGA Thermograms……………………………………………………………… S36

4. Swelling Ratio of Polymer LP-IV in Several Solvents……………………….... S36

4. HPLC Profiles…………………………………………………………….. S37-S54

1. NMR Spectra for New Compounds and Polymers

^{1}H NMR (400 MHz, Chloroform-d)/ ^{13}C {^{1}H} NMR (101 MHz, Chloroform-d) of **2**

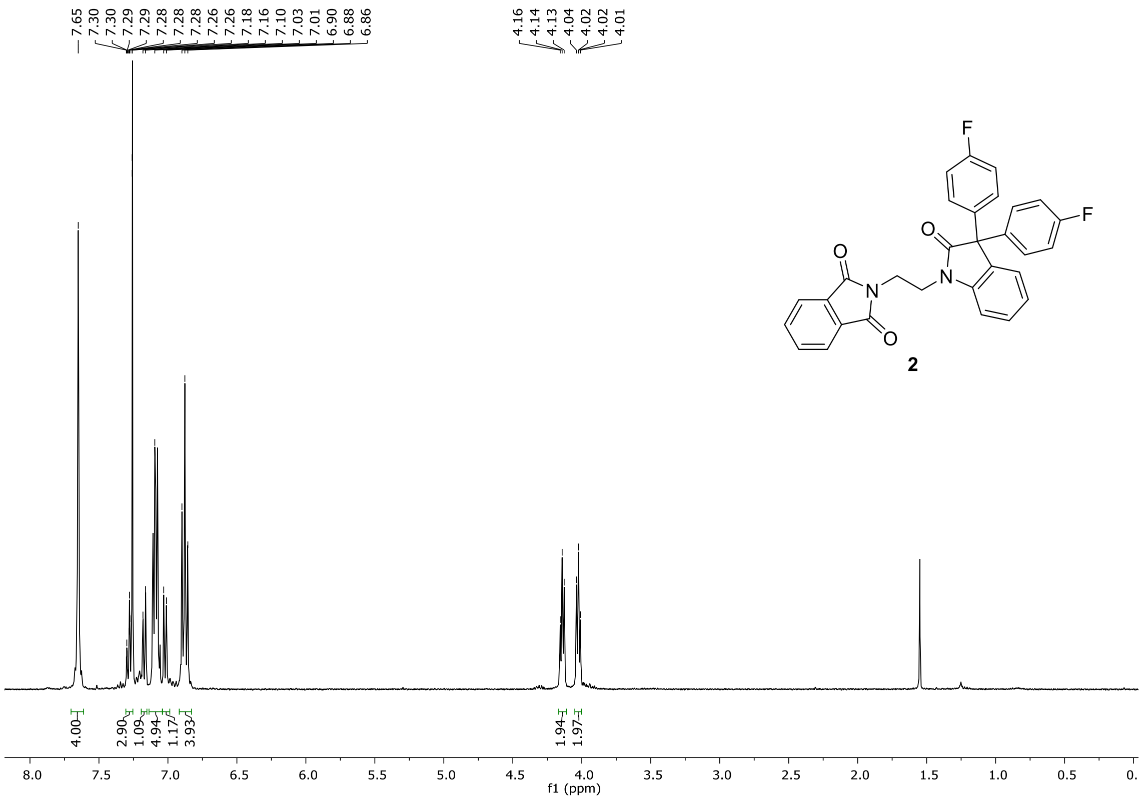

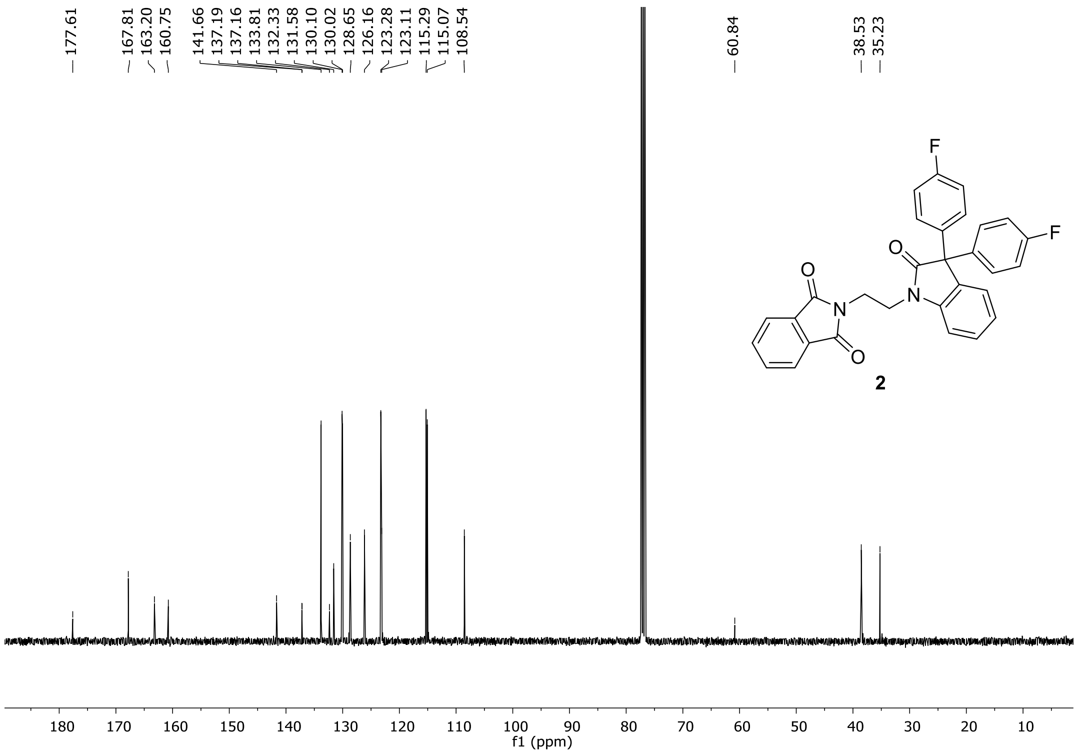

^{1} H NMR (400 MHz, Chloroform-d)/ ^{13}C {^{1} H} NMR (101 MHz, Chloroform-d) of **3**

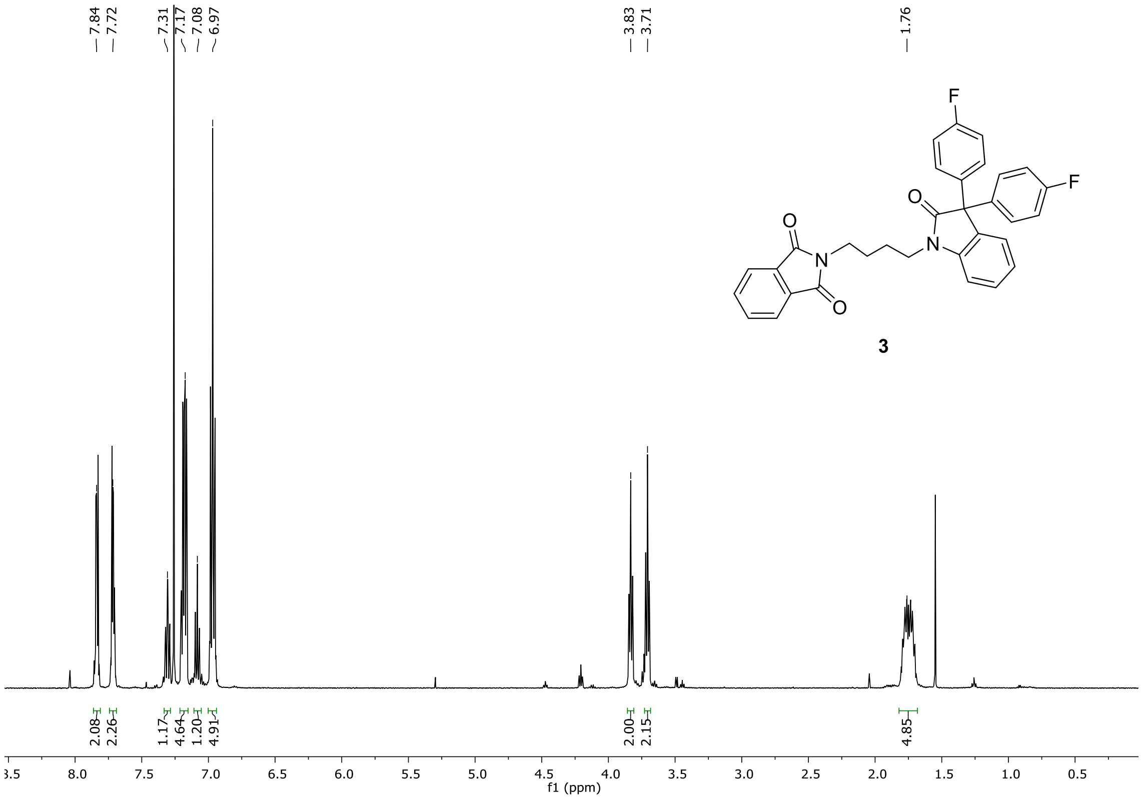

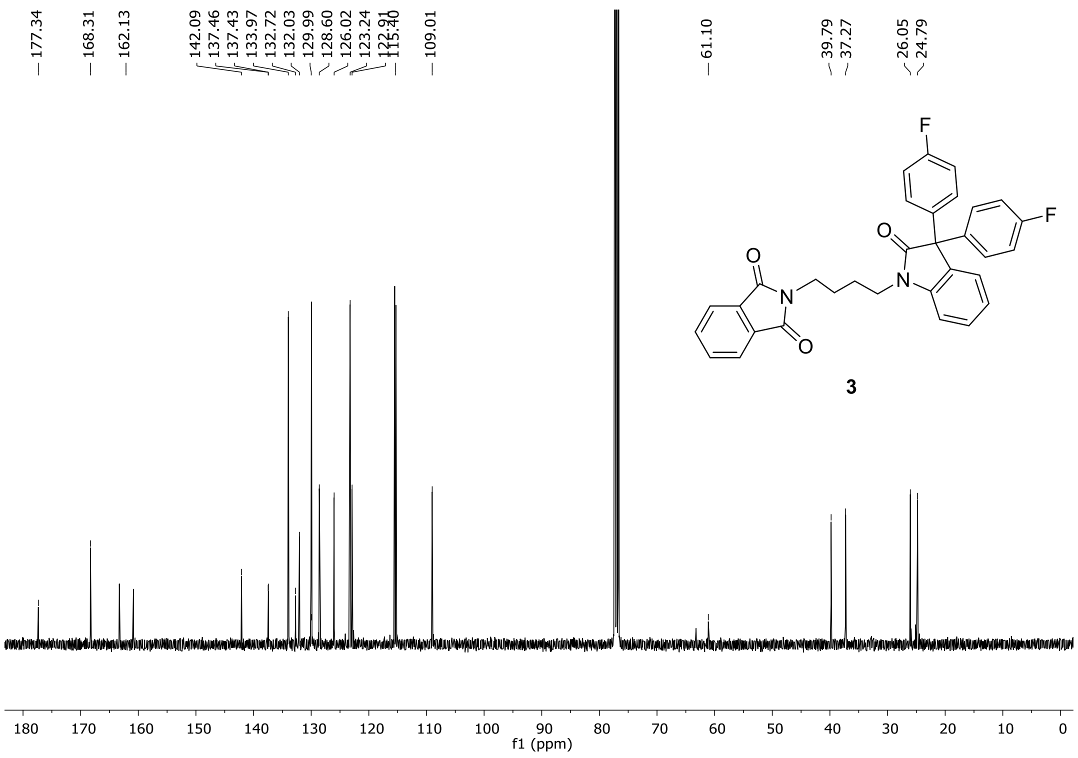

^{1}H NMR (500 MHz, Chloroform-d)/ ^{13}C {^{1}H} NMR (126 MHz, Chloroform-d) of **7**

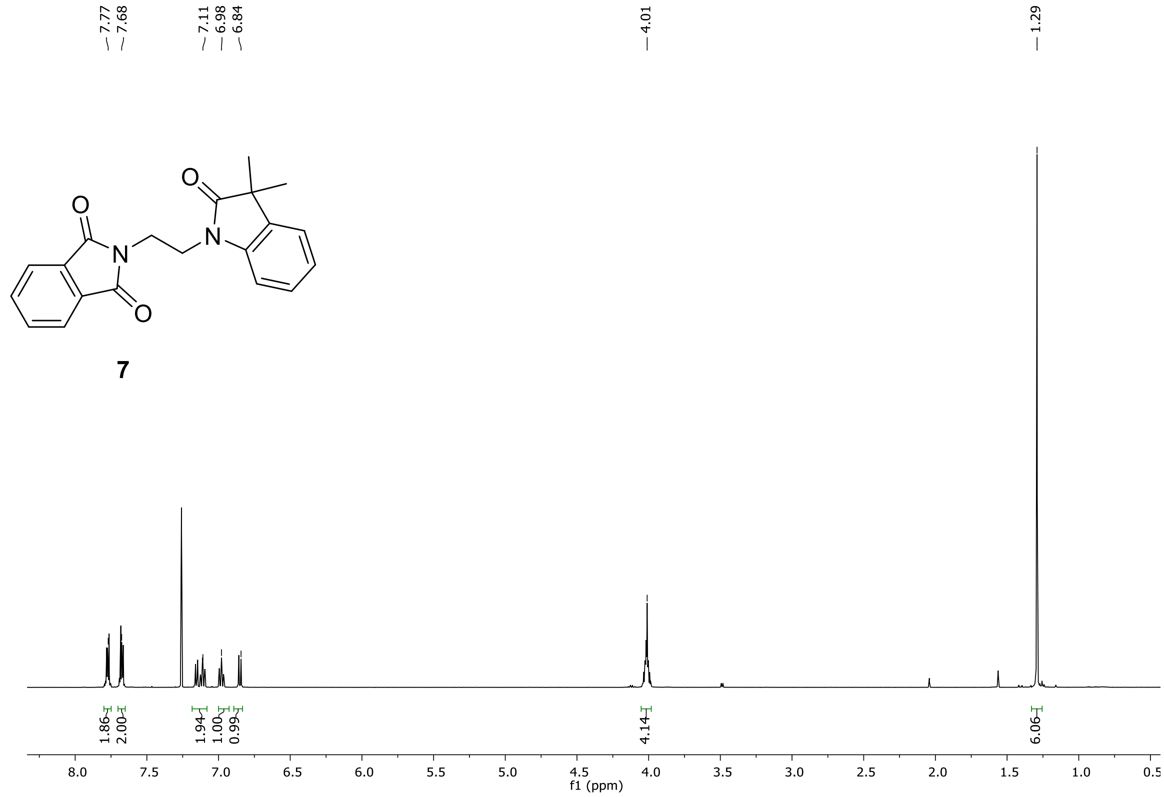

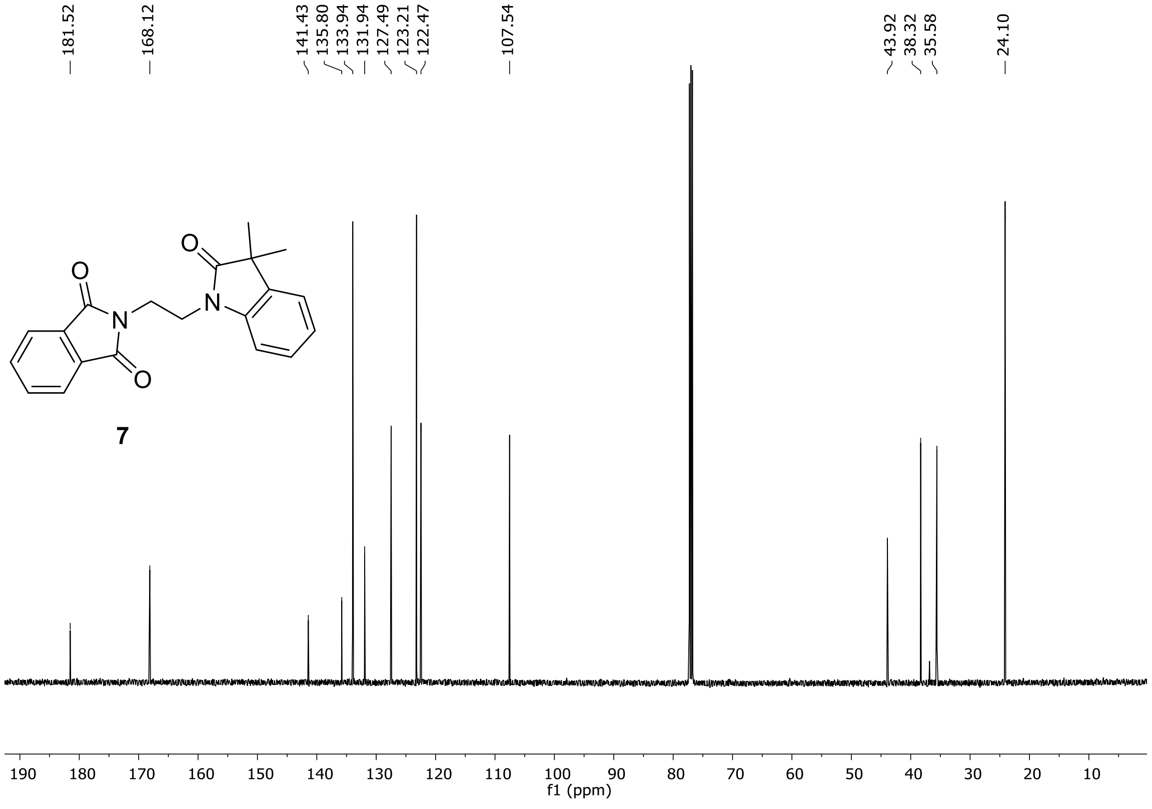

[1] H NMR (400 MHz, Chloroform-d)/ ^{13}C {[1] H} NMR (101 MHz, Chloroform-d) of **4**

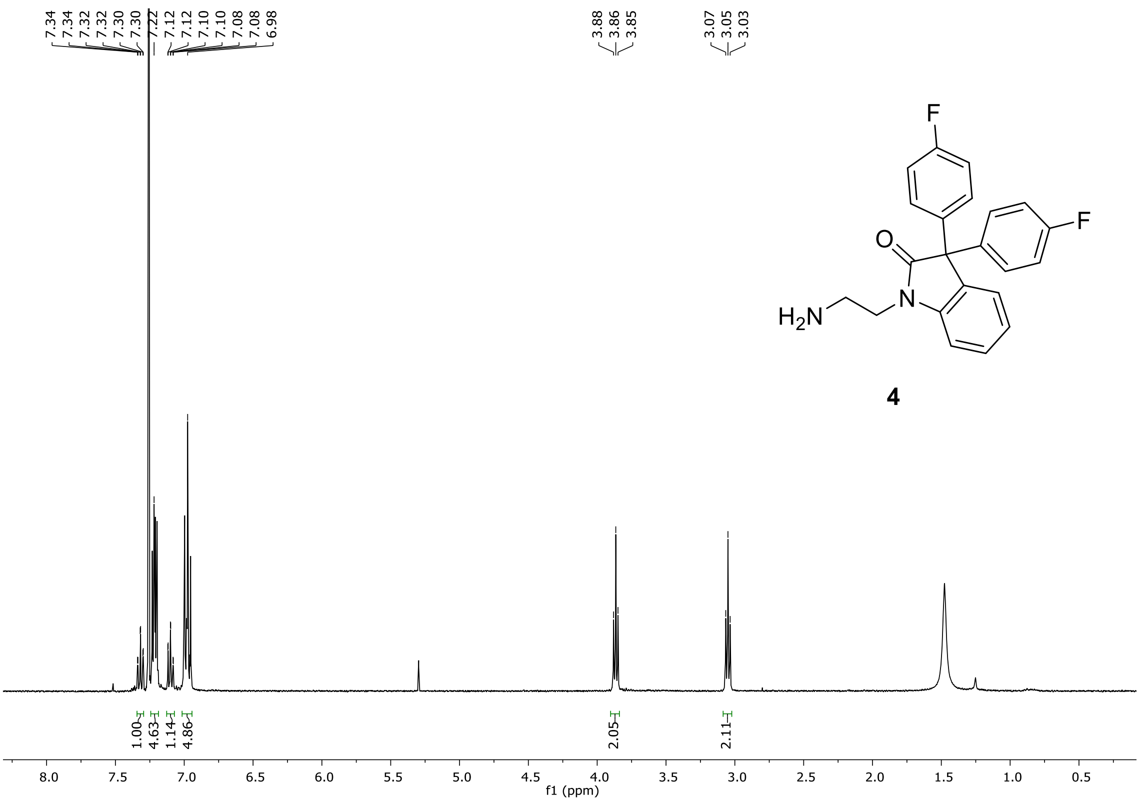

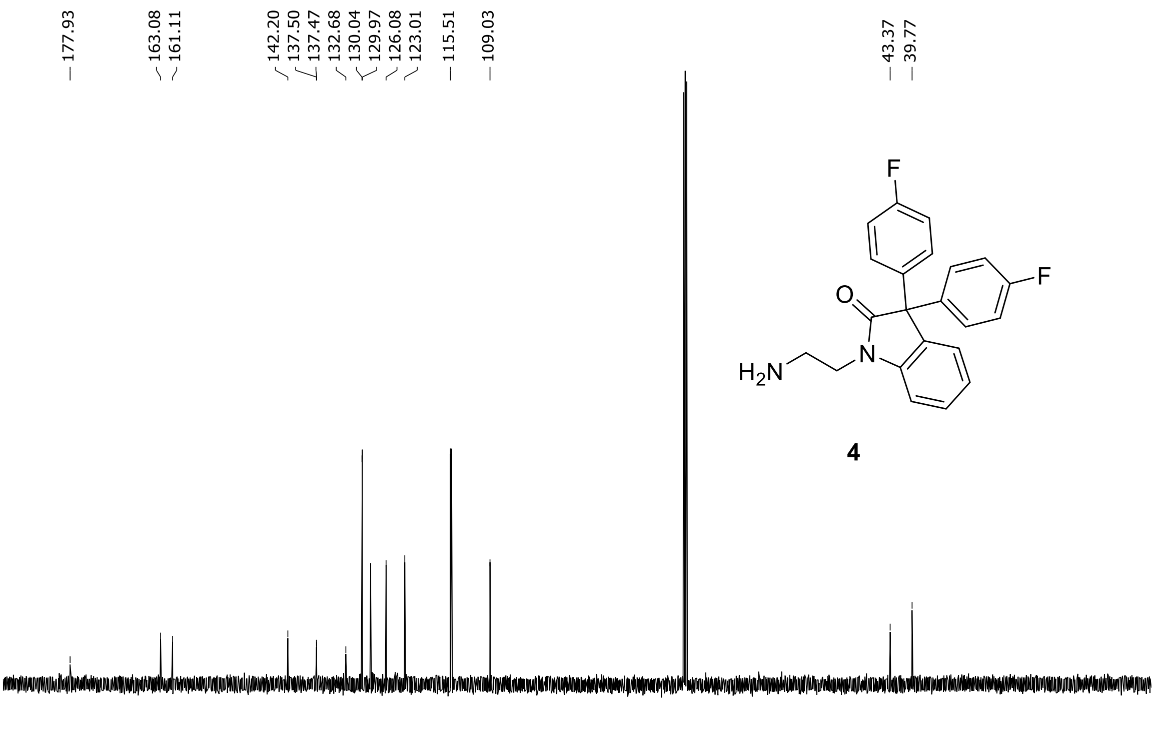

^{1}H NMR (400 MHz, Chloroform-d)/ ^{13}C {^{1}H} NMR (101 MHz, Chloroform-d) of **5**

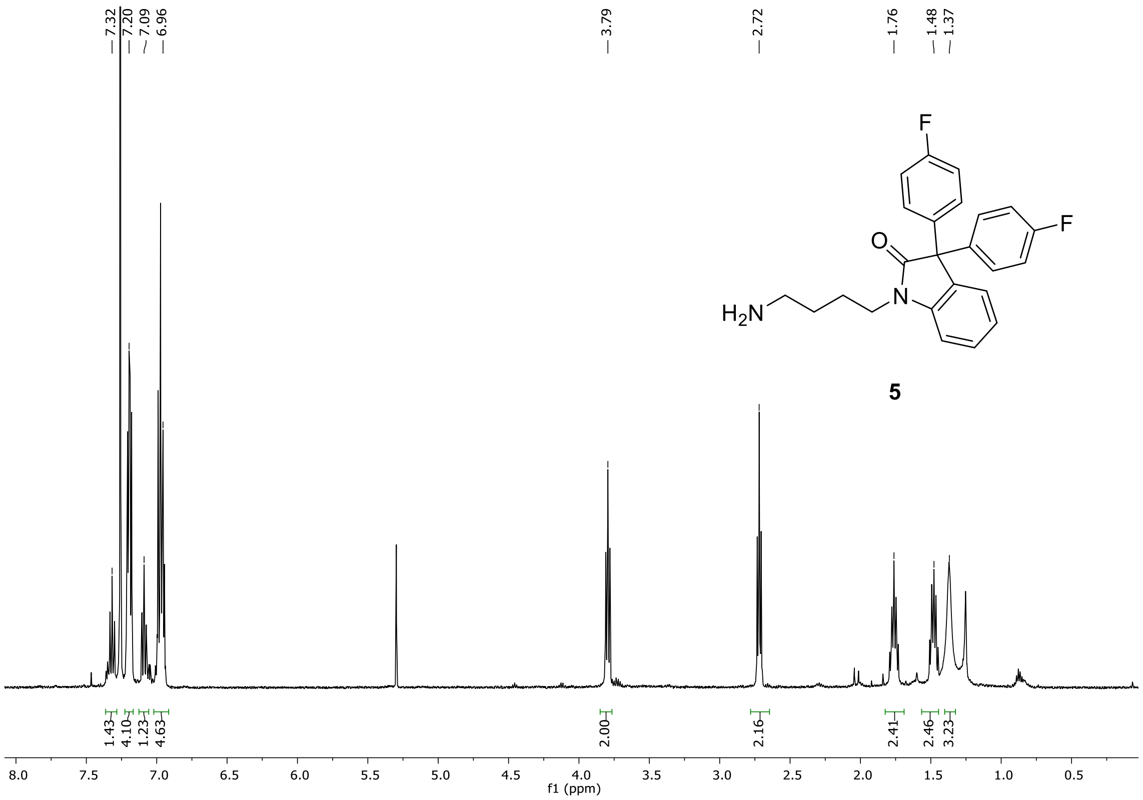

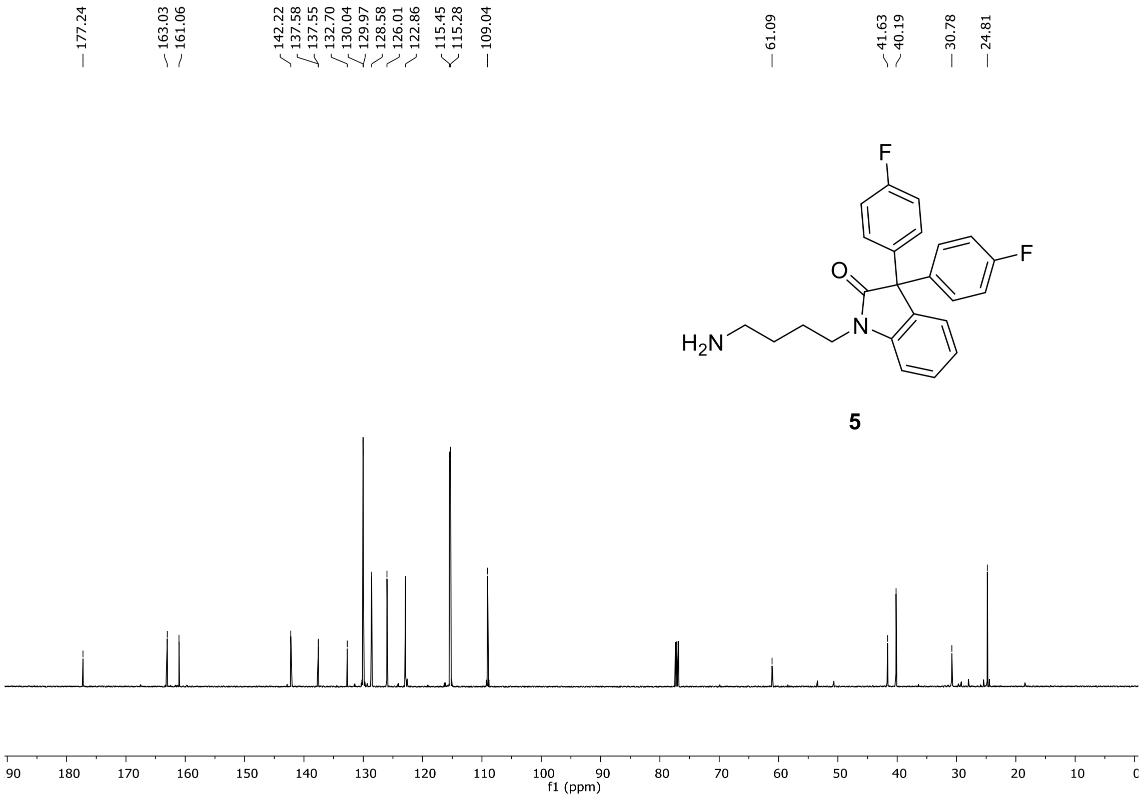

[1] H NMR (500 MHz, Chloroform-d)/ ^{13}C {[1] H} NMR (126 MHz, Chloroform-d) of **8**

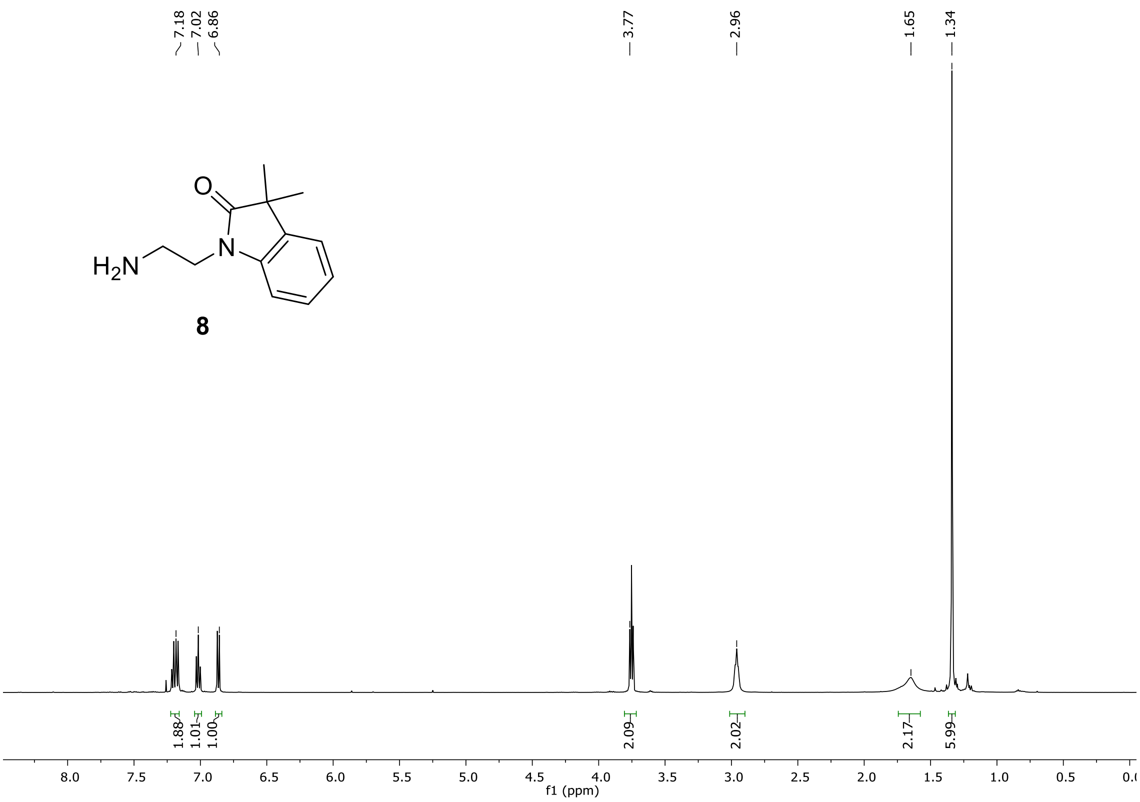

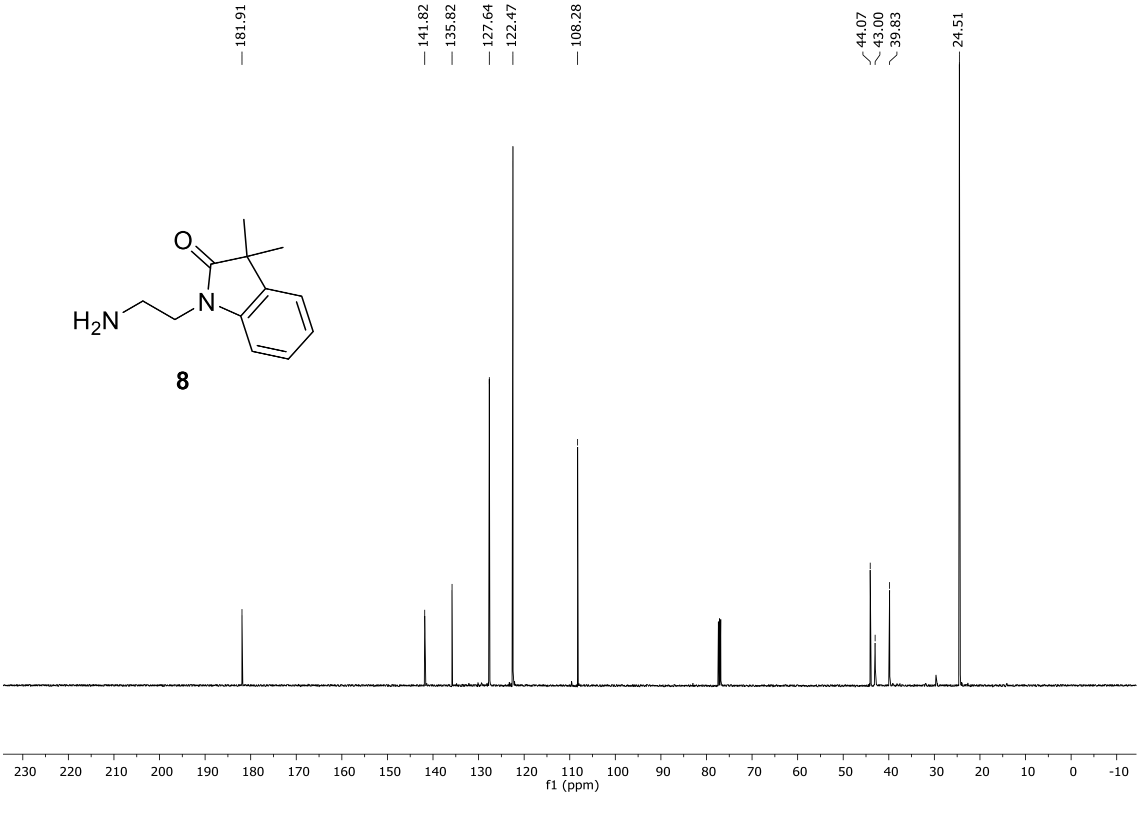

[1] H NMR (400 MHz, Chloroform-d)/ ^{13}C {[1] H} NMR (101 MHz, Chloroform-d) of **C1**

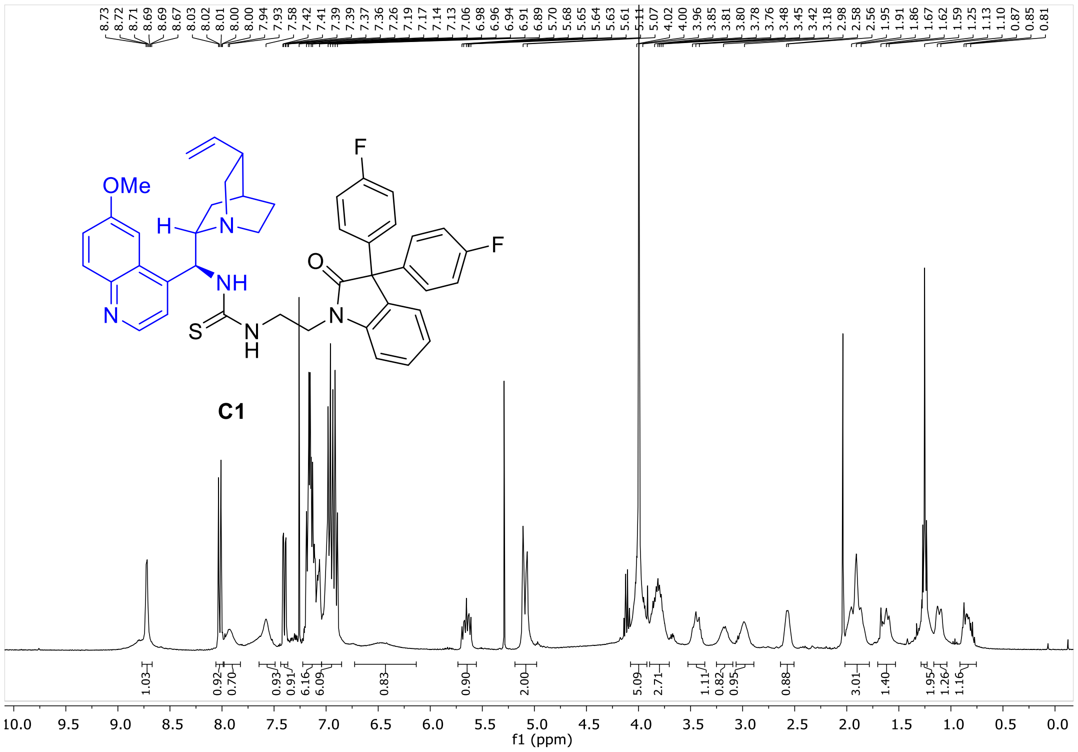

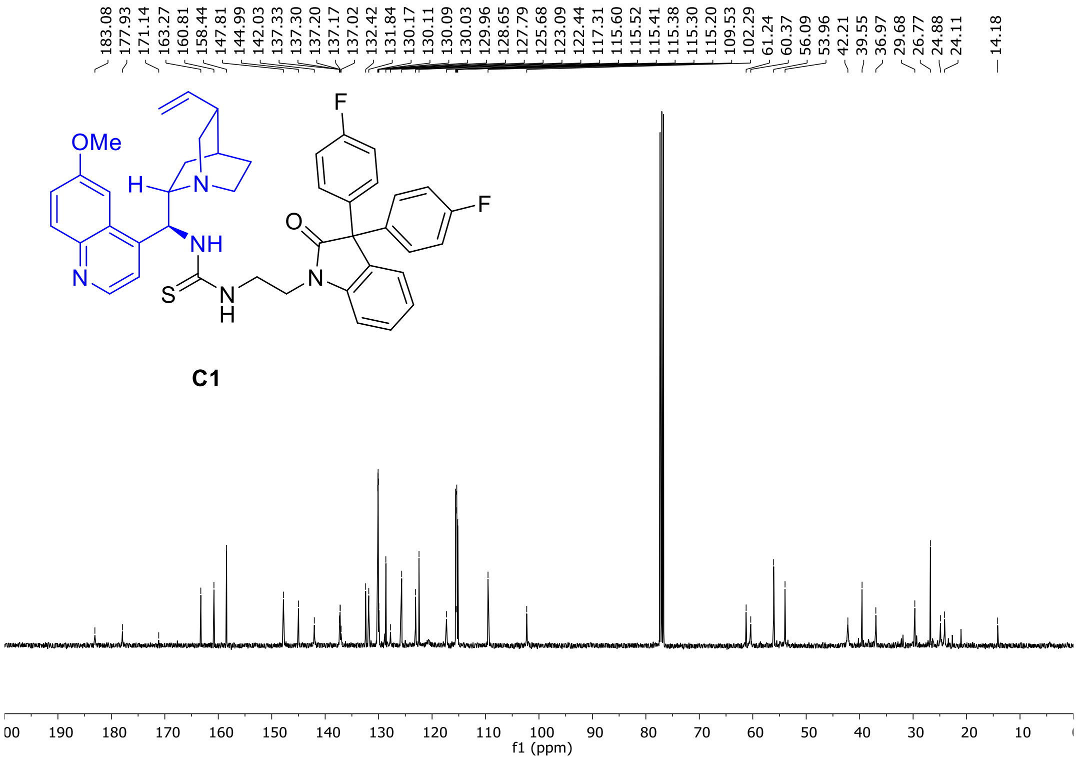

[1] H NMR (400 MHz, Chloroform-d)/ ^{13}C {[1] H} NMR (101 MHz, Chloroform-d) of **C2**

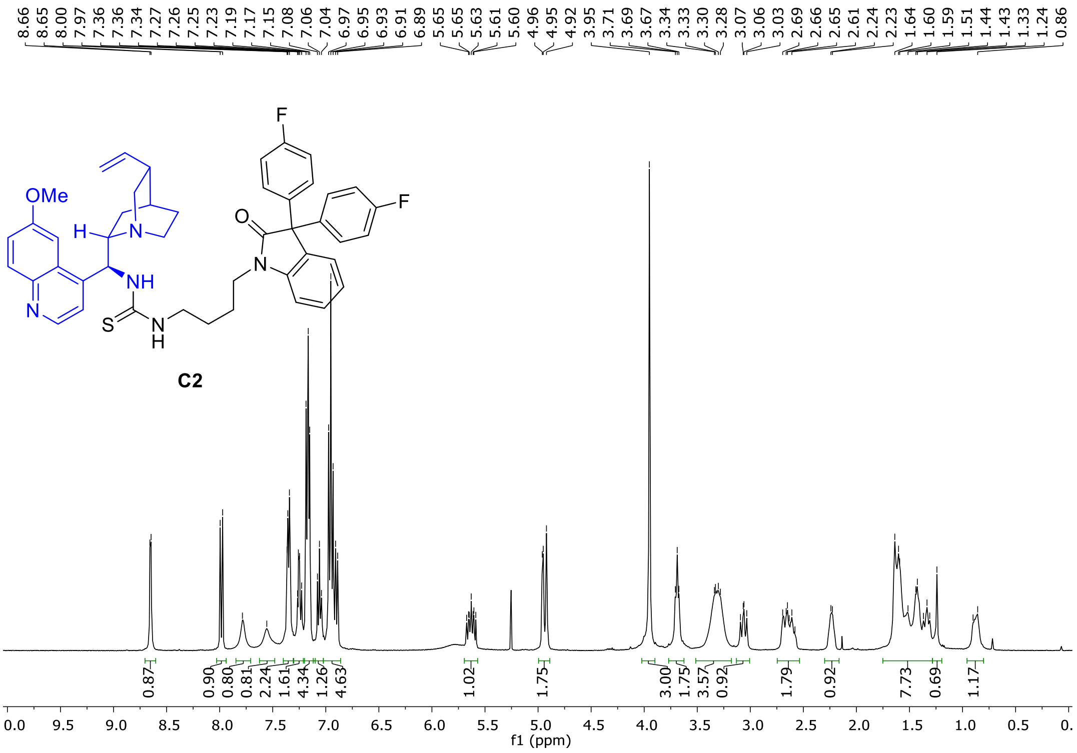

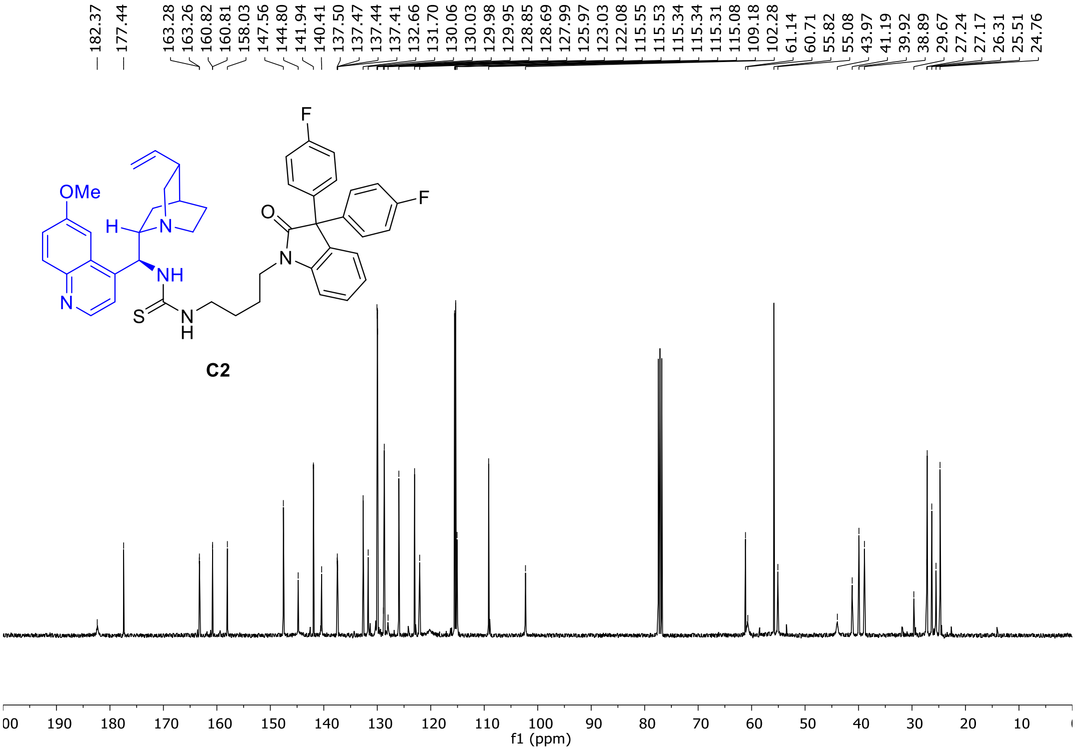

^{1}H NMR (500 MHz, Chloroform-d)/ ^{13}C {^{1}H} NMR (101 MHz, Chloroform-d) of **C3**

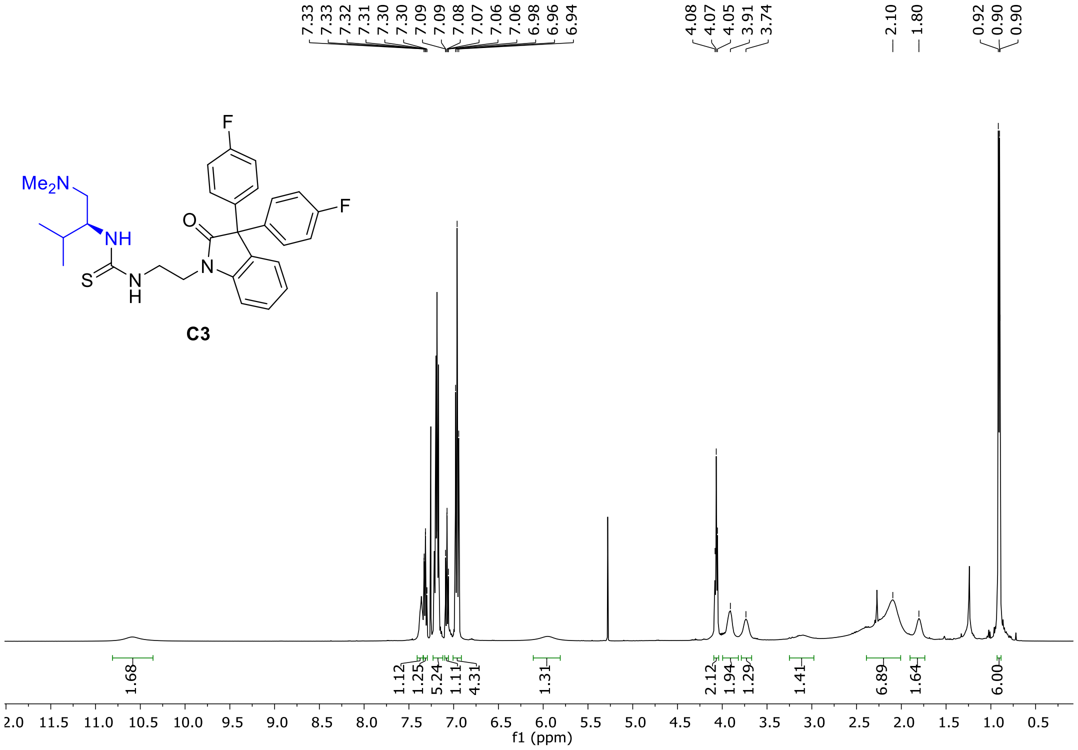

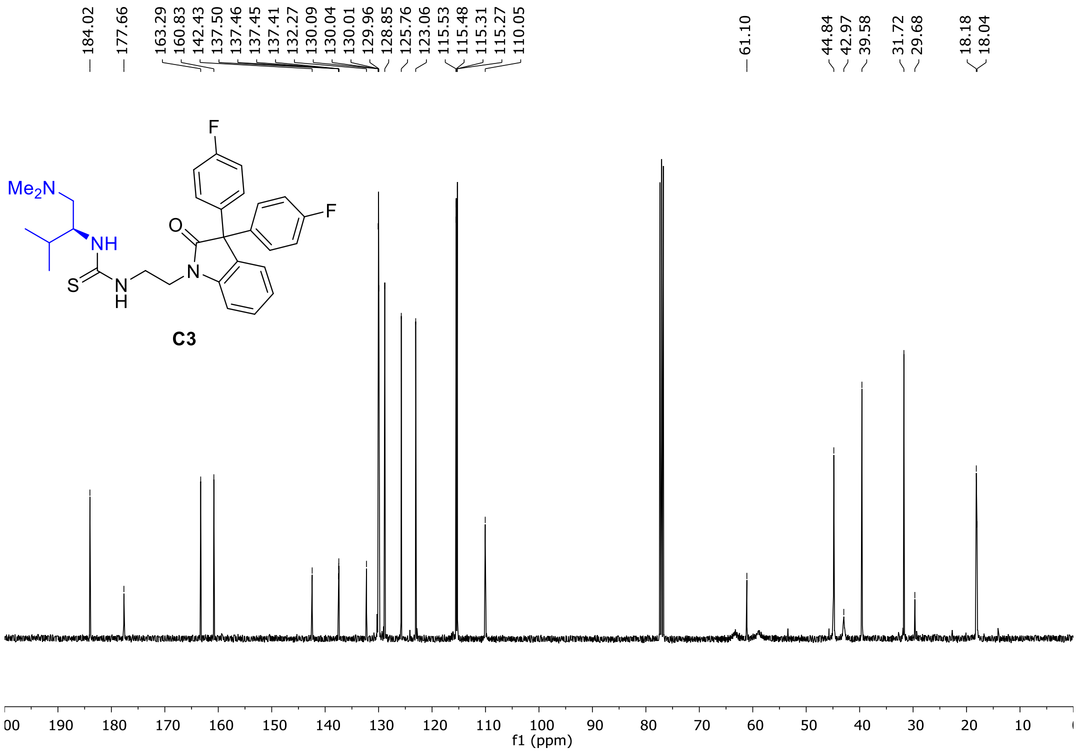

[1] H NMR (500 MHz, Chloroform-d)/ ^{13}C {[1] H} NMR (101 MHz, Chloroform-d) of **C4**

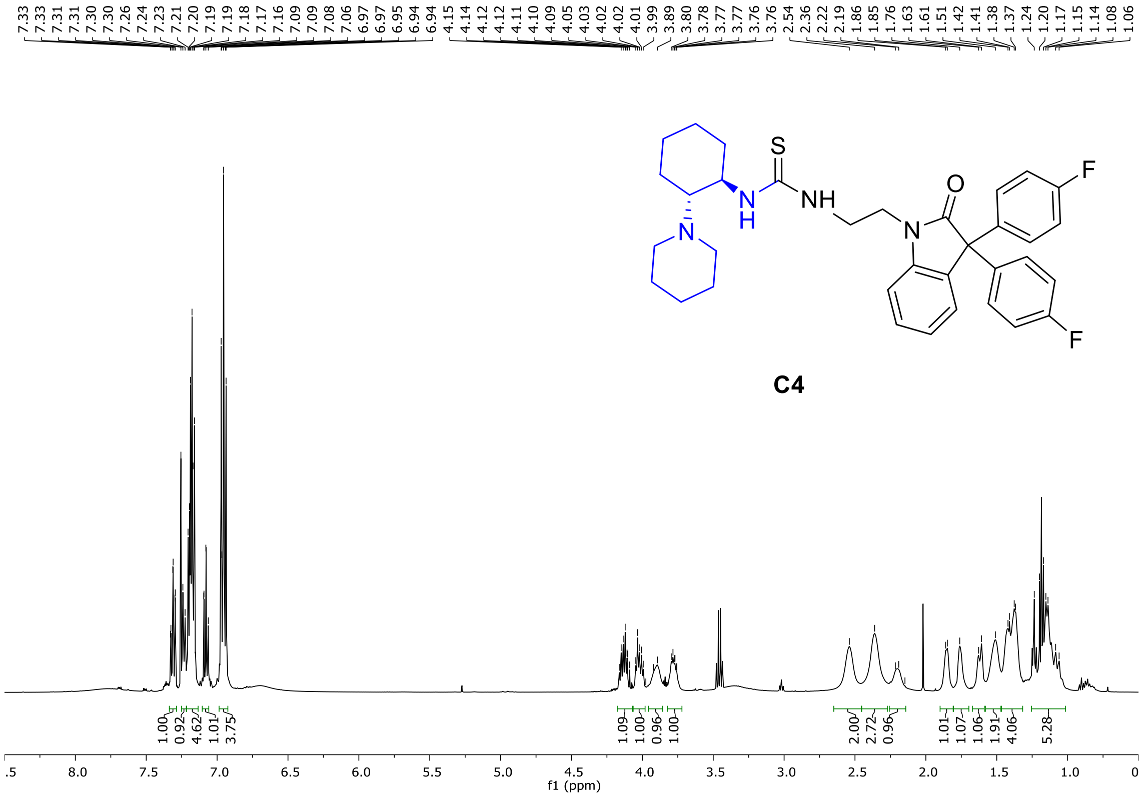

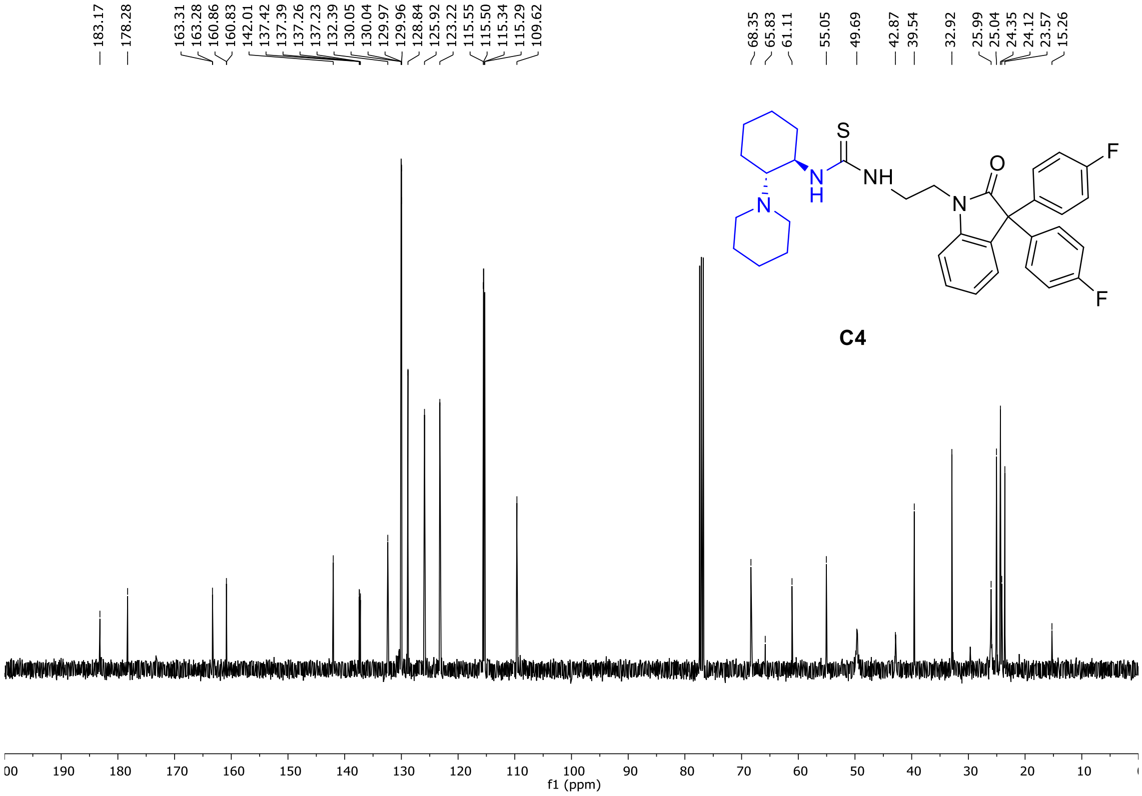

^{1}H NMR (500 MHz, Chloroform-d)/ ^{13}C {^{1}H} NMR (126 MHz, Chloroform-d) of **C5**

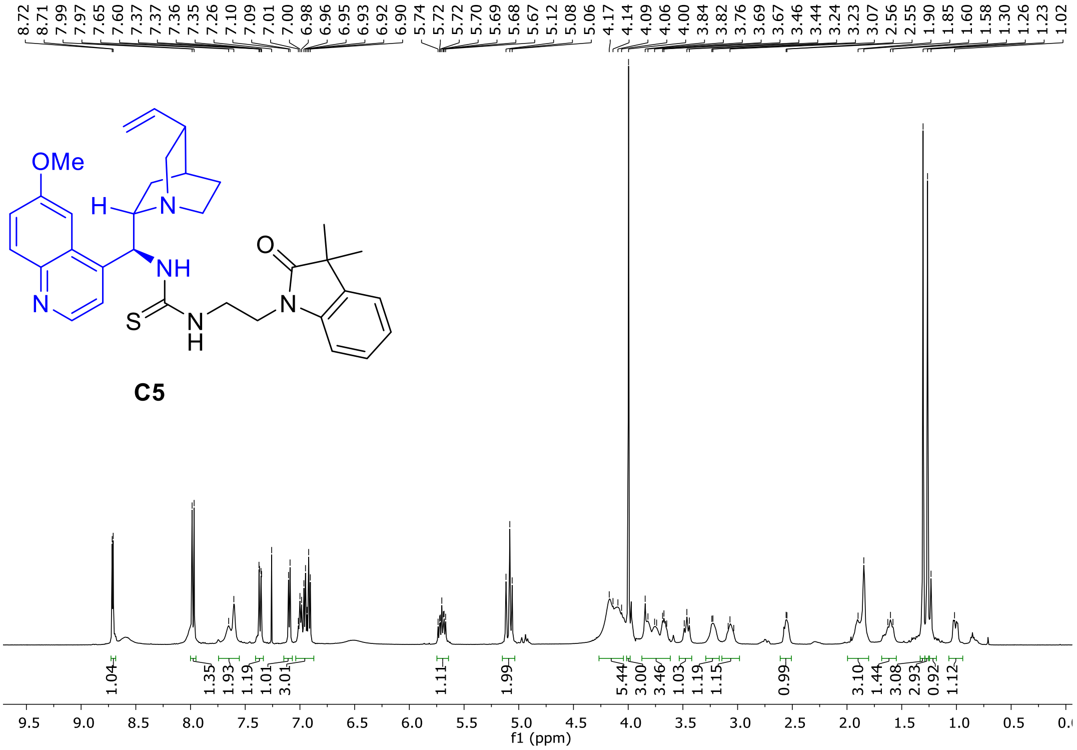

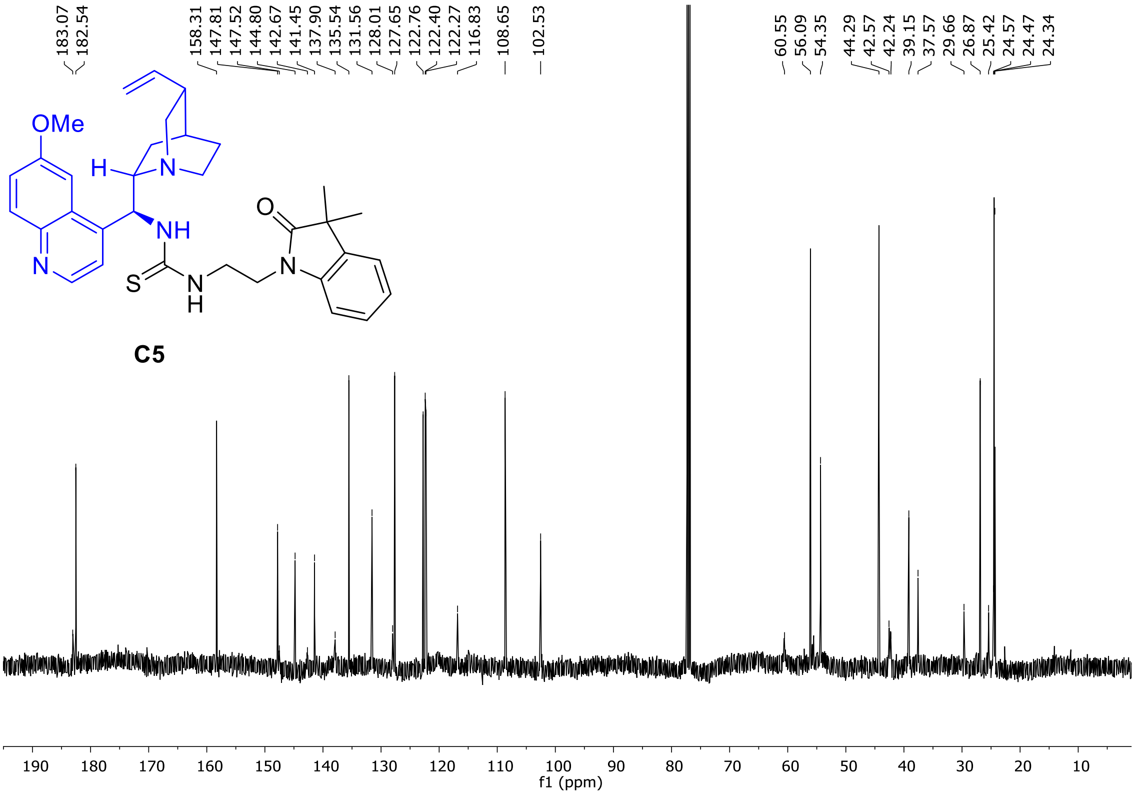

[1] H NMR (400 MHz, DMSO-d^6)/ ^{13}C {[1] H} NMR (101 MHz, DMSO-d^6) of **C6**

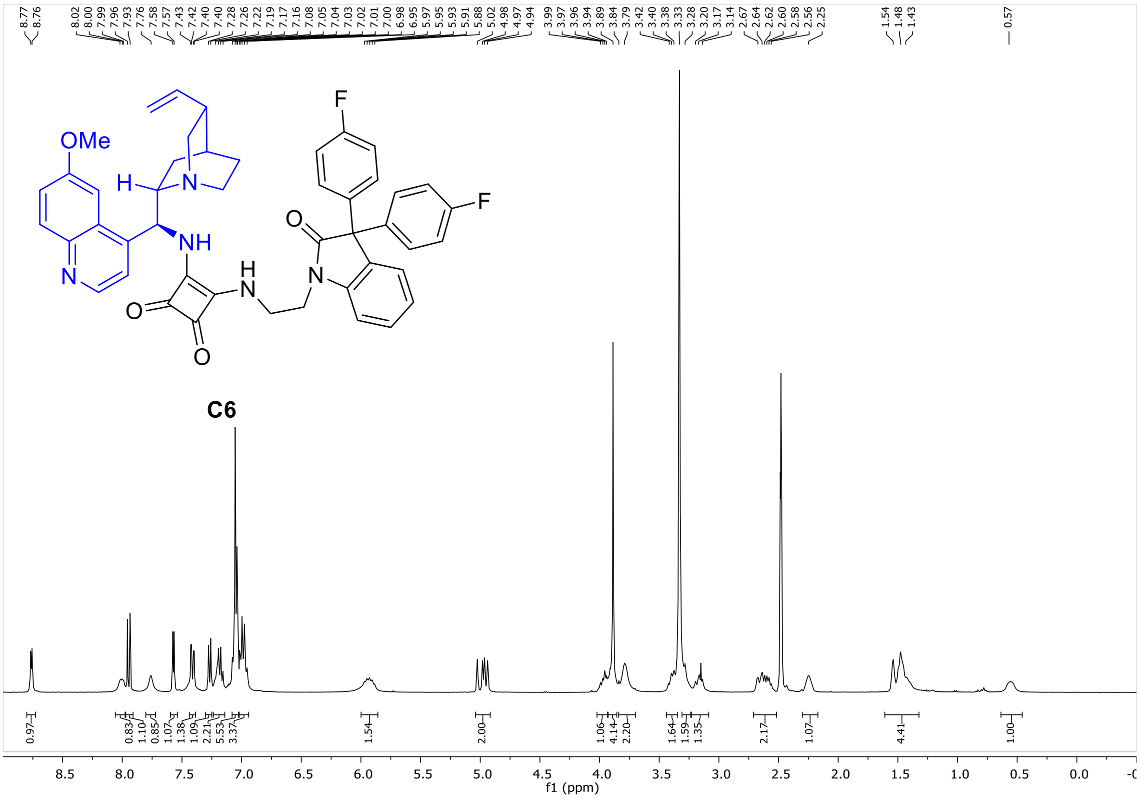

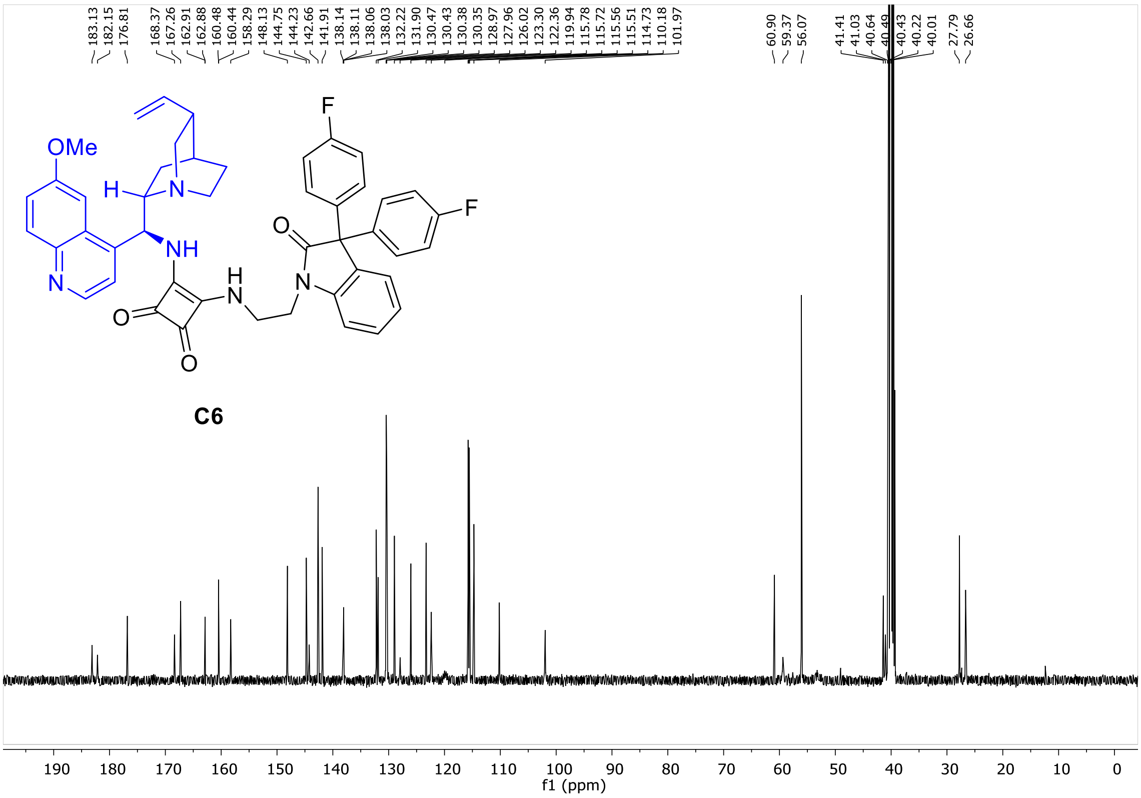

^{1} H NMR (400 MHz, Chloroform-d)/ ^{13}C {^{1} H} NMR (101 MHz, Chloroform-d) of **15b**

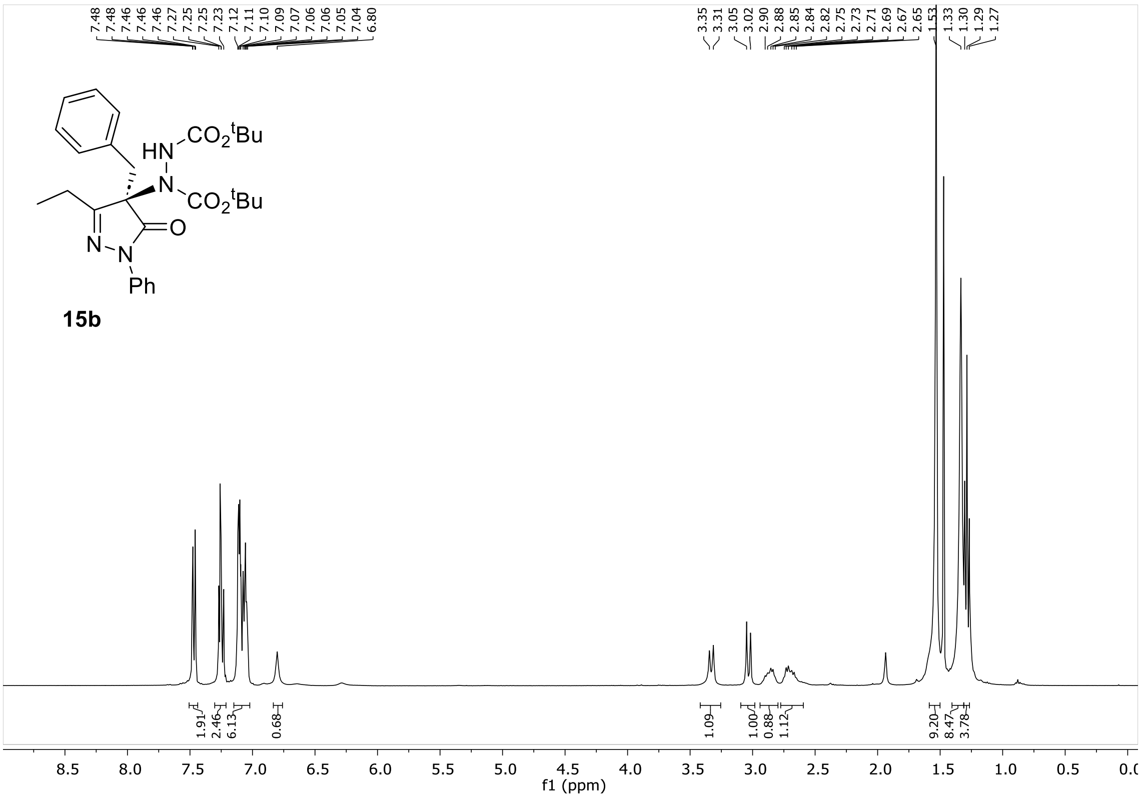

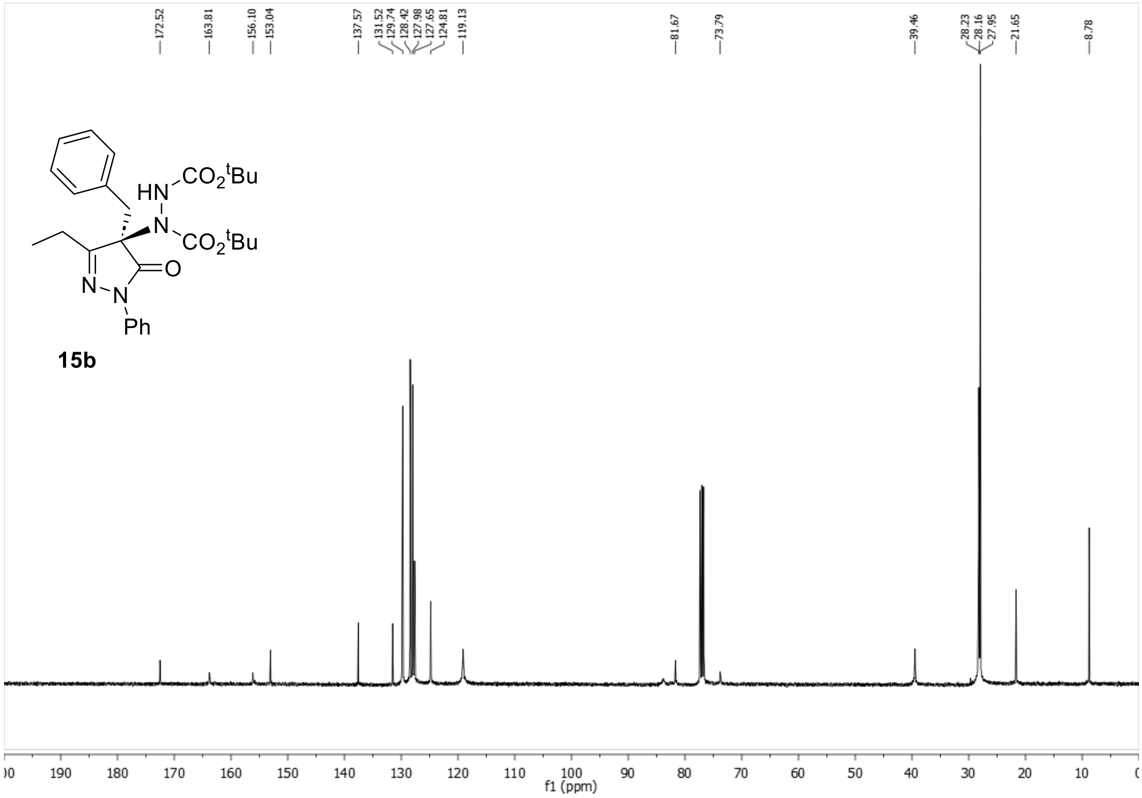

^{1} H NMR (400 MHz, Chloroform-d)/ ^{13}C {^{1} H} NMR (101 MHz, Chloroform-d) of **15c**

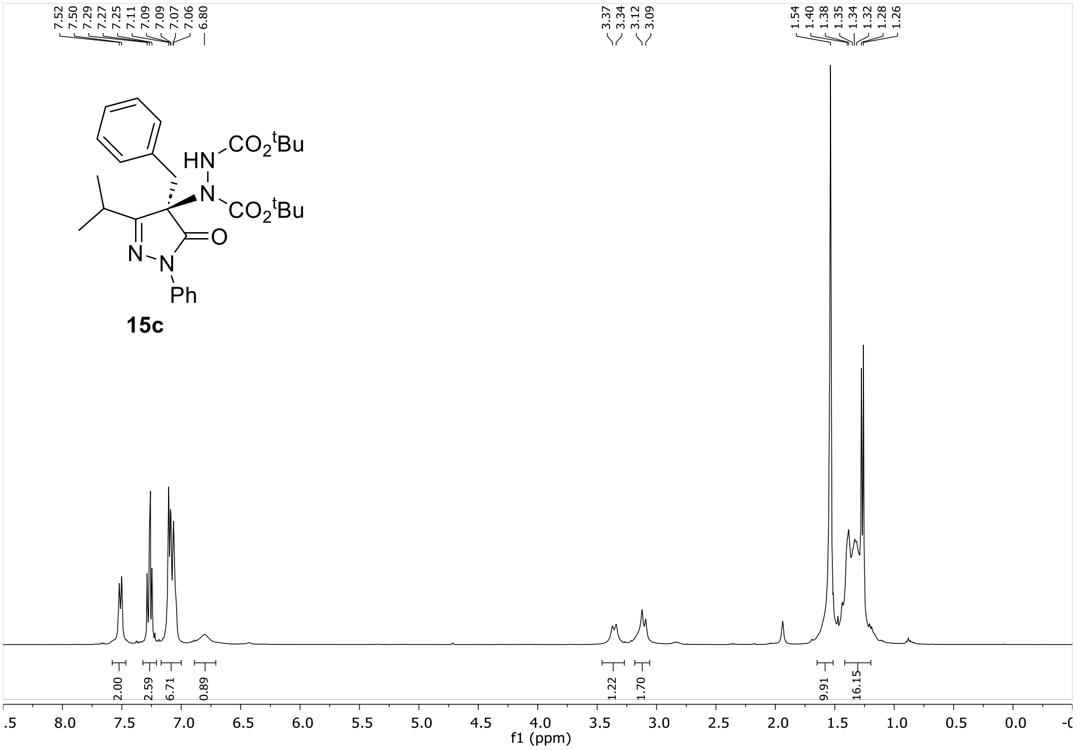

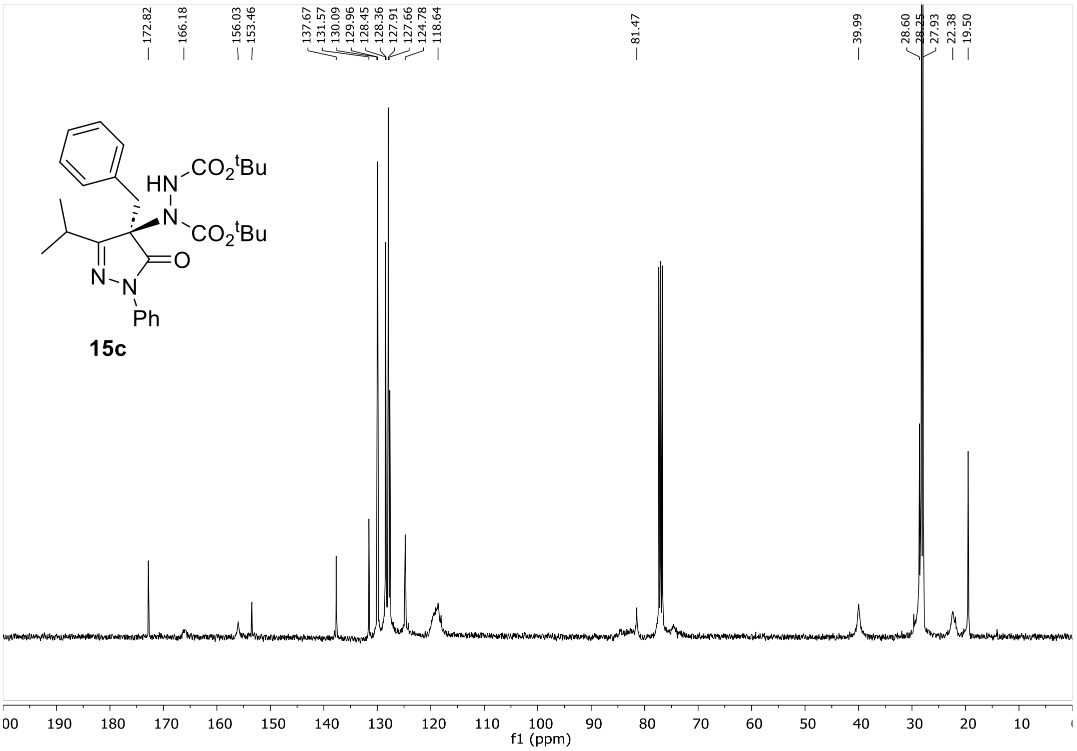

[1] H NMR (400 MHz, Chloroform-d)/ ^{13}C {[1] H} NMR (101 MHz, Chloroform-d) of **15d**

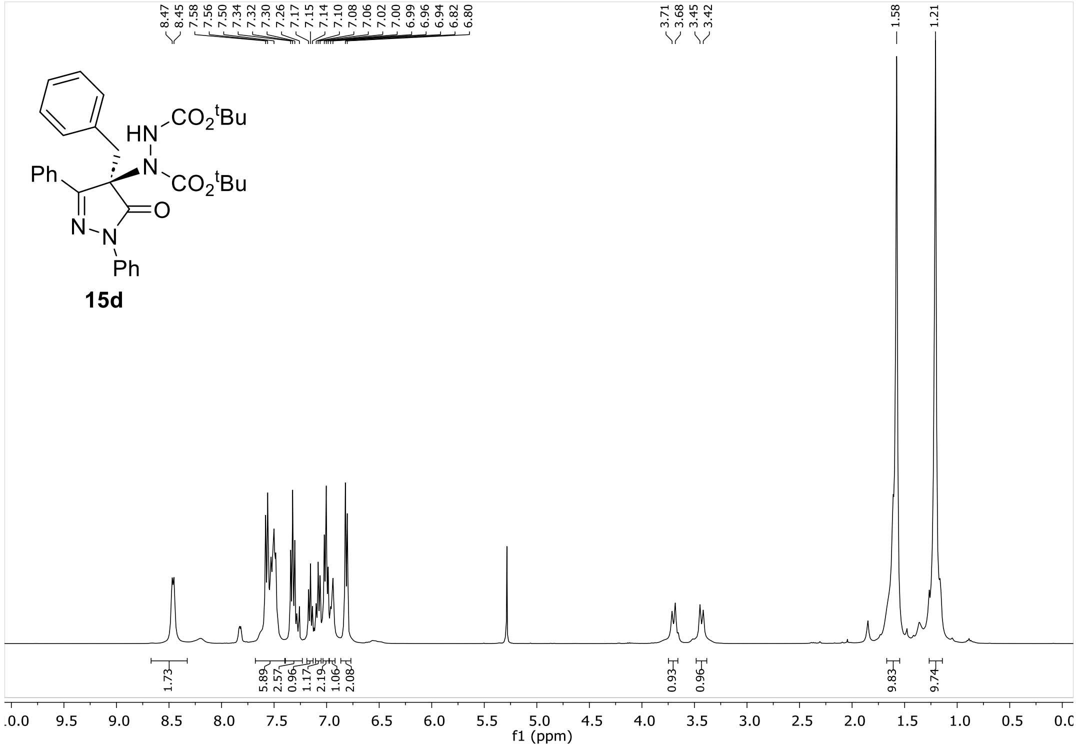

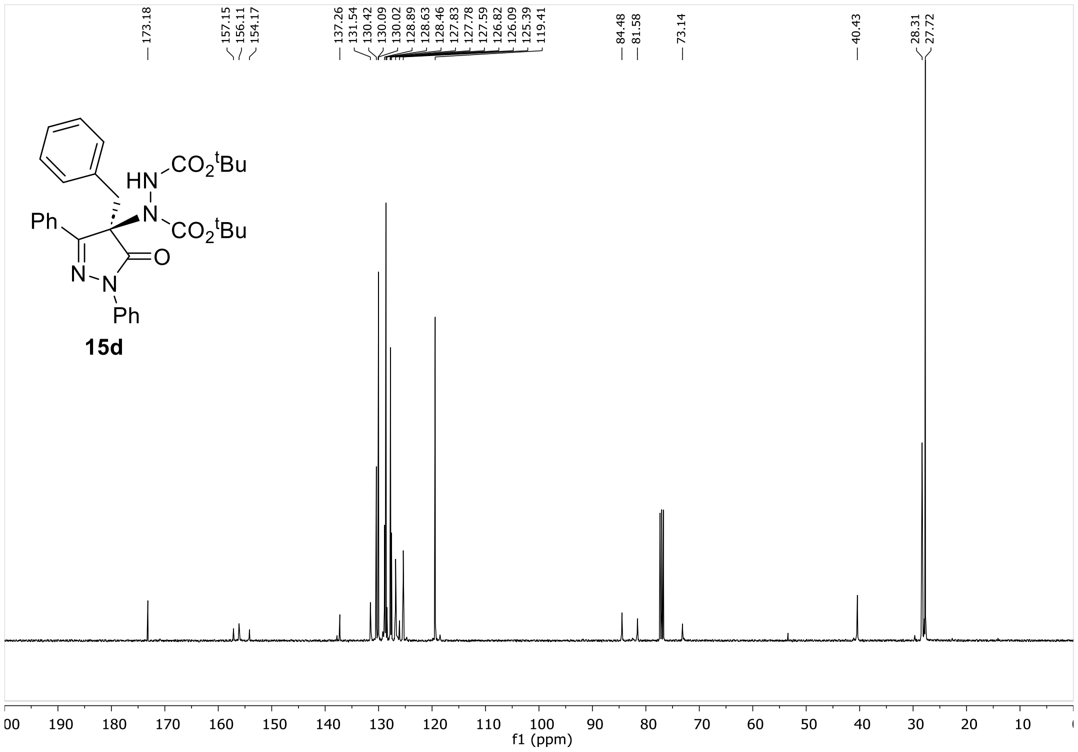

^{1}H NMR (400 MHz, Chloroform-d)/ $^{13}C\{^{1}H\}$ NMR (101 MHz, Chloroform-d) of **15e**

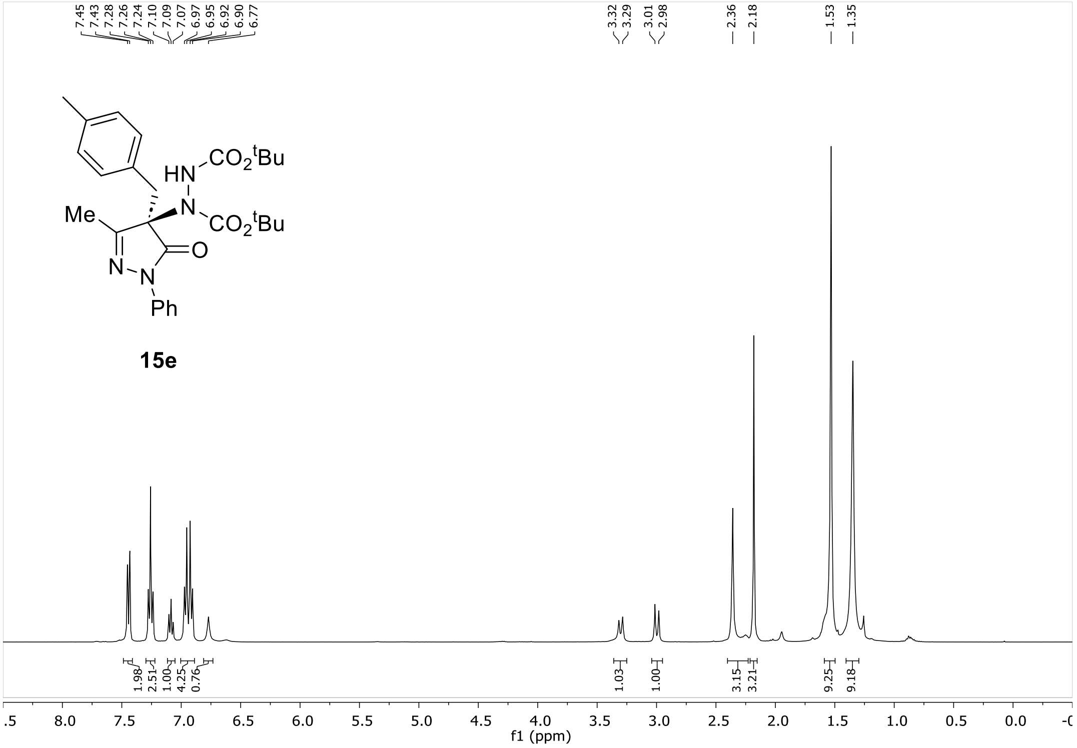

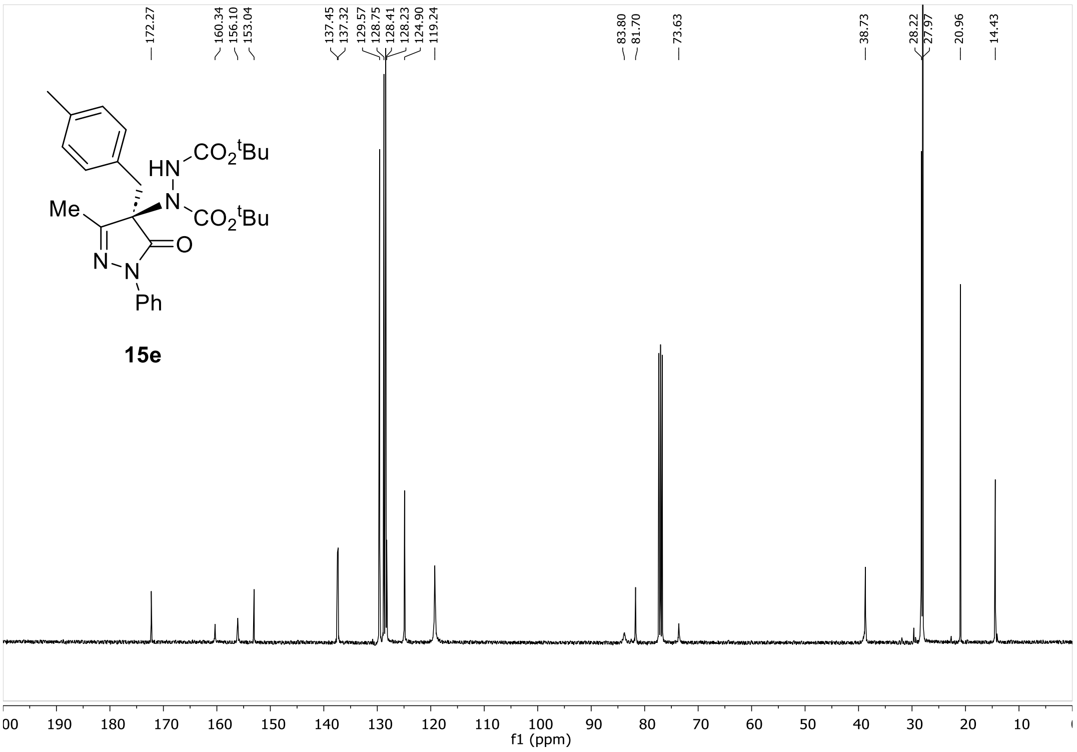

^{1} H NMR (400 MHz, Chloroform-d)/ ^{13}C {^{1} H} NMR (101 MHz, Chloroform-d) of **15f**

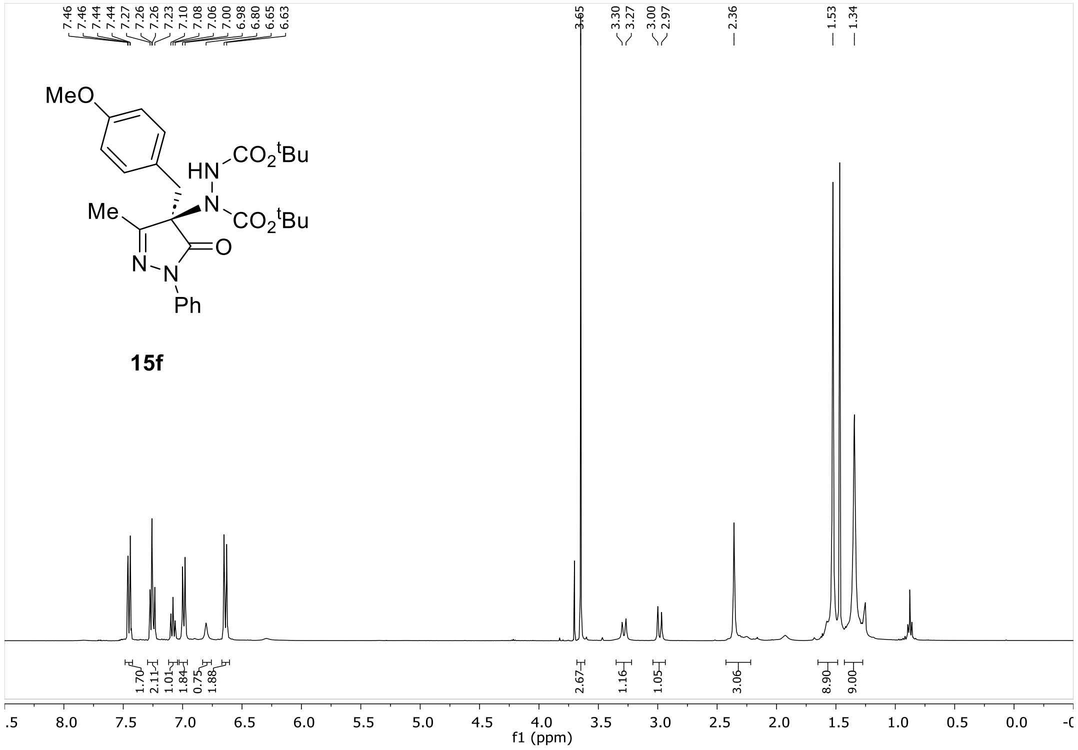

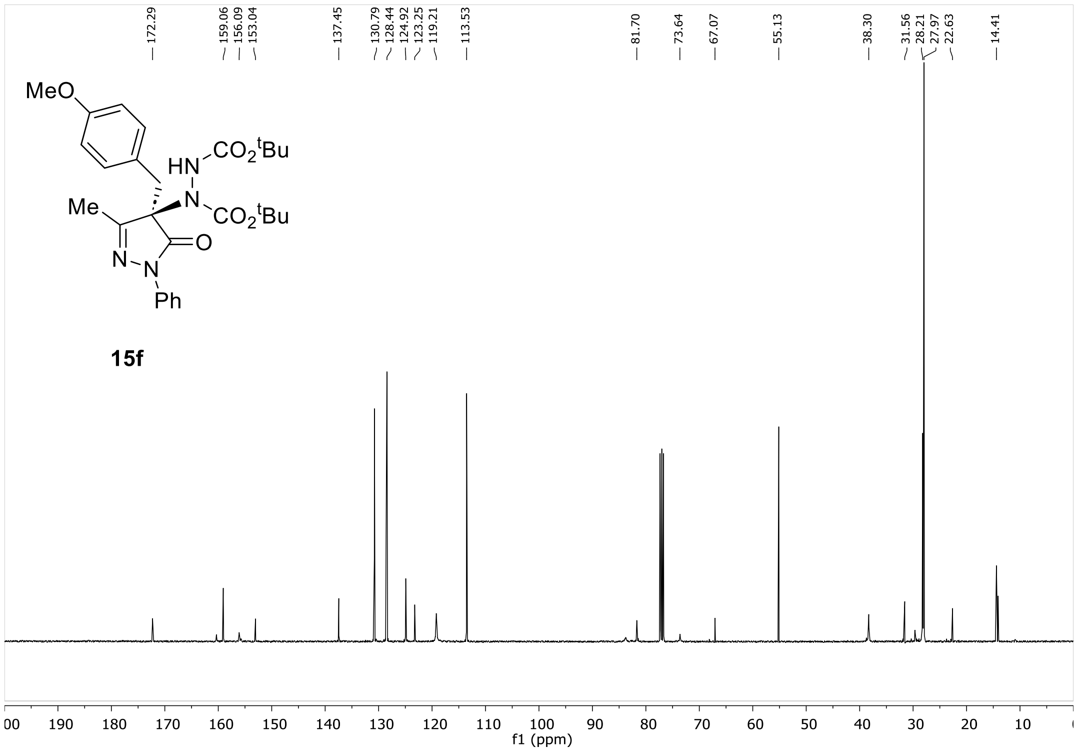

^{1}H NMR (400 MHz, Chloroform-d)/ ^{13}C {^{1}H} NMR (101 MHz, Chloroform-d) of **15g**

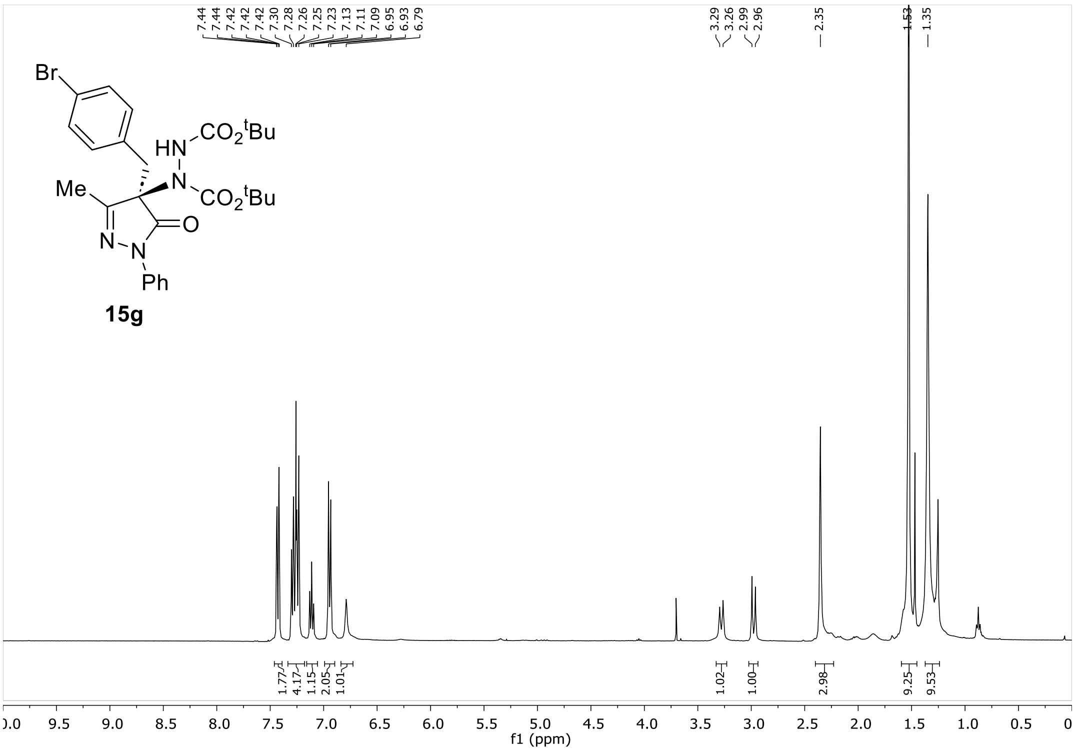

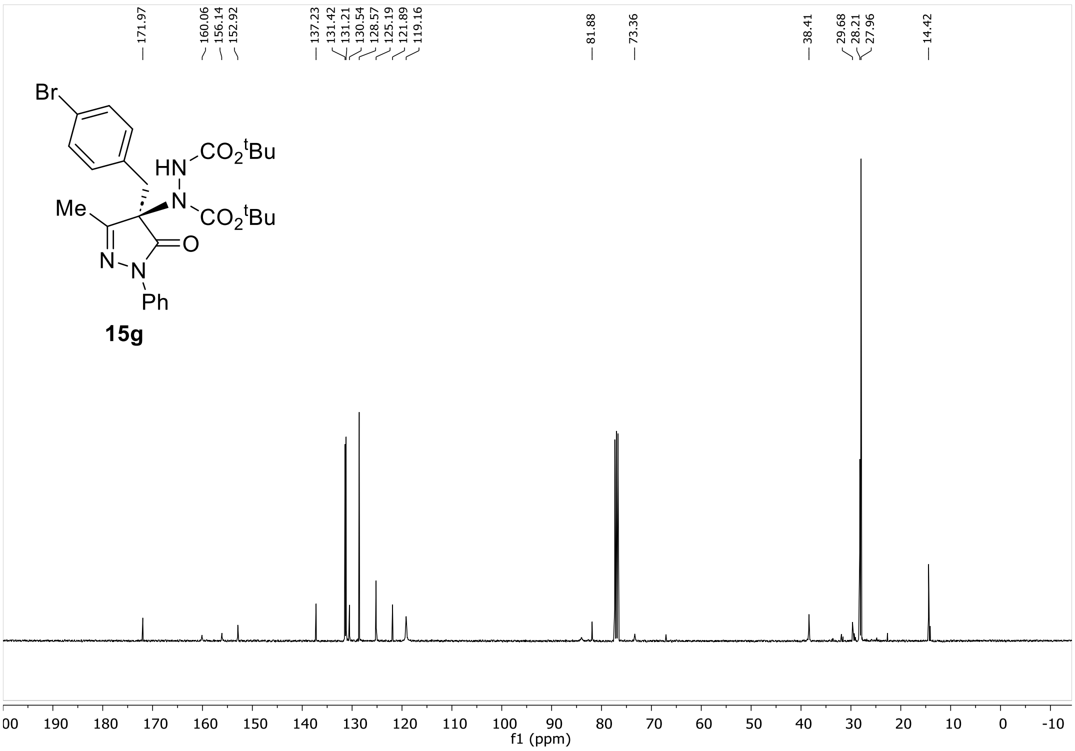

[1] H NMR (400 MHz, Chloroform-d)/ ^{13}C {[1] H} NMR (101 MHz, Chloroform-d) of **15h**

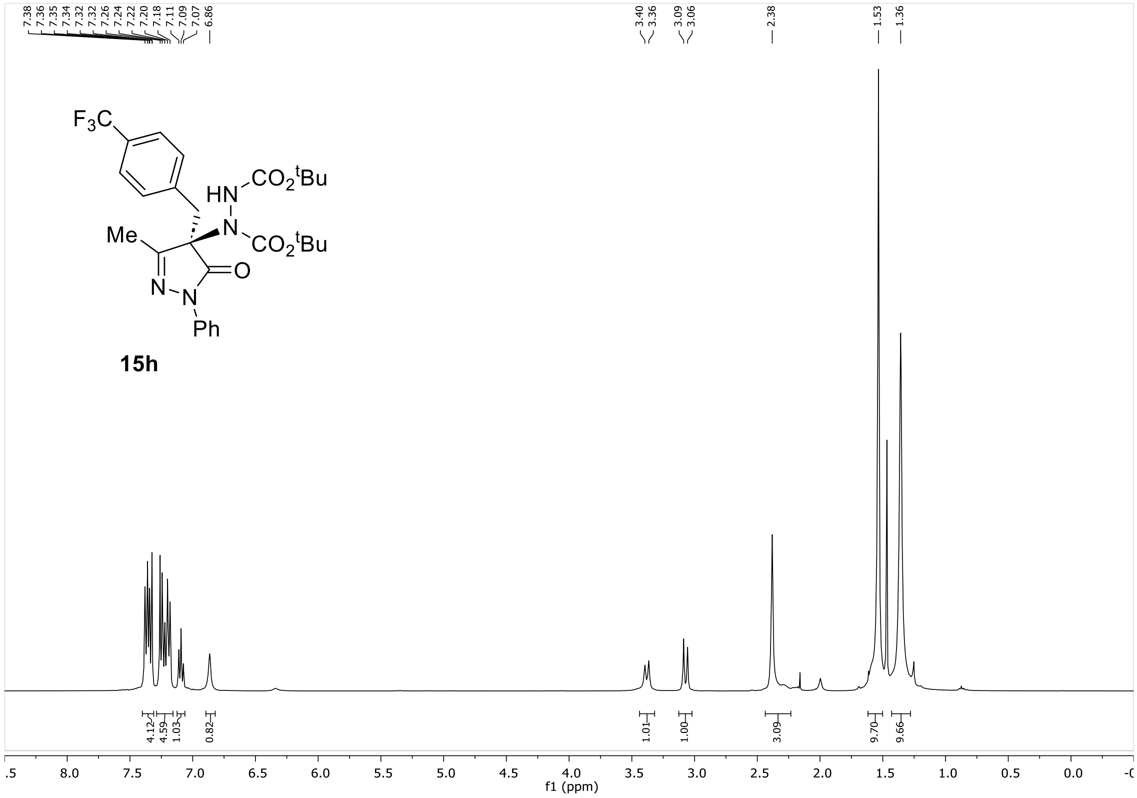

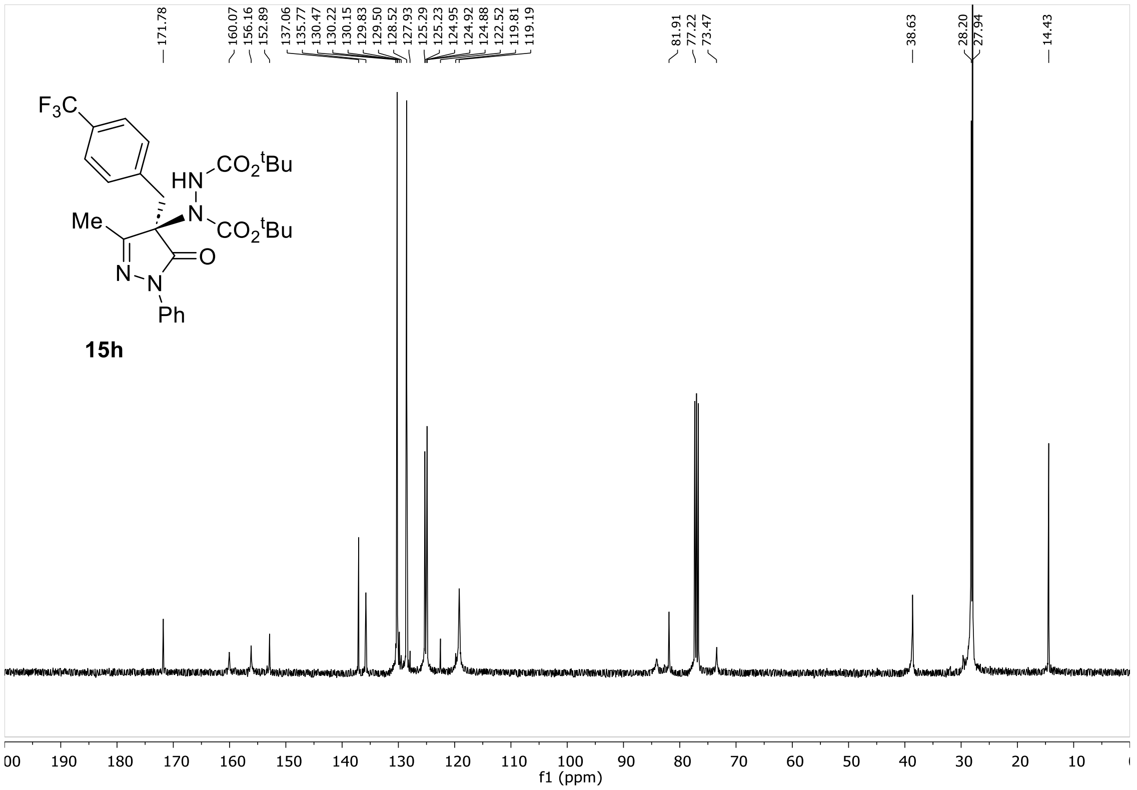

^{1} H NMR (400 MHz, Chloroform-d)/ ^{13}C {^{1} H} NMR (101 MHz, Chloroform-d) of **15i**

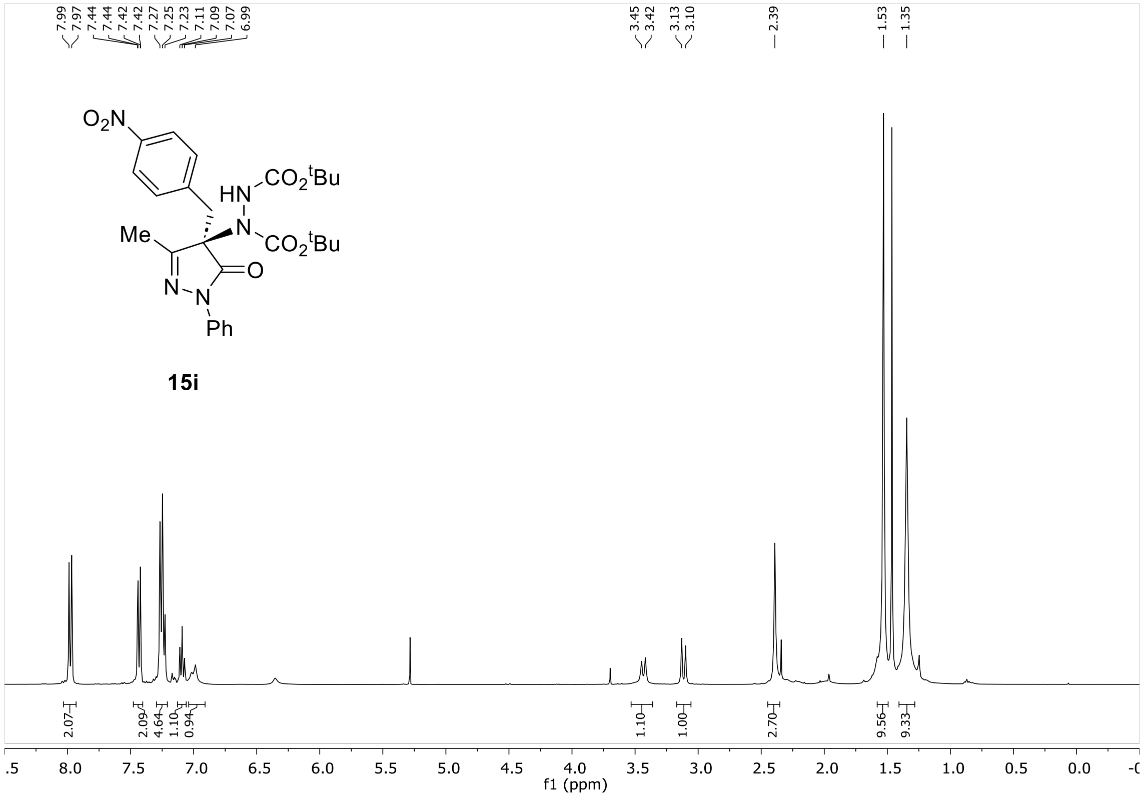

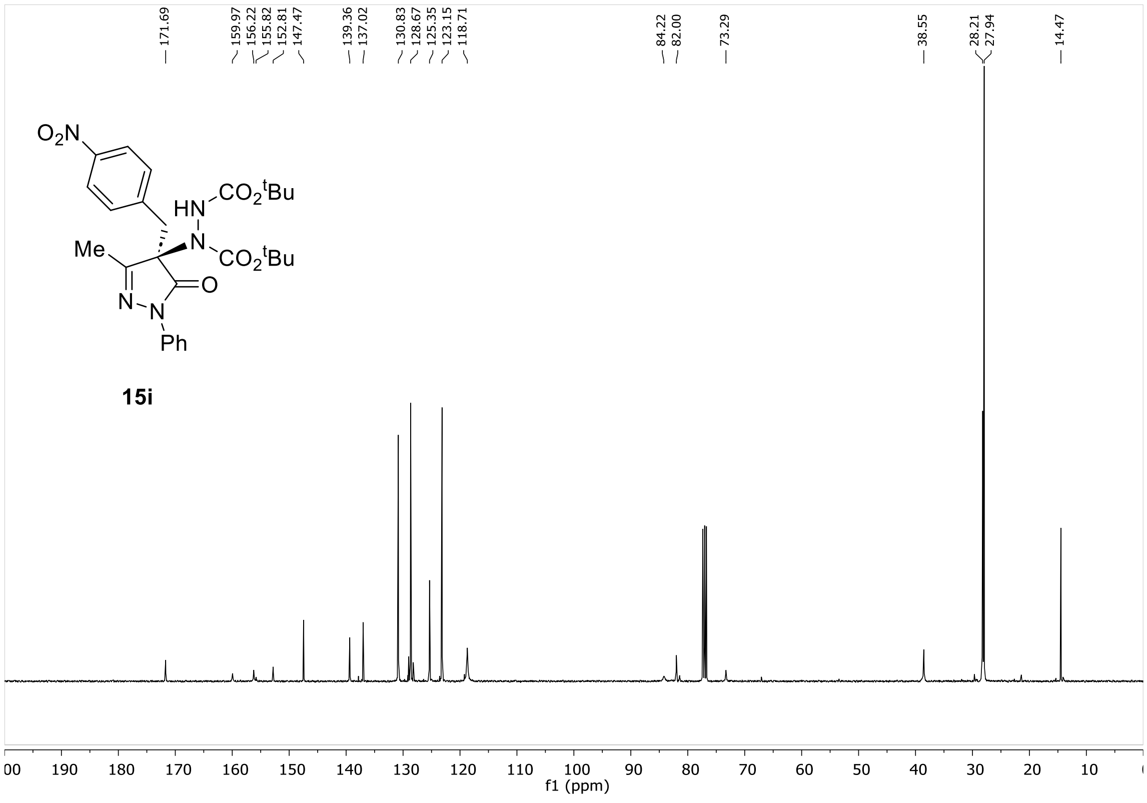

[1] H NMR (400 MHz, Chloroform-d)/ ^{13}C {[1] H} NMR (101 MHz, Chloroform-d) of **15j**

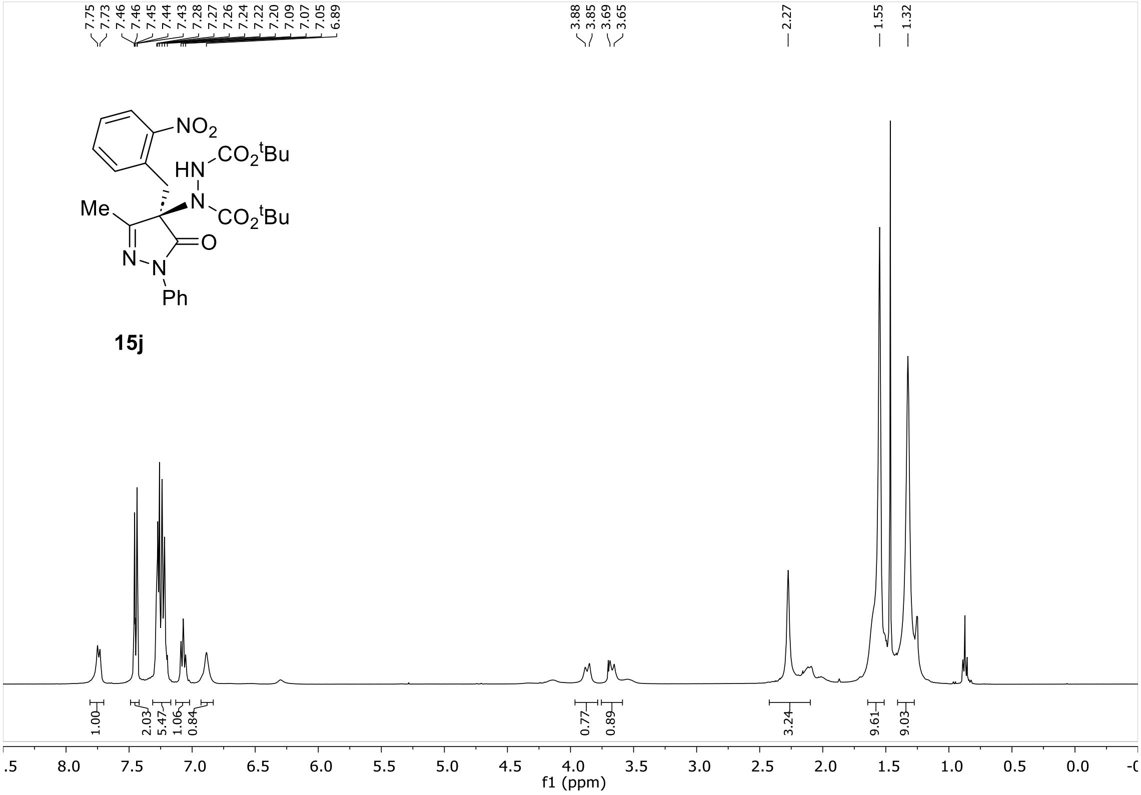

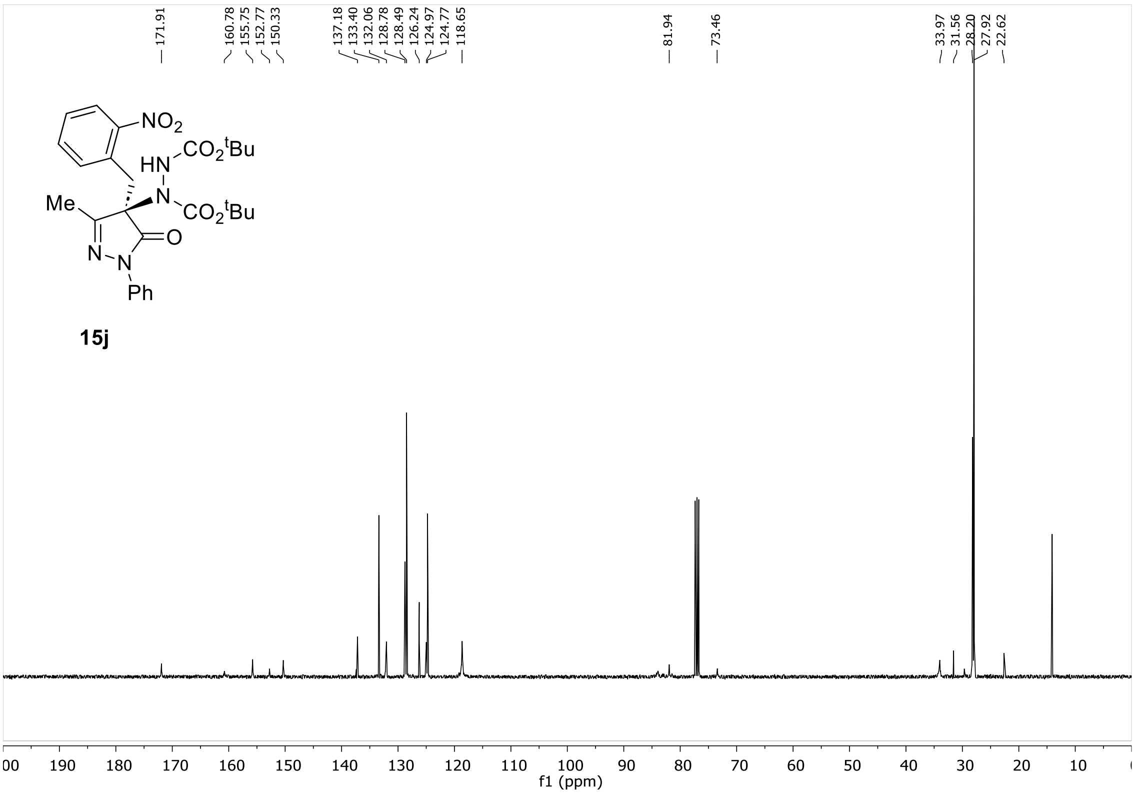

[1] H NMR (400 MHz, Chloroform-d)/ ^{13}C {[1] H} NMR (101 MHz, Chloroform-d) of **15k**

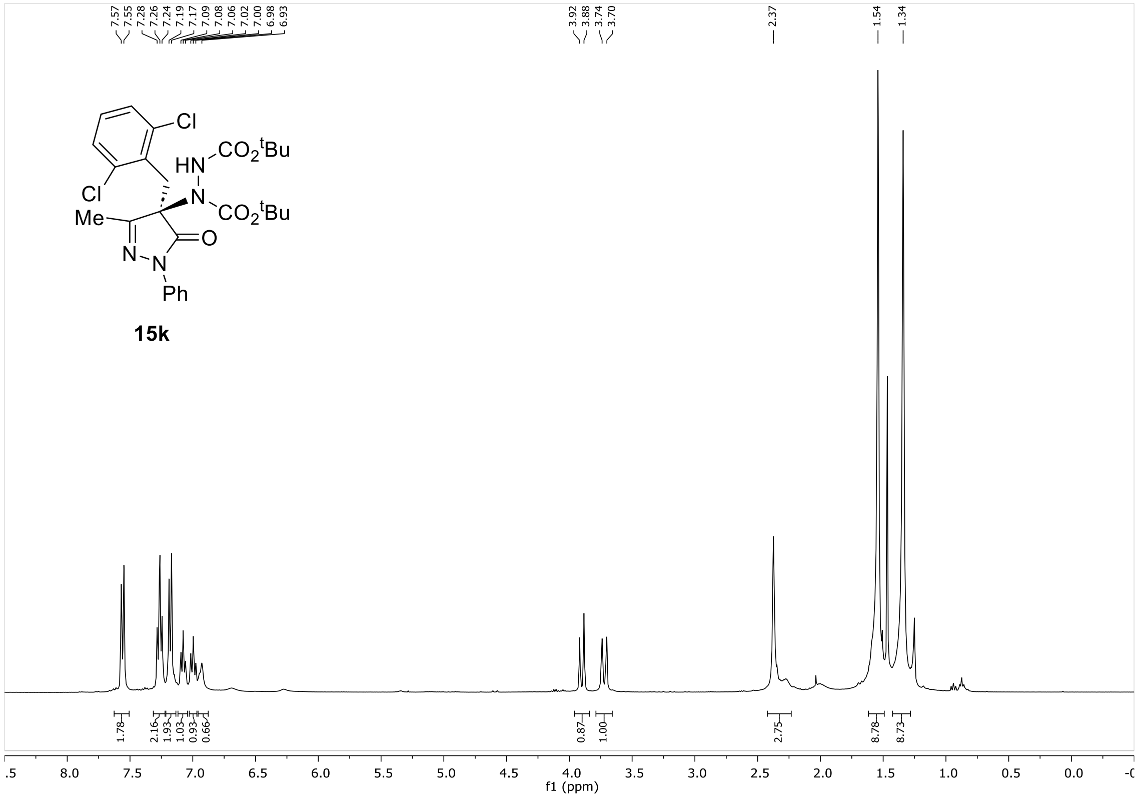

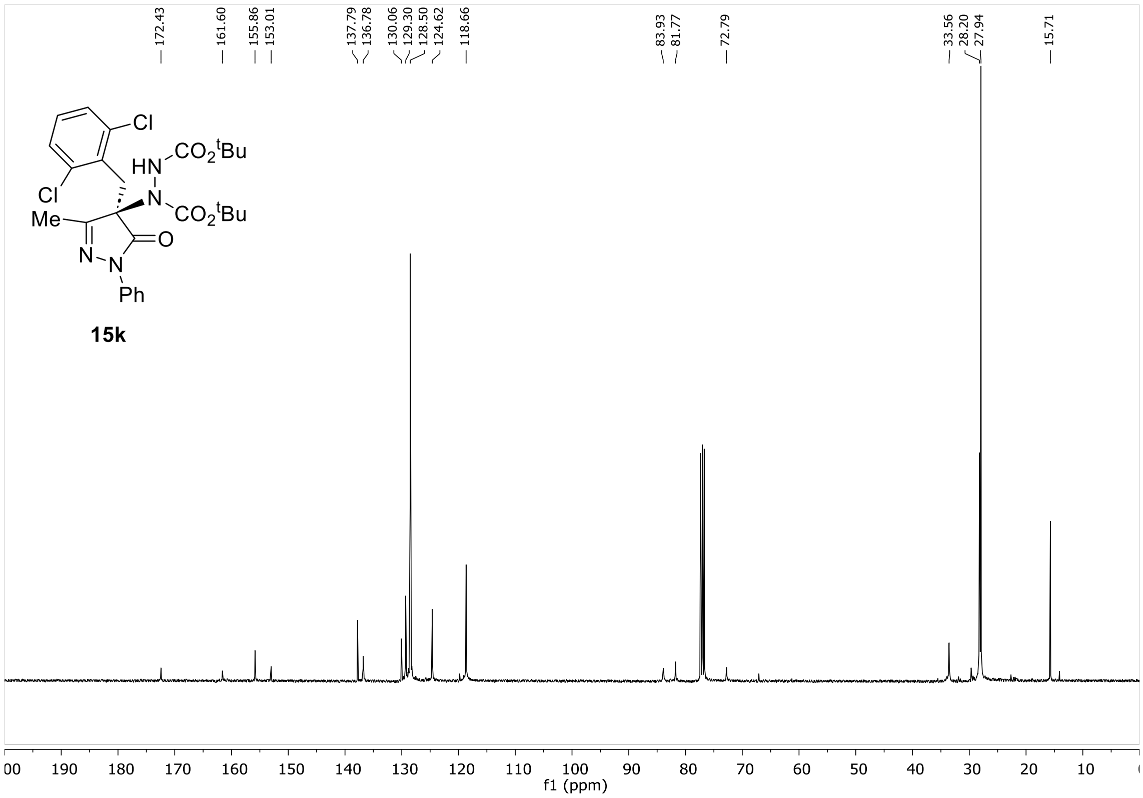

^{1}H NMR (400 MHz, Chloroform-d)/ ^{13}C {^{1}H} NMR (101 MHz, Chloroform-d) of **15l**

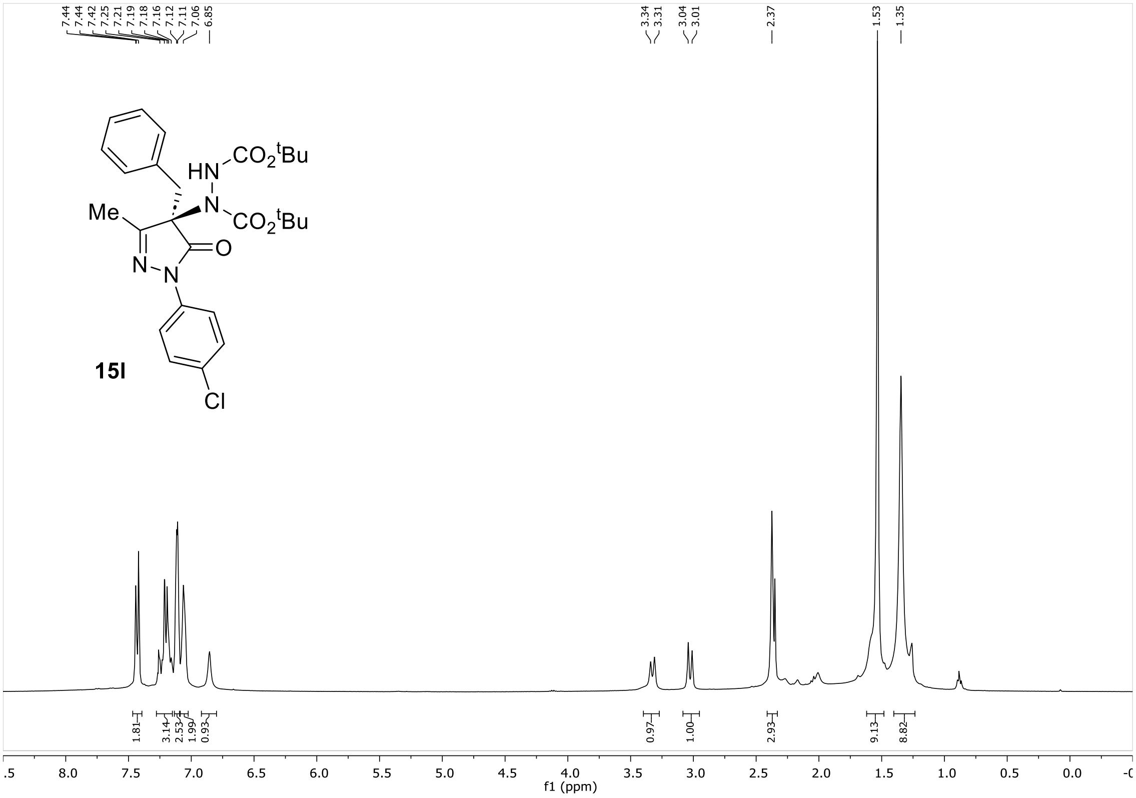

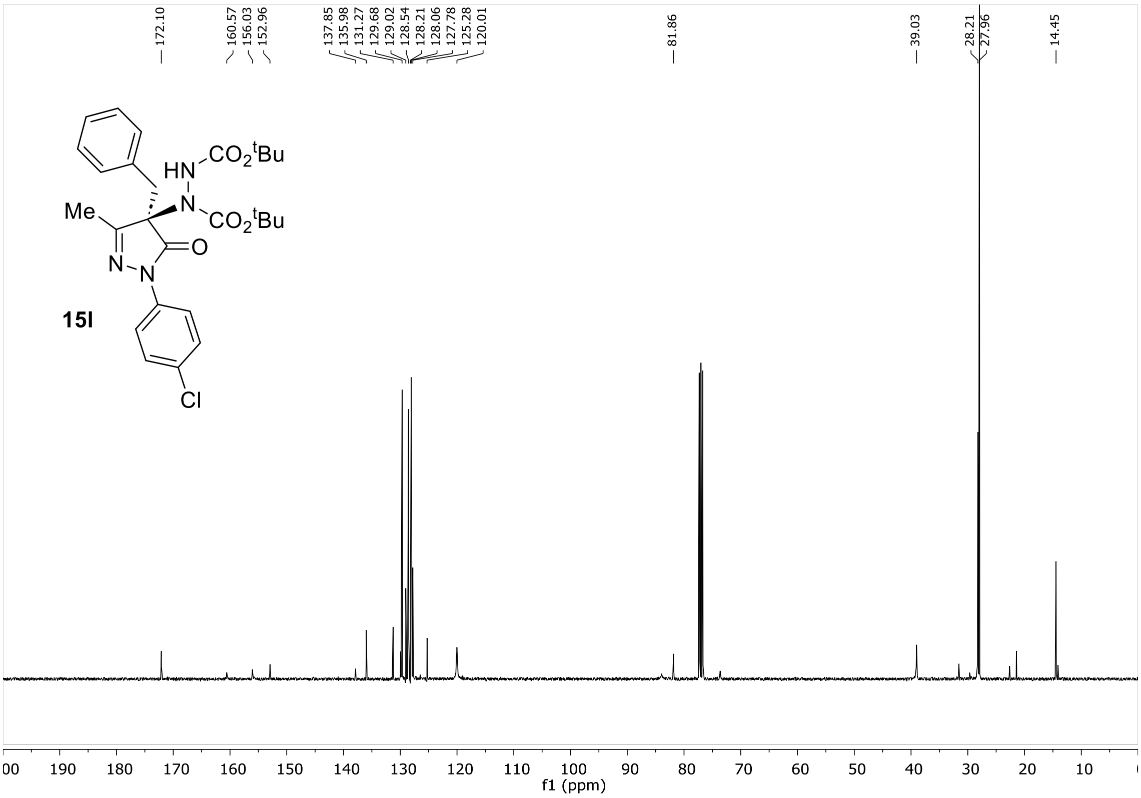

[1] H NMR (500 MHz, Chloroform-d)/ ^{13}C {[1] H} NMR (126 MHz, Chloroform-d) of **15m**

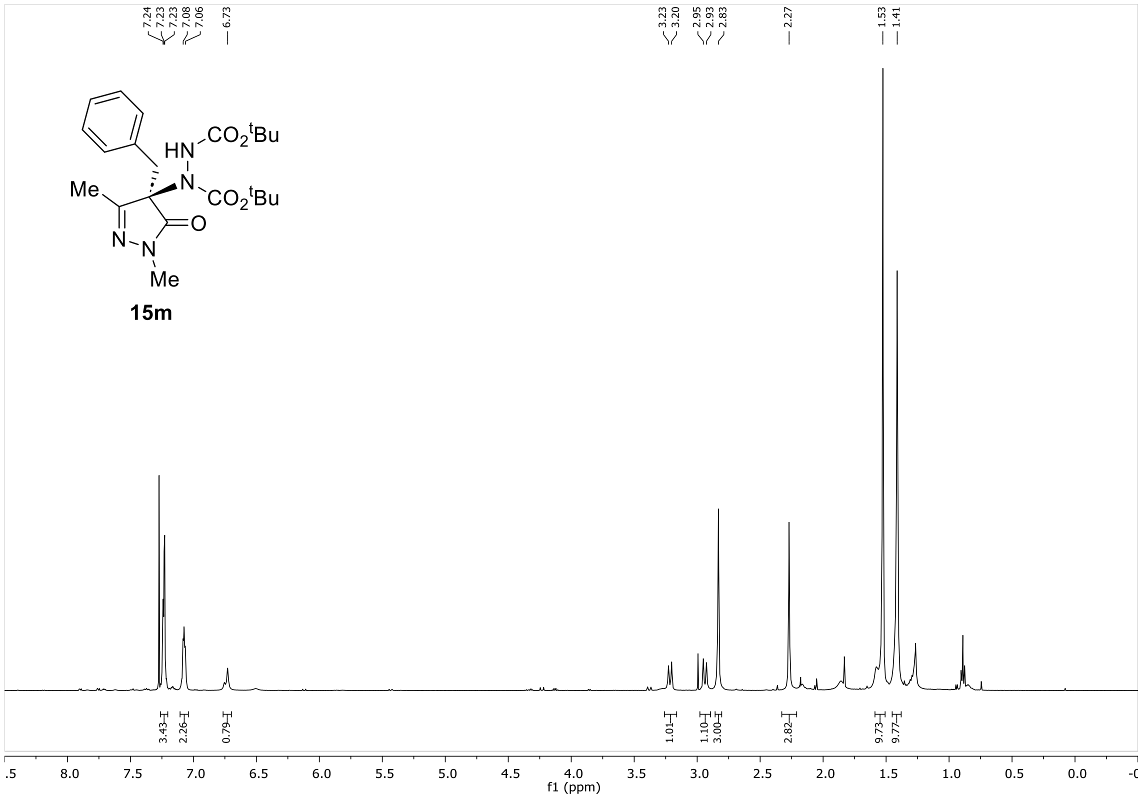

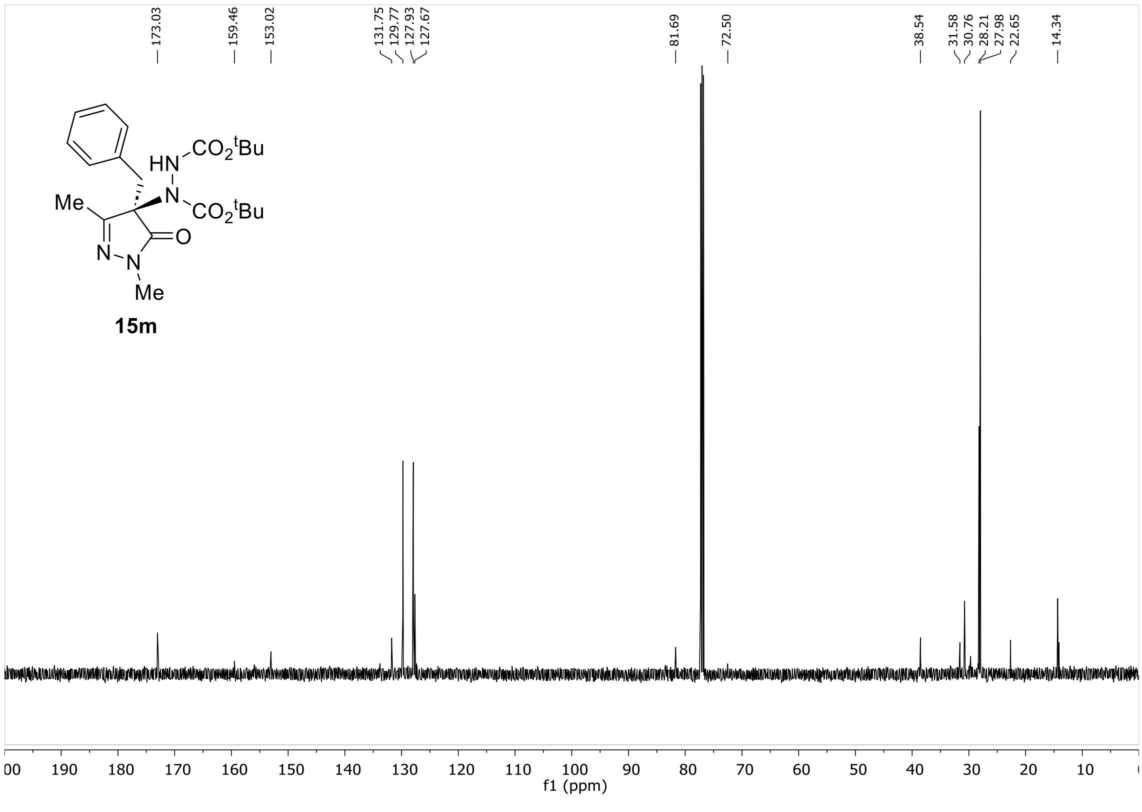

^{1} H NMR (400 MHz, Chloroform-d)/ ^{13}C {^{1} H} NMR (101 MHz, Chloroform-d) of **15n**

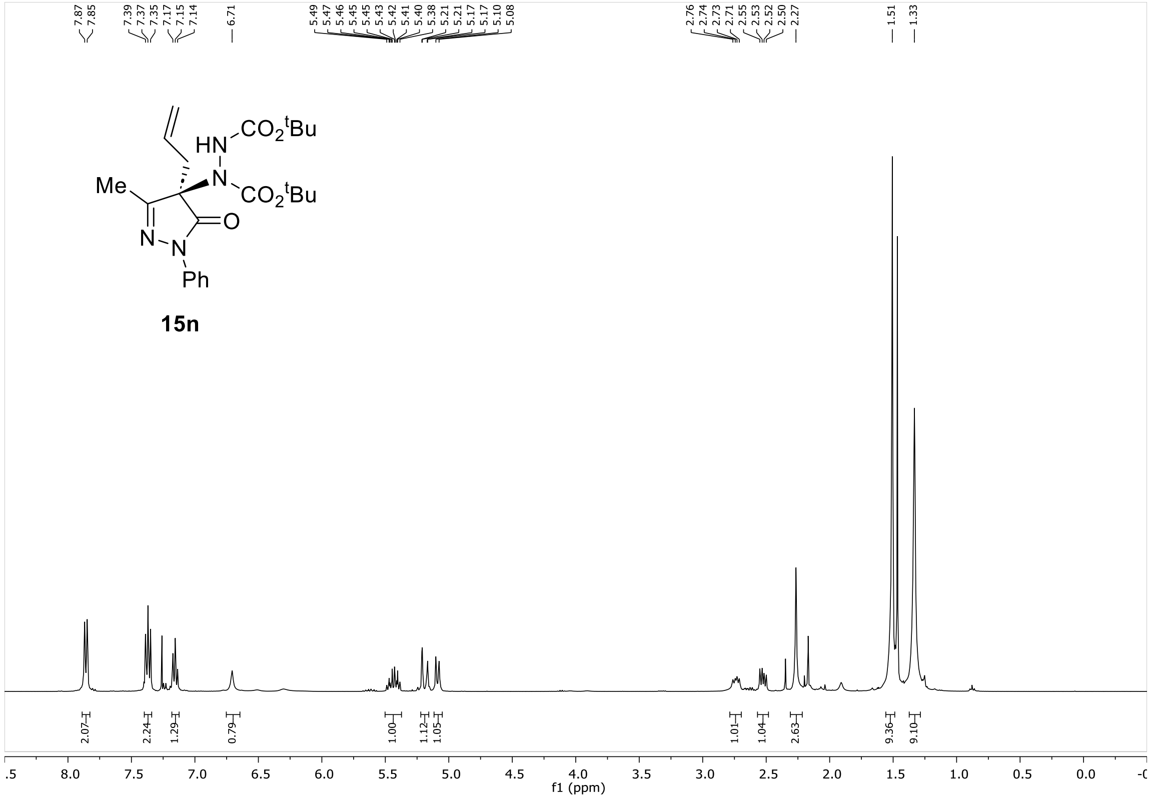

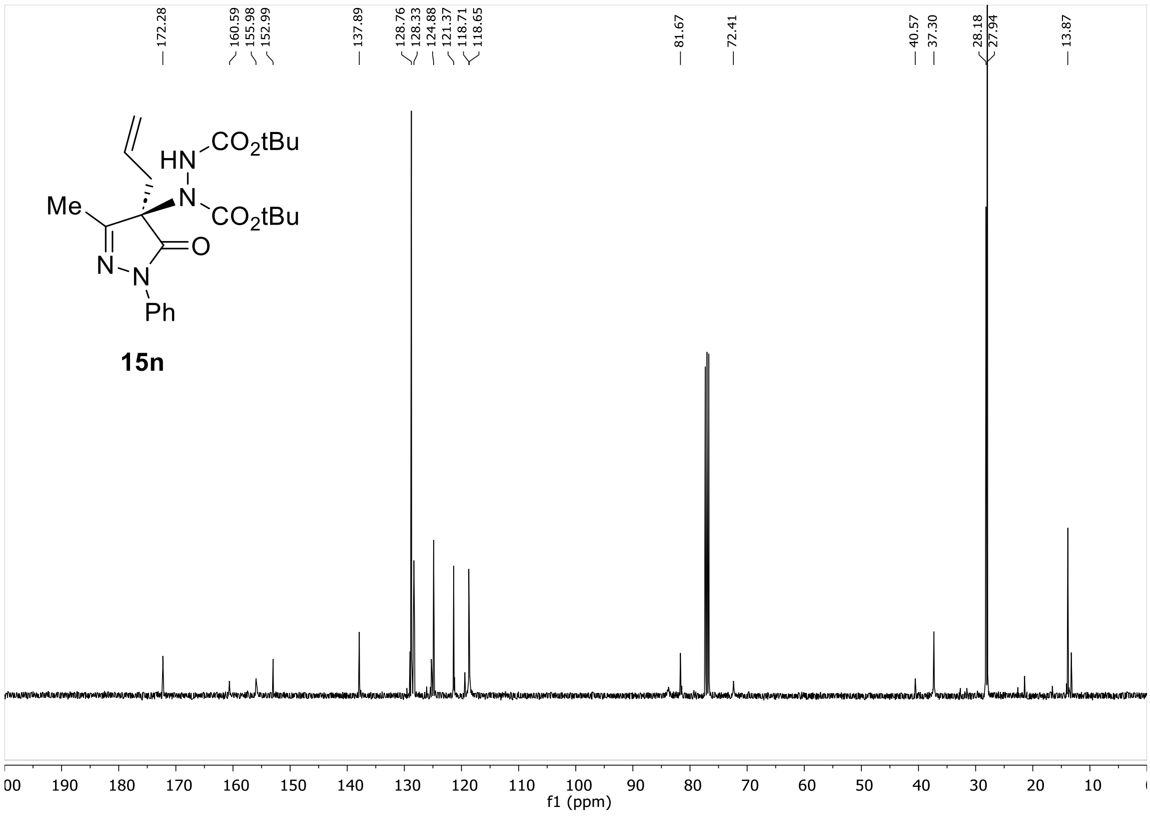

^{1} H NMR (400 MHz, Chloroform-d)/ ^{13}C {^{1} H} NMR (101 MHz, Chloroform-d) of **15o**

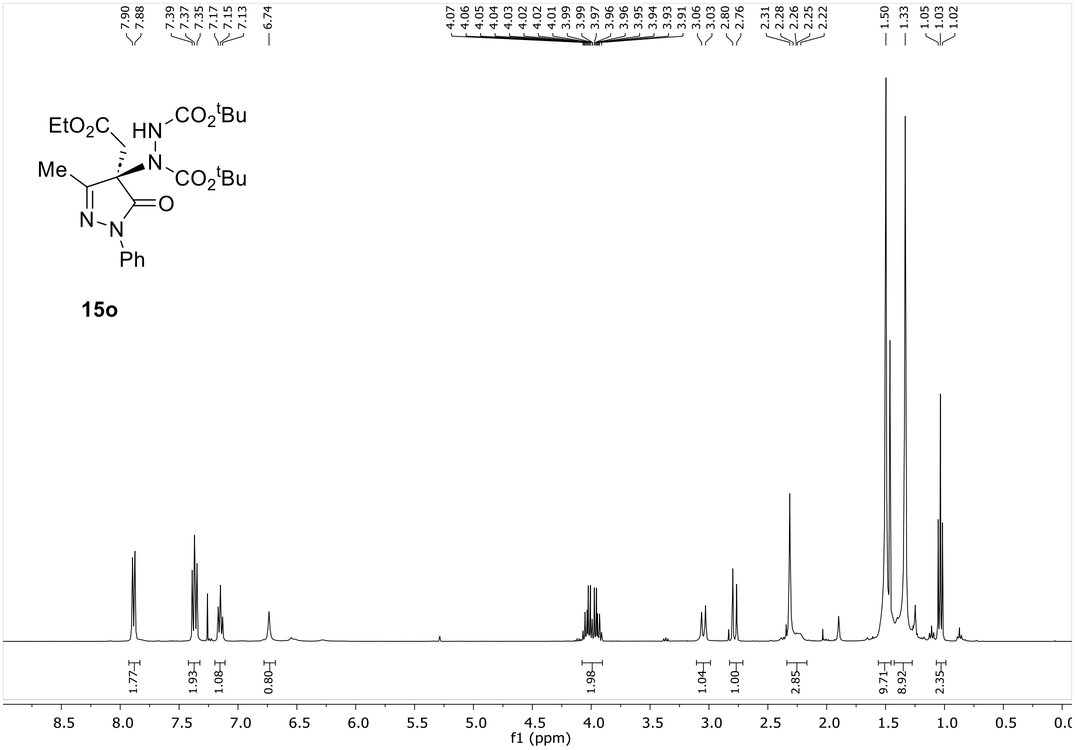

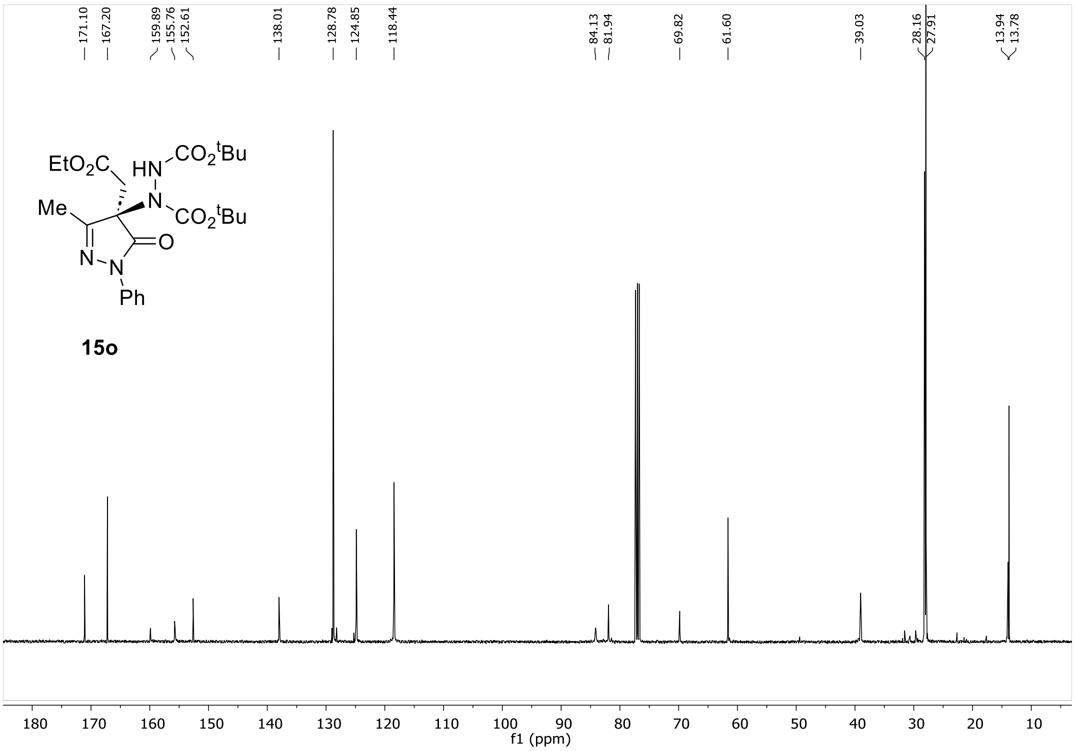

[1] H NMR (400 MHz, Chloroform-d)/ ^{13}C {[1] H} NMR (101 MHz, Chloroform-d) of **16**

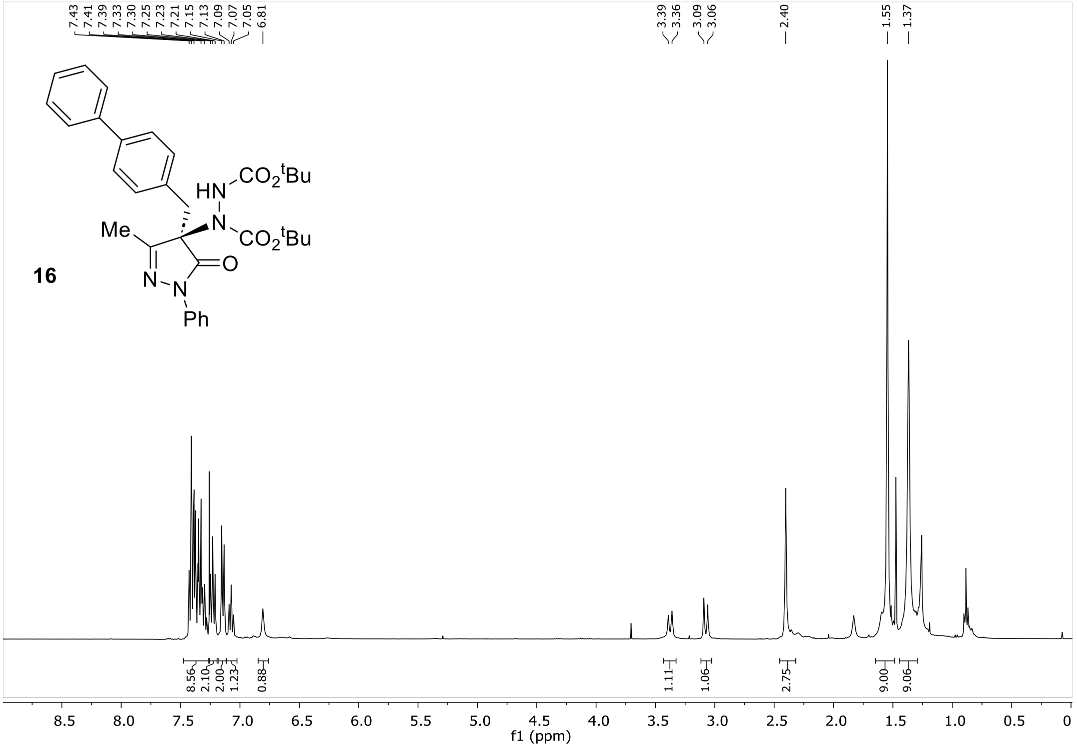

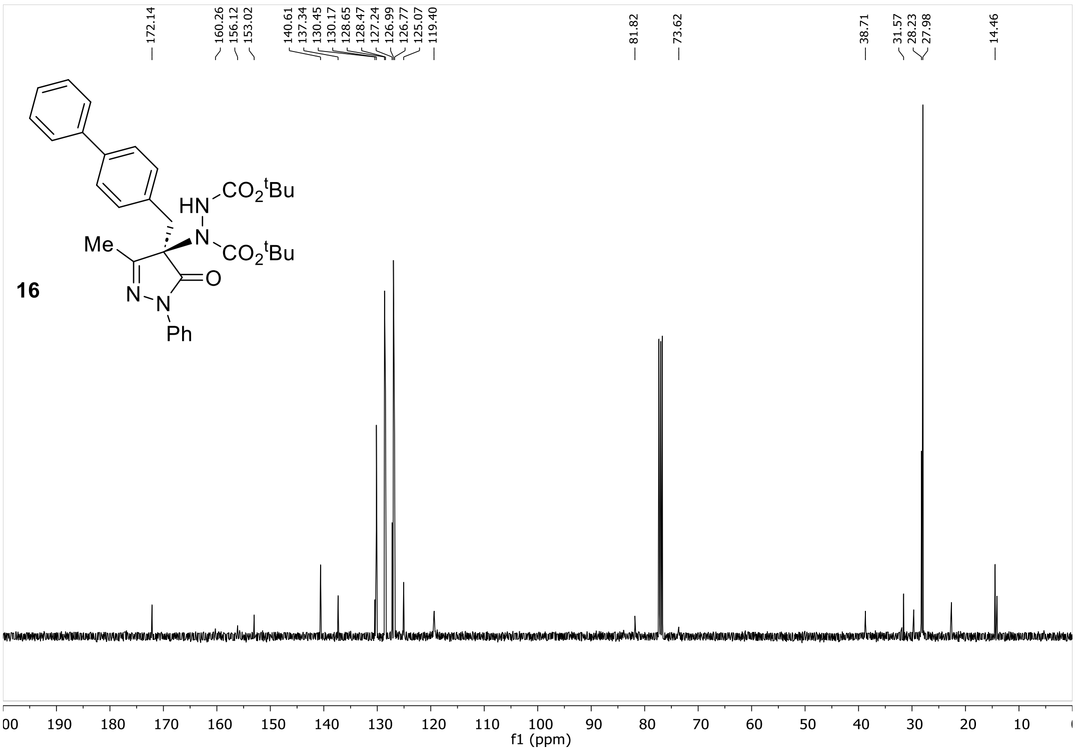

[1] H NMR (400 MHz, DMSO-d[6])/ ^{13}C {[1] H} NMR (101 MHz, DMSO-d[6]) of **LP-I**

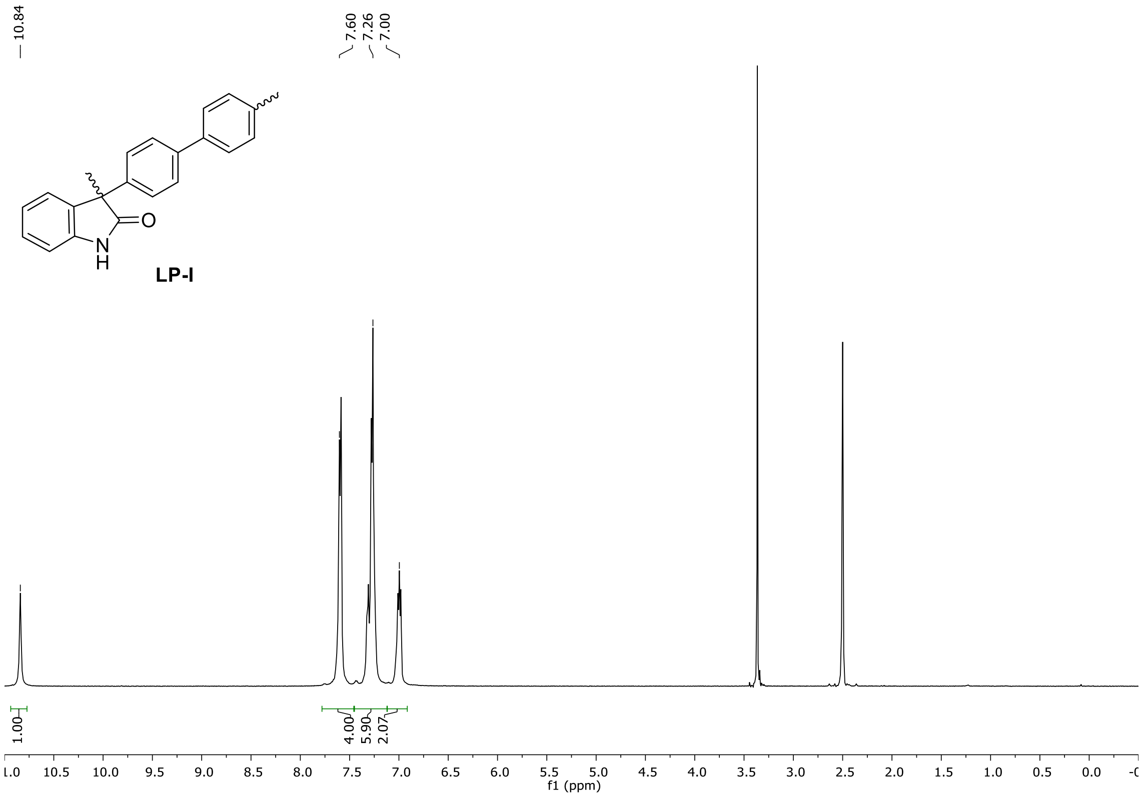

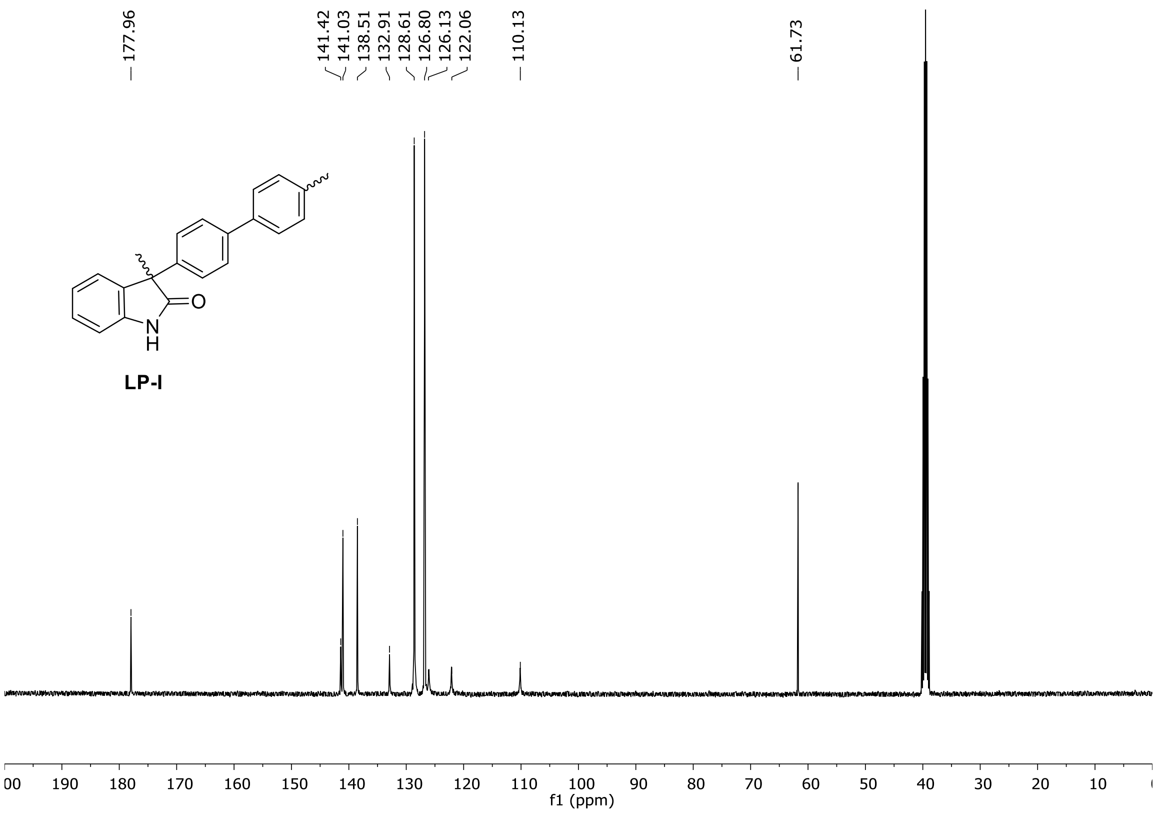

[1] H NMR (400 MHz, DMSO-d^6)/ ^{13}C {[1] H} NMR (101 MHz, DMSO-d^6) of **LP-II**

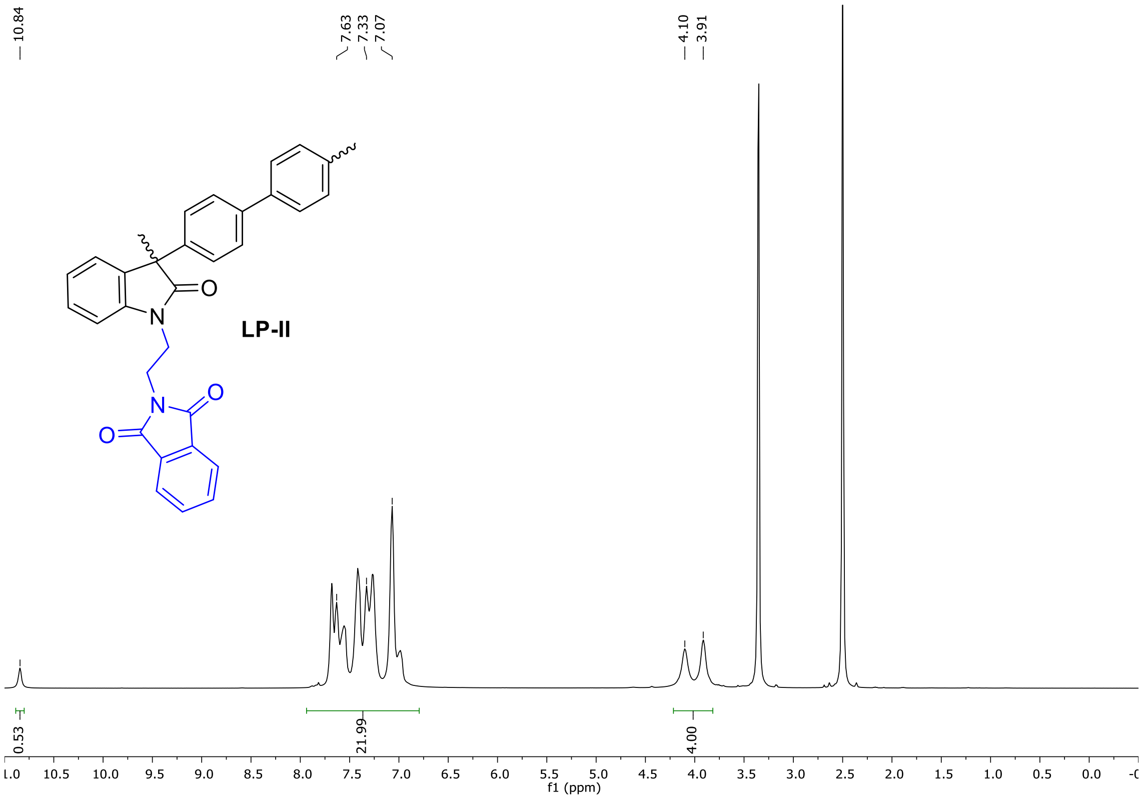

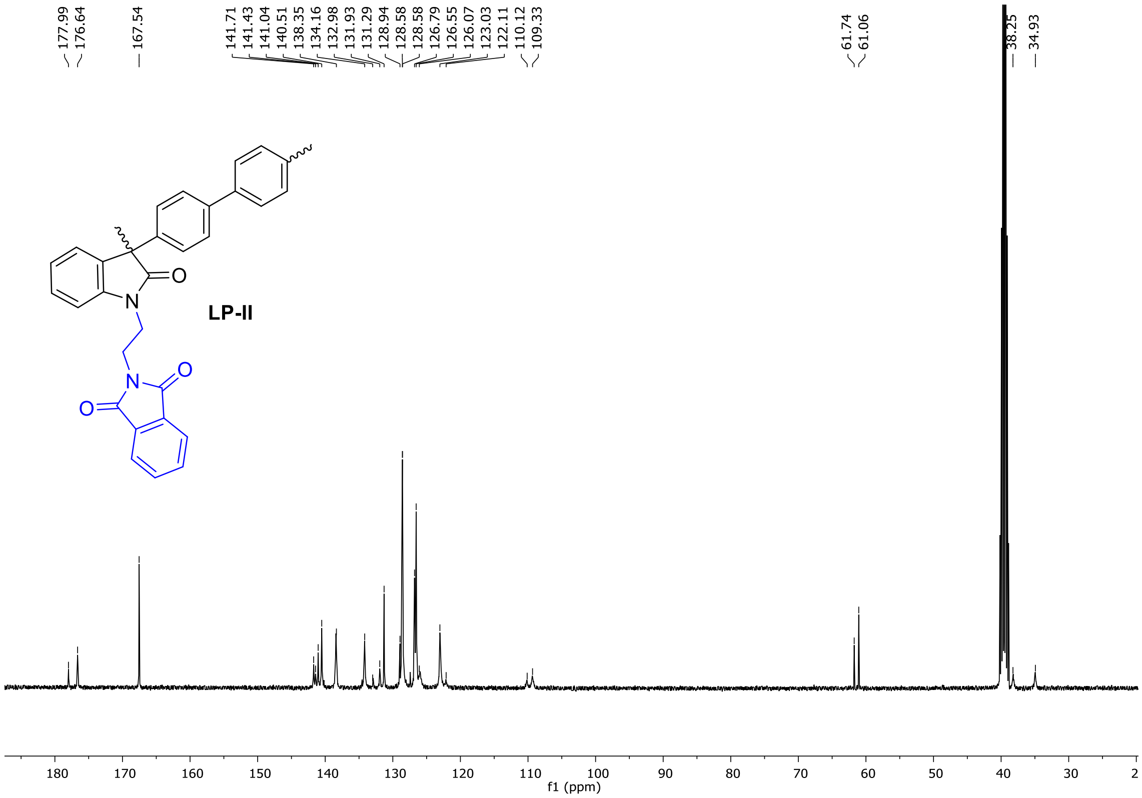

Comparison of ^{13}C {^{1} H} NMR (101 MHz, DMSO-d^{6}) of **LP-I** and **LP-II**

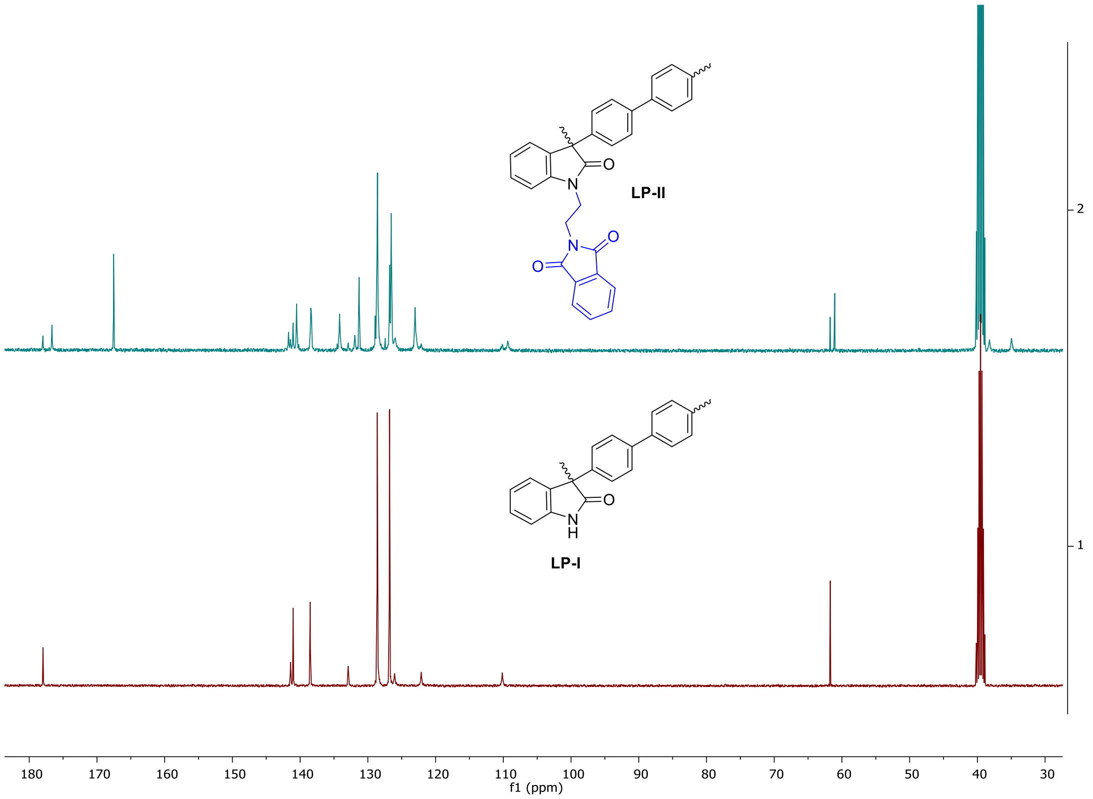

[1] H NMR (500 MHz, DMSO-d[6], 60 °C)/ ^{13}C {[1] H} NMR (126 MHz, DMSO-d[6], 60 °C) of **LP-III**

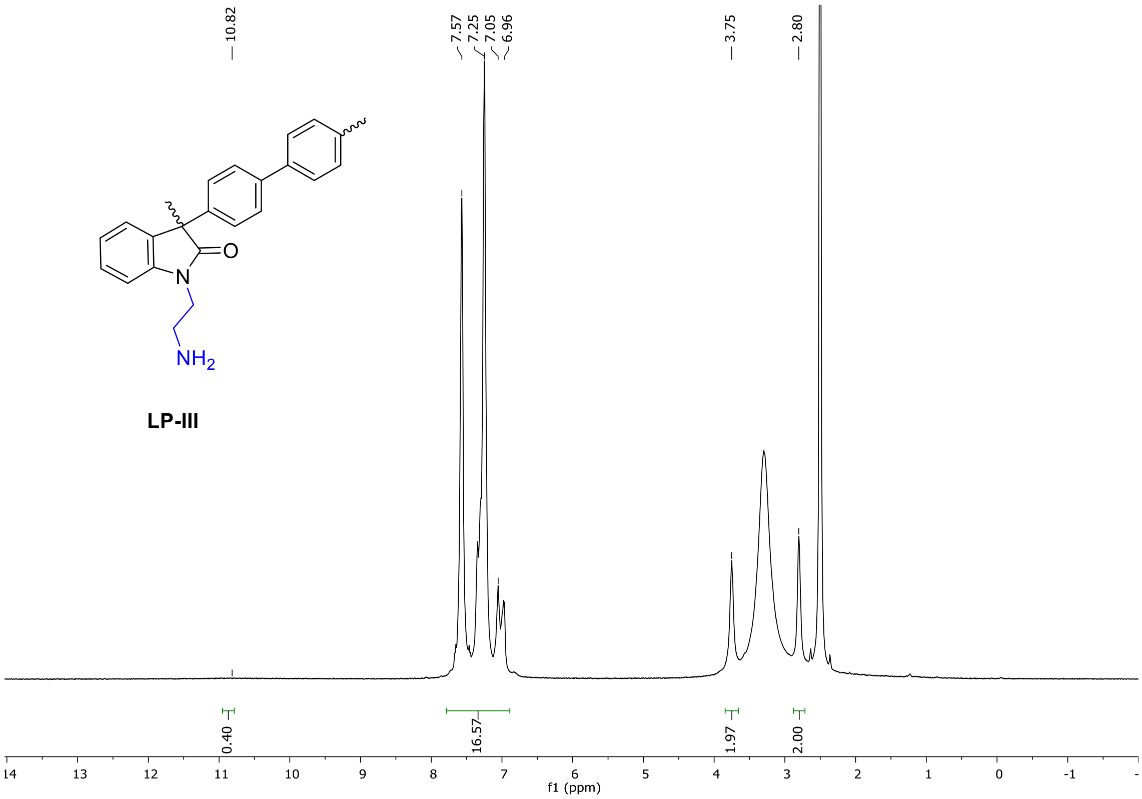

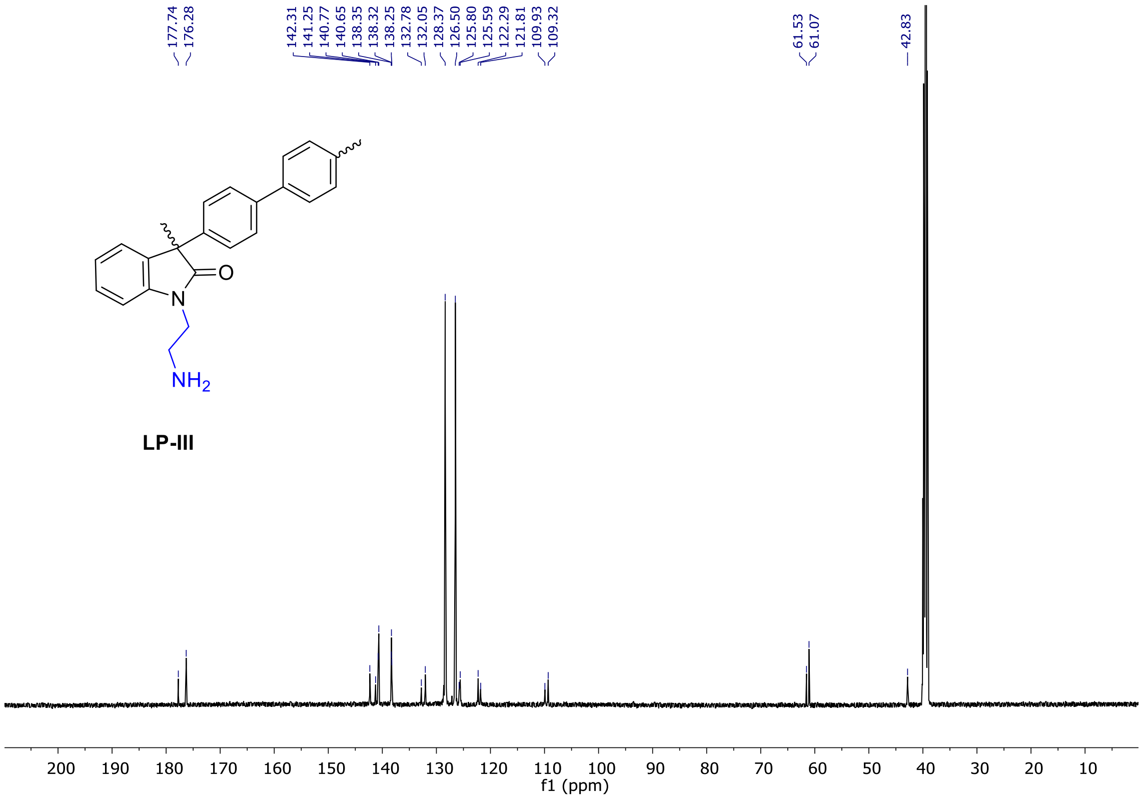

[1] H NMR (500 MHz, DMSO-d[6], 60 °C) / ^{13}C {[1] H} NMR (126 MHz, DMSO-d[6], 60 °C) of **LP-IV**

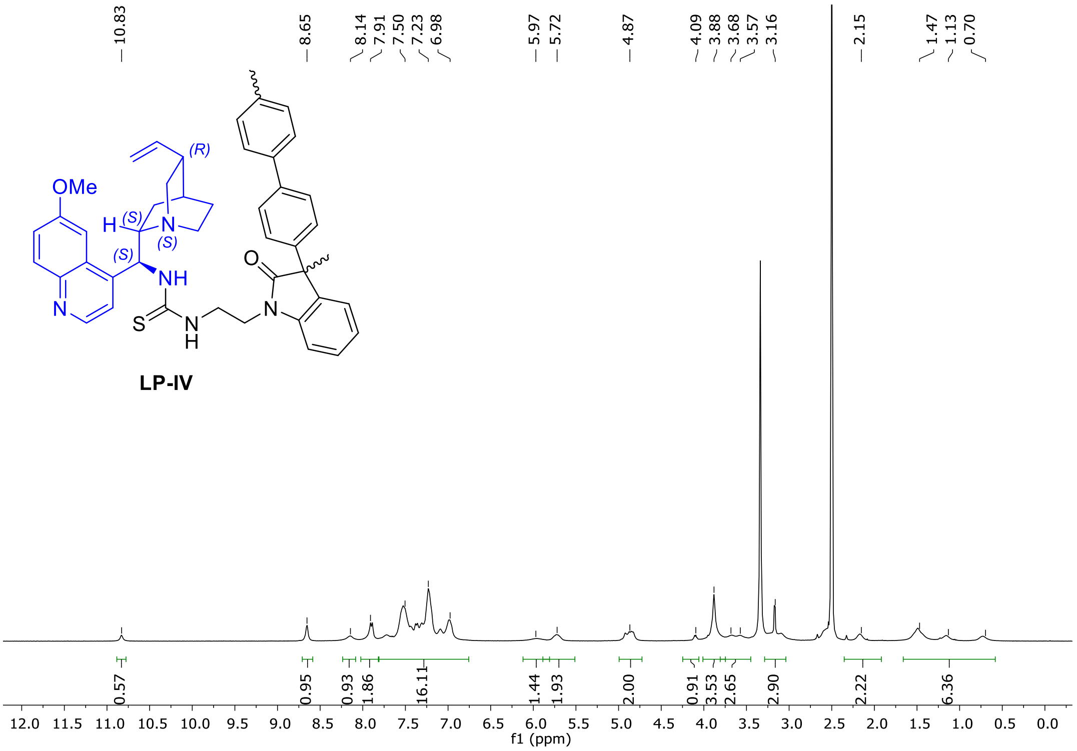

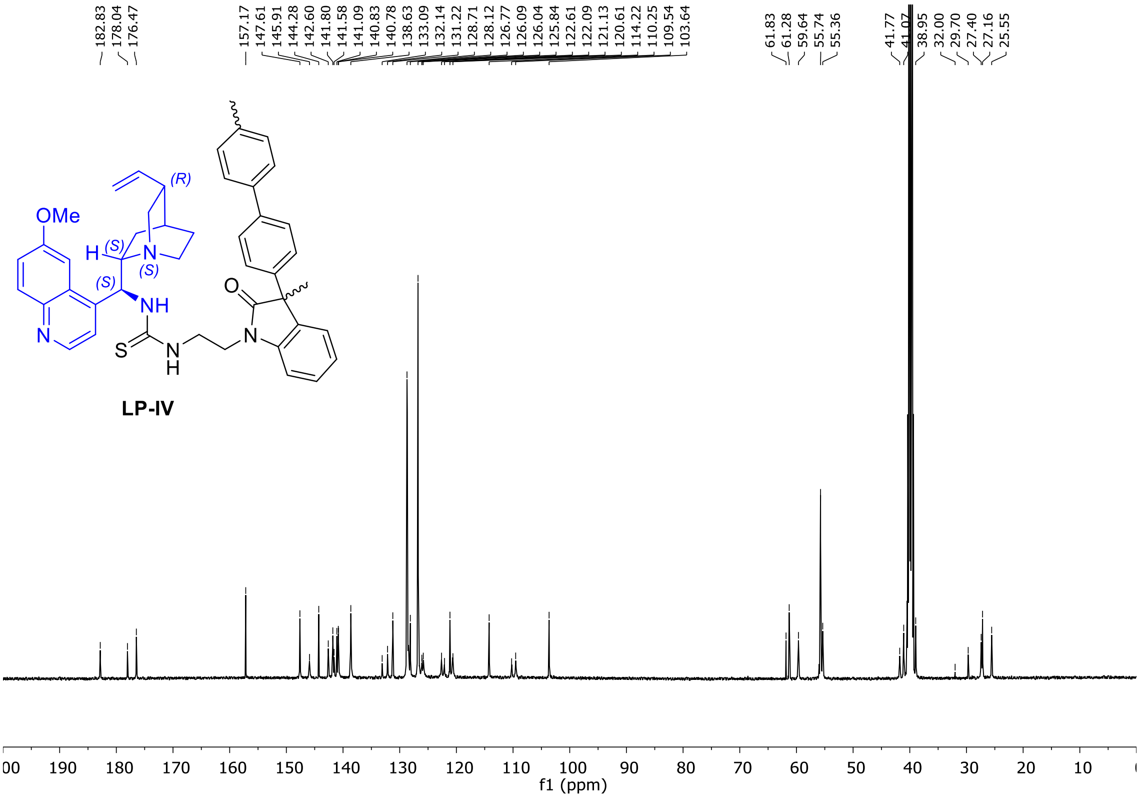

Comparison of ^{1} H NMR (500 MHz, DMSO-d^{6}, 60 °C) of **LP-IV** and **C1**

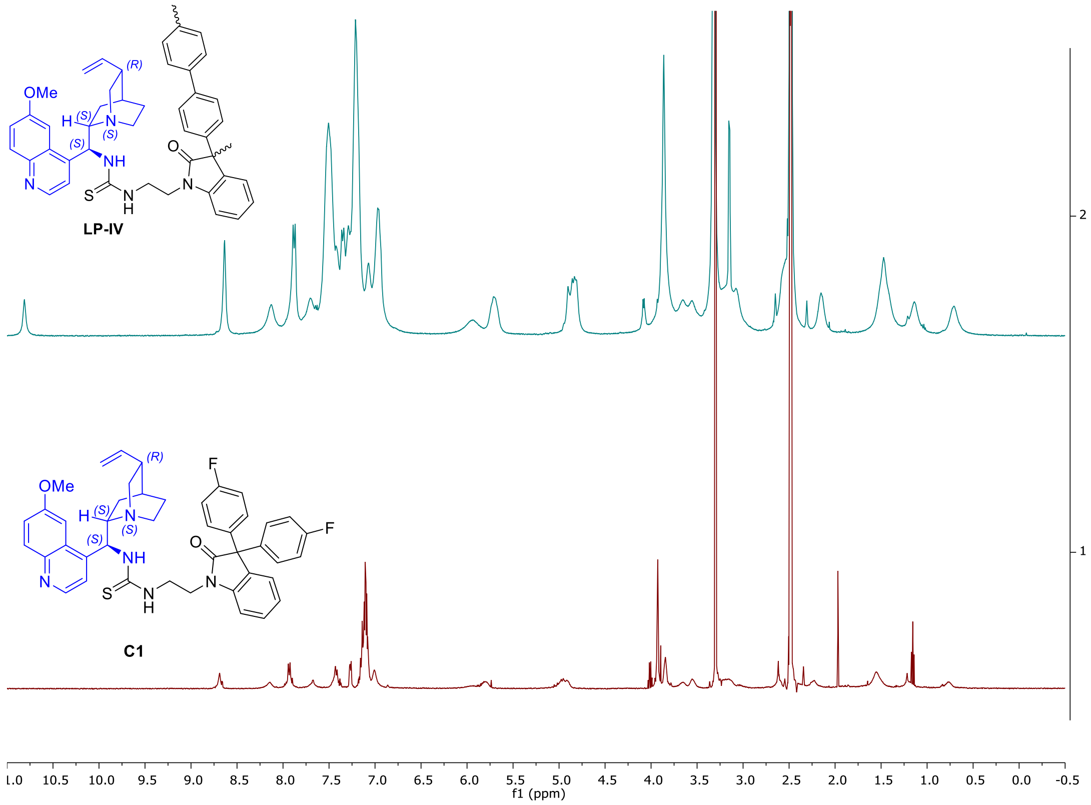

2. IR Spectra of polymers LP I-IV.

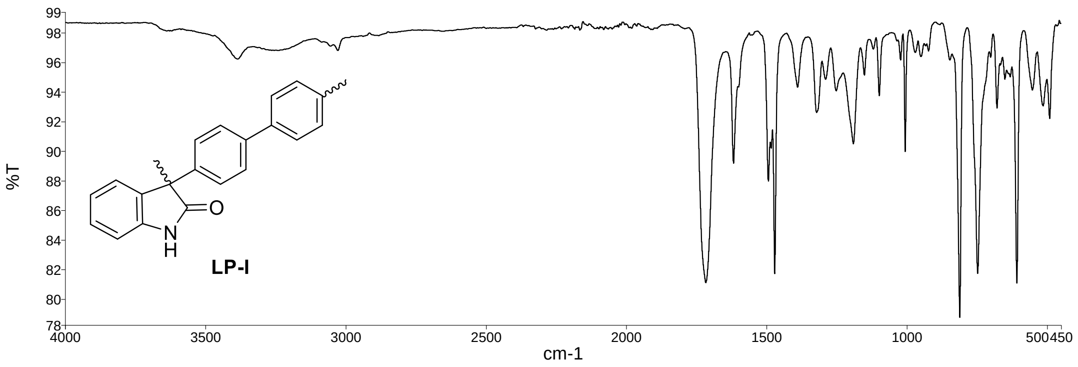

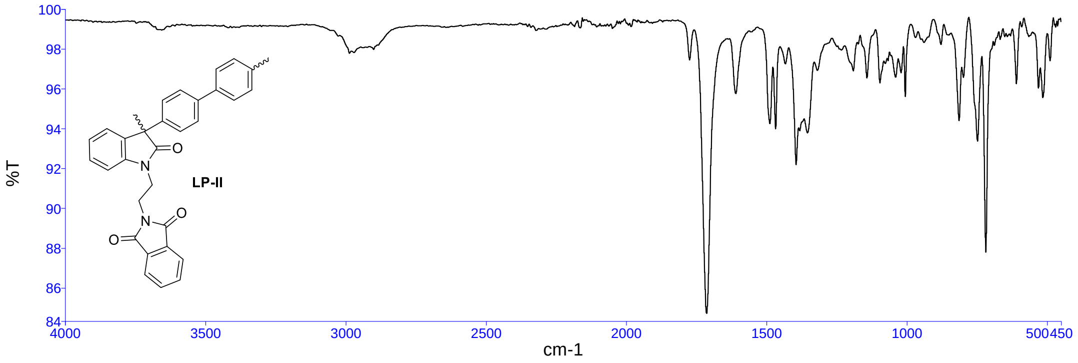

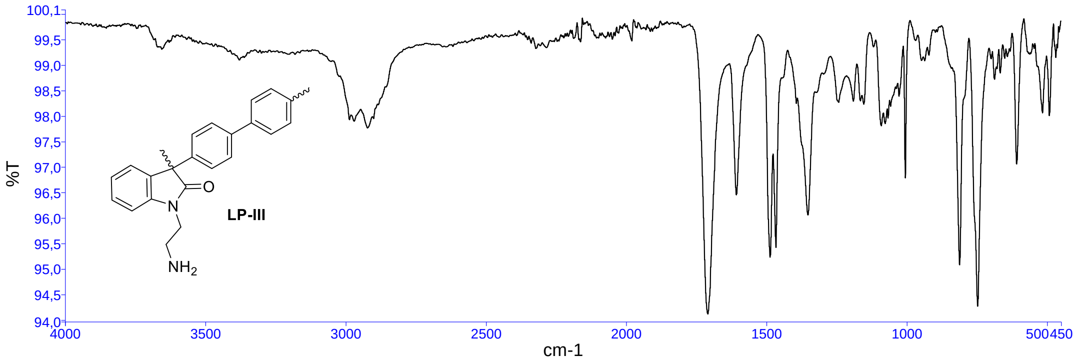

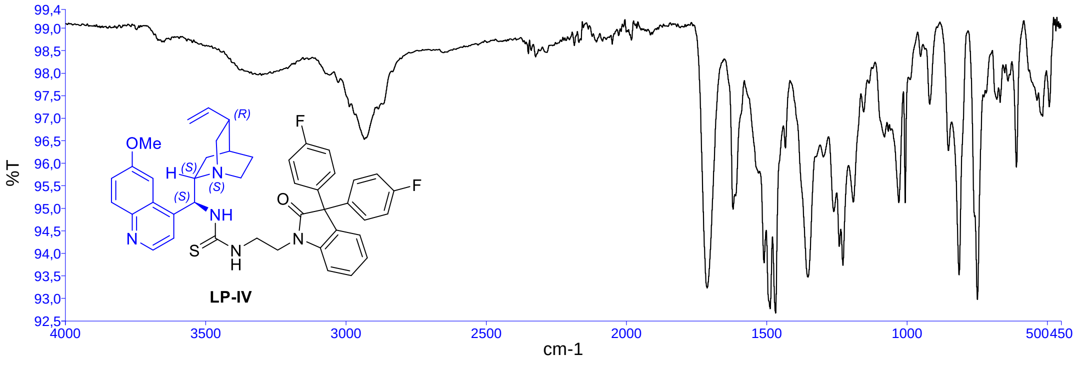

3. TGA thermograms.

Thermogravimetric analysis (TGA) thermograms comparison of polymers **I**–**IV** under nitrogen atmosphere

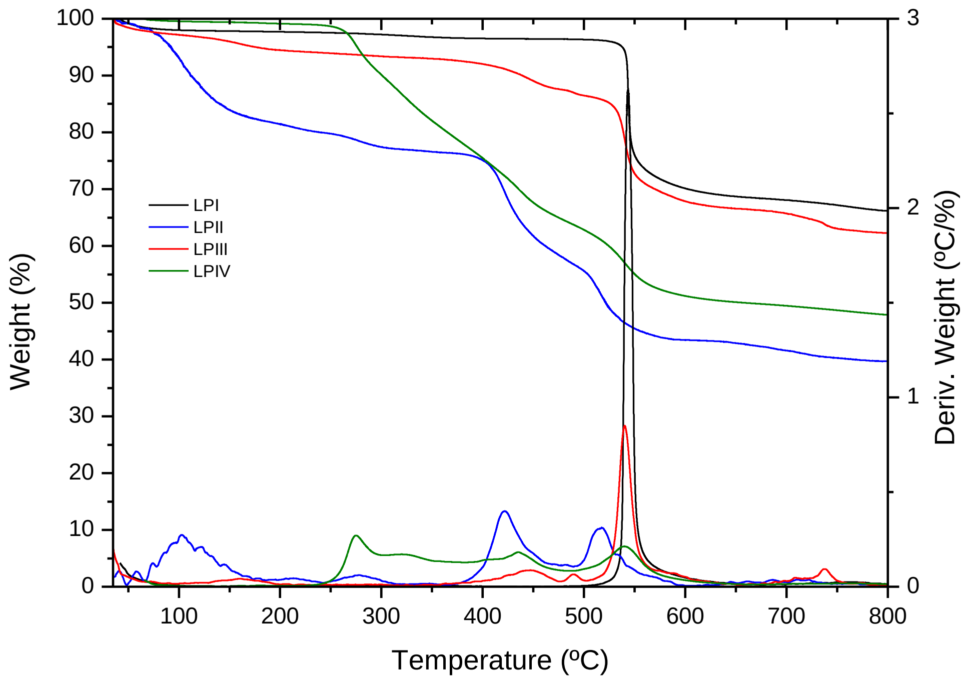

4.- Swelling Ratio of Polymer LP-IV in Several Solvents

Data for each solvent was determined gravimetrically and measured as grams of swollen polymer (W_s) per grams of dry polymer (W_d). Polymer LP-**IV** (60 mg) was weighted into a tared sealed tube and different solvents (5 mL) were added into the tube. The tubes were then closed and shaken for 3 h at room temperature. Afterward, centrifugation (30 min at 4500 rpm) was carried out, and a syringe was used to remove the excess of solvent. Then, the tubes were immediately weighted to obtain the weight of the absorbed solvent. Swelling ratios (SR, %) were also corrected in volume considering the corresponding density of the solvents ($d_{solvent}$).

**LP-IV**	W_d (mg)	W_S-W_d (mg)	Swelling ratio (SR, %)	$d_{solvent}$ (g/mL)	SR* (%)
Toluene	60.0	21.9	36.5	0.867	42.1
DCM	60.9	35.0	57.5	1.327	43.3
DCM/Toluene 1:1	60.2	28.9	48.0	0.993	48.3

SR=100(W_S-W_D)/Wd. SR*=SR/$d_{solvent}$

It was observed that SR values were higher when DCM was used. Therefore, due to the existing differences between the pure solvents (and solvent mixture) densities, it was considered appropriate to correct the SR value to SR* which indicates the amount of absorbed solvent volume per gram of dry polymer. It was observed that the swelling values were very similar in this case for SR*.

4. HPLC Profiles of the isolated compounds.

Dibenzyl (*R*)-1-(4-benzyl-3-methyl-5-oxo-1-phenyl-4,5-dihydro-1*H*-pyrazol-4-yl)hydrazine-1,2-dicarboxylate (13a).

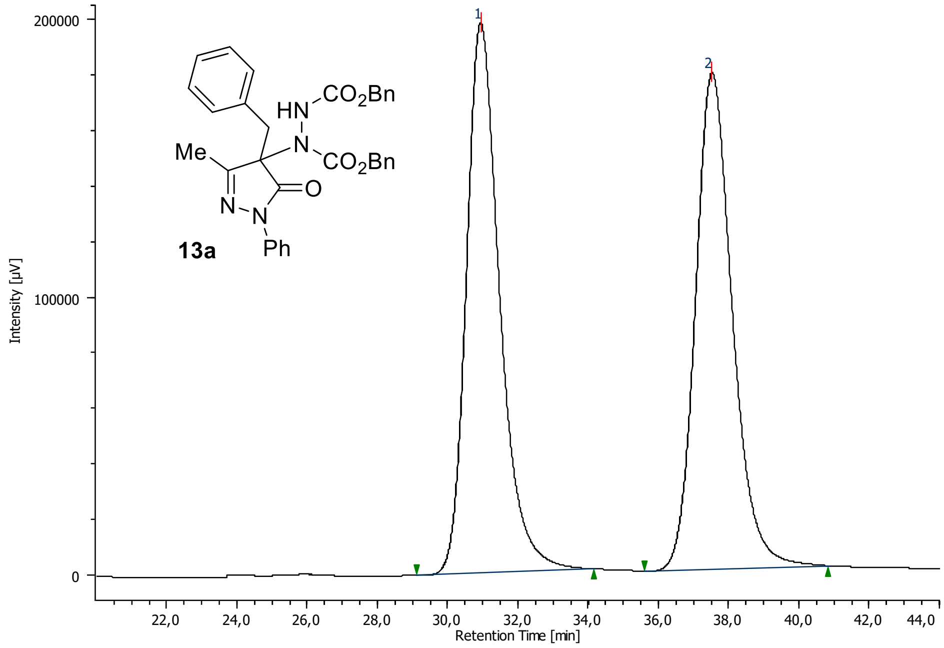

Peak Number	t_R	Area	Height	Area%	Height%	Symmetry Factor
1	**30,925**	13008997	197520	**50,035**	52,578	1,322
2	**37,500**	12990869	178150	**49,965**	47,422	1,212

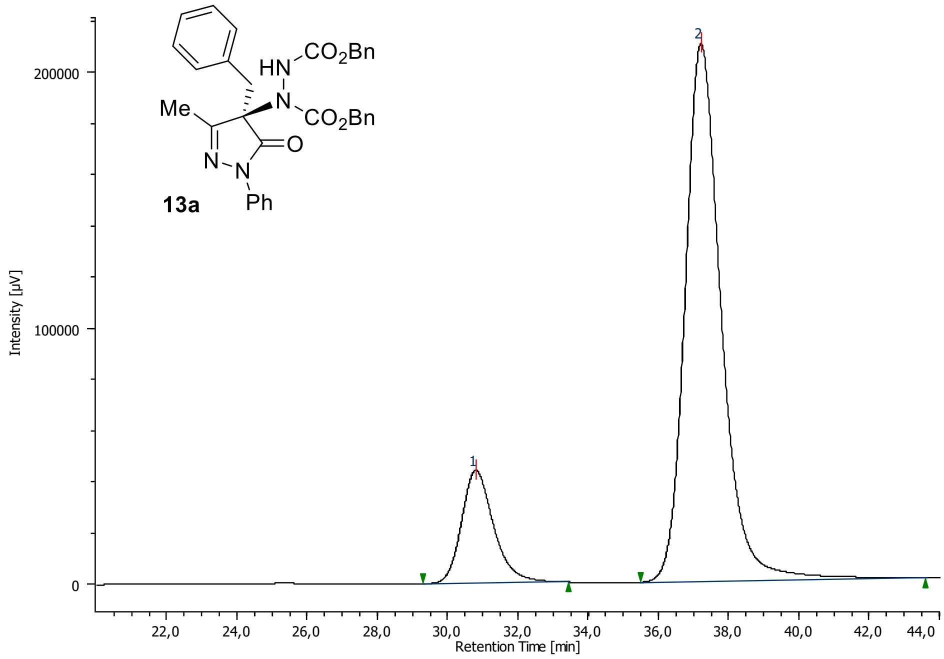

Peak Number	t_R	Area	Height	Area%	Height%	Symmetry Factor
1	**30,783**	2809905	44107	**15,820**	17,375	1,276
2	**37,183**	14951586	209752	**84,180**	82,625	1,246

Diisopropyl (*R*)-1-(4-benzyl-3-methyl-5-oxo-1-phenyl-4,5-dihydro-1*H*-pyrazol-4-yl)hydrazine-1,2-dicarboxylate (14a).

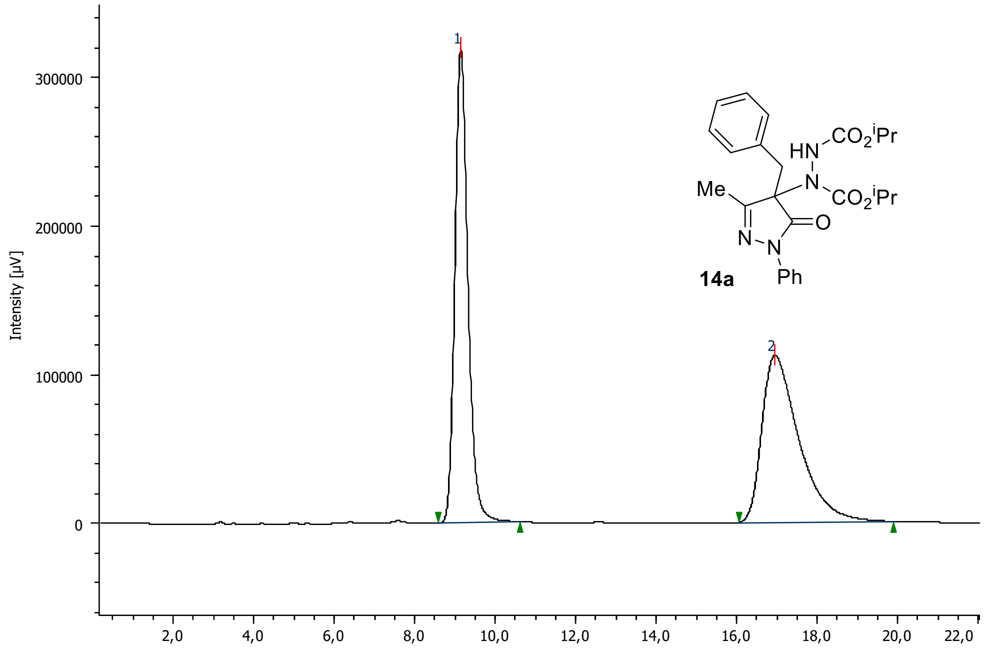

Peak Number	t_R	Area	Height	Area%	Height%	Symmetry Factor
1	**9,133**	7229334	318688	**50,160**	73,956	1,217
2	**16,925**	7183171	112230	**49,840**	26,044	1,733

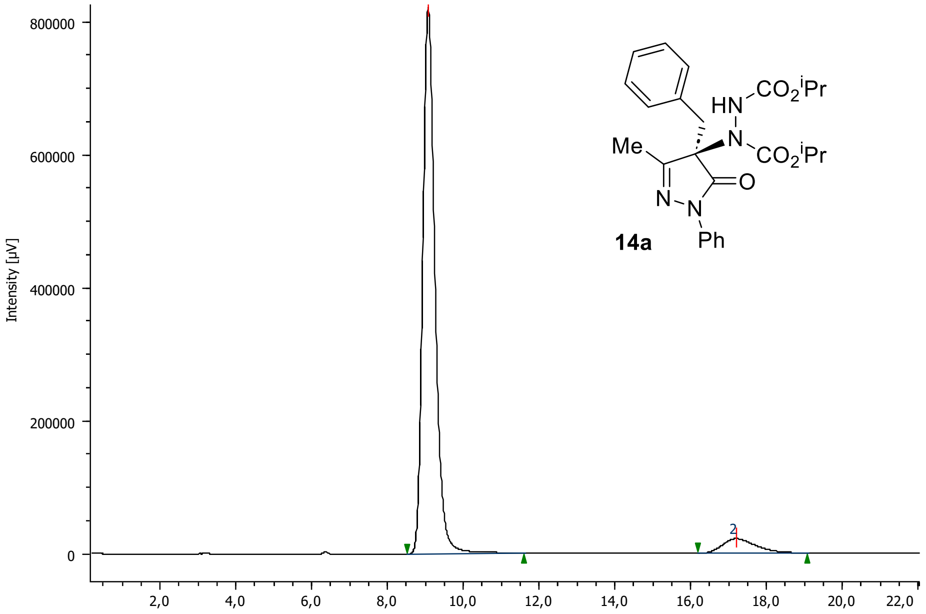

Peak Name	t_R	Area	Height	Area%	Height%	Symmetry Factor
1	**9,058**	18350031	820233	**93,149**	97,413	1,276
2	**17,175**	1349710	21782	**6,851**	2,587	1,388

Di-*tert*-Butyl (*R*)-1-(4-benzyl-3-methyl-5-oxo-1-phenyl-4,5-dihydro-1*H*-pyrazol-4-yl)hydrazine-1,2-dicarboxylate (15a).

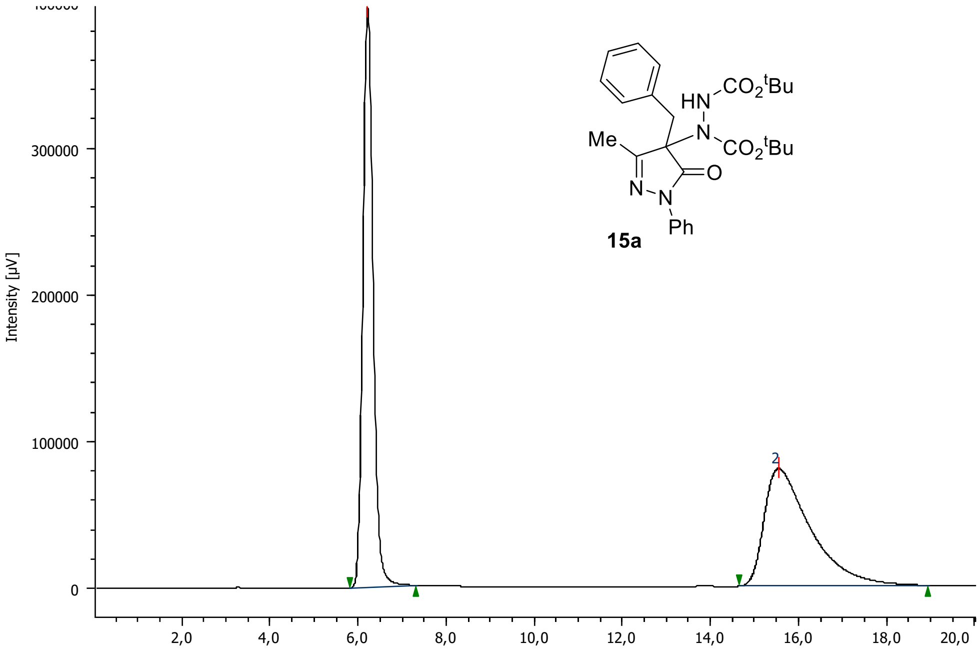

Peak Number	t_R	*Area*	*Height*	*Area%*	*Height%*	*Symmetry Factor*
1	**6,208**	6364996	394589	**52,153**	83,170	1,167
2	**15,533**	5839433	79845	**47,847**	16,830	2,050

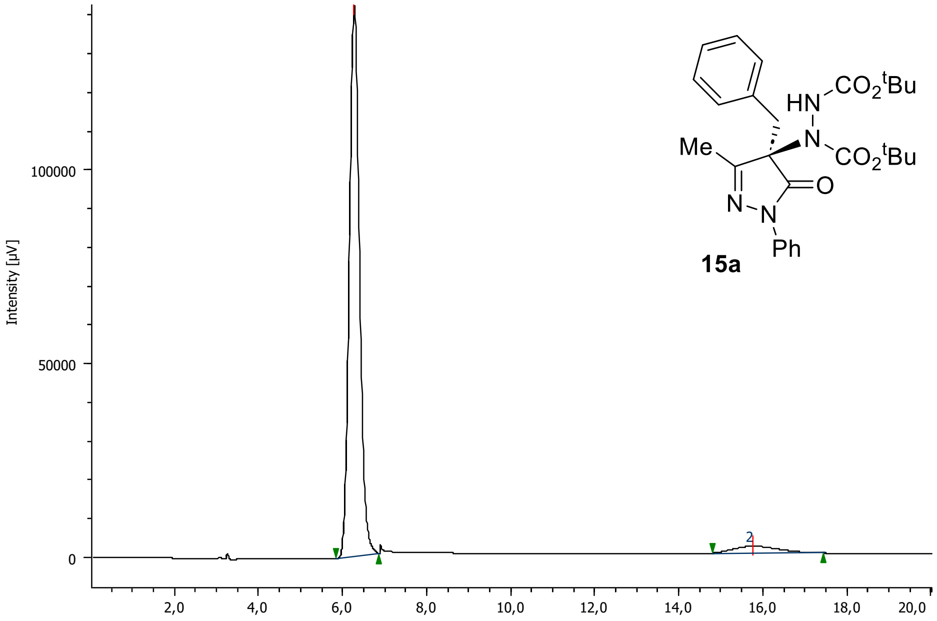

Peak Number	t_R	*Area*	*Height*	*Area%*	*Height%*	*Symmetry Factor*
1	**6,267**	2394692	141884	**94,836**	98,780	1,127
2	**15,742**	130404	1753	**5,164**	1,220	1,312

Di-*tert*-butyl (*R*)-1-(4-benzyl-3-ethyl-5-oxo-1-phenyl-4,5-dihydro-1*H*-pyrazol-4-yl)hydrazine-1,2-dicarboxylate (16b).

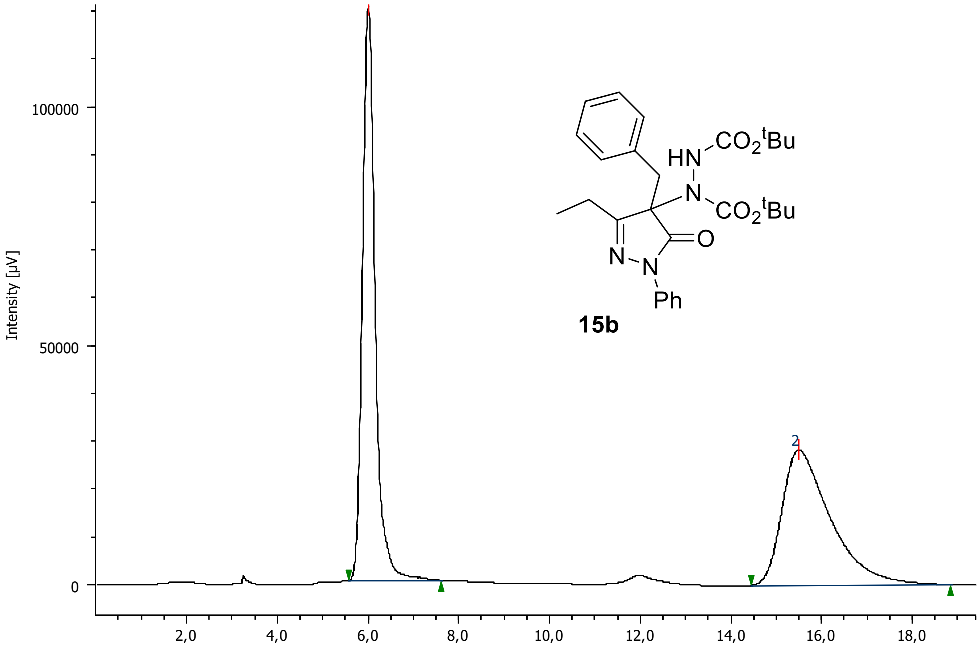

Peak Number	t_R	*Area*	*Height*	*Area%*	*Height%*	*Symmetry Factor*
1	**6,000**	2355954	120104	**52,861**	80,934	1,289
2	**15,475**	2100931	28294	**47,139**	19,066	1,759

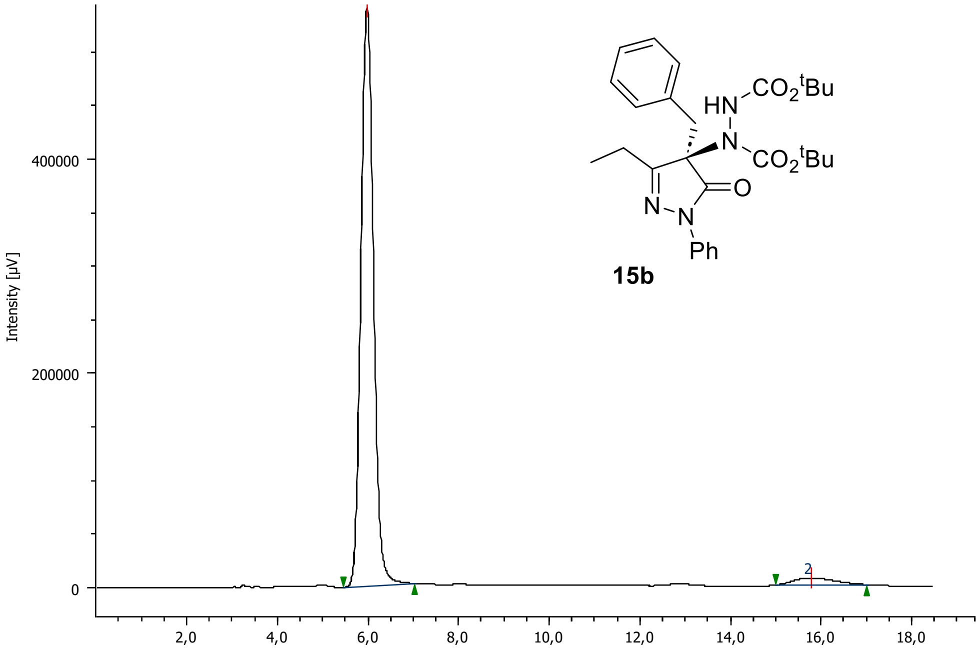

Peak Number	t_R	*Area*	*Height*	*Area%*	*Height%*	*Symmetry Factor*
1	**5,975**	10456722	539099	**96,197**	98,812	1,096
2	**15,767**	413363	6480	**3,803**	1,188	1,256

Di-*tert*-butyl (*R*)-1-(4-benzyl-3-isopropyl-5-oxo-1-phenyl-4,5-dihydro-1*H*-pyrazol-4-yl)hydrazine-1,2-dicarboxylate (15c).

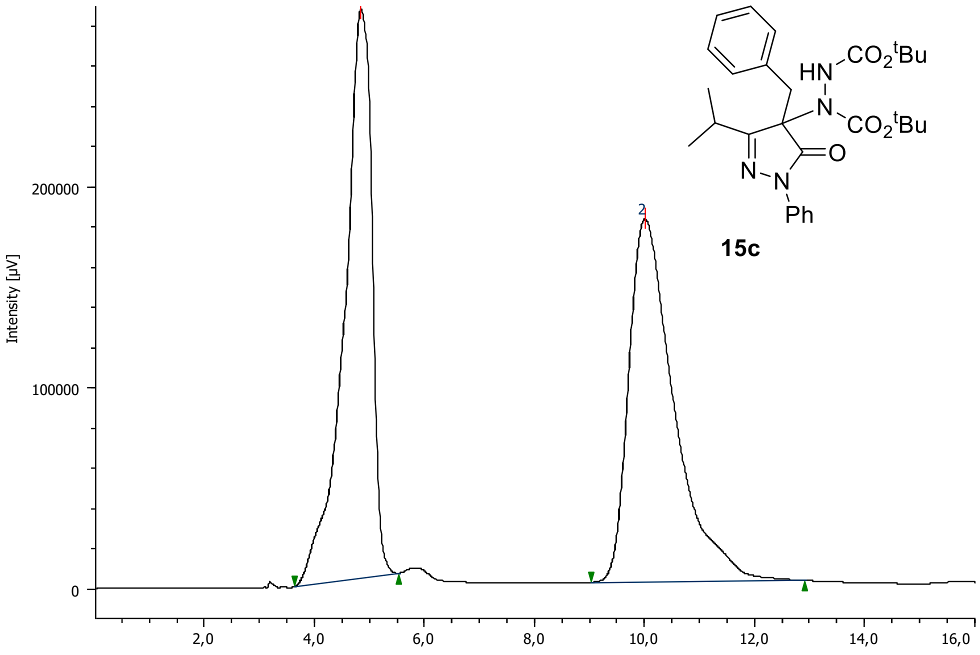

Peak Number	*t_R*	*Area*	*Height*	*Area%*	*Height%*	*Symmetry Factor*
1	**4,842**	9910141	282380	**48,887**	61,065	0,709
2	**10,000**	10361569	180049	**51,113**	38,935	1,802

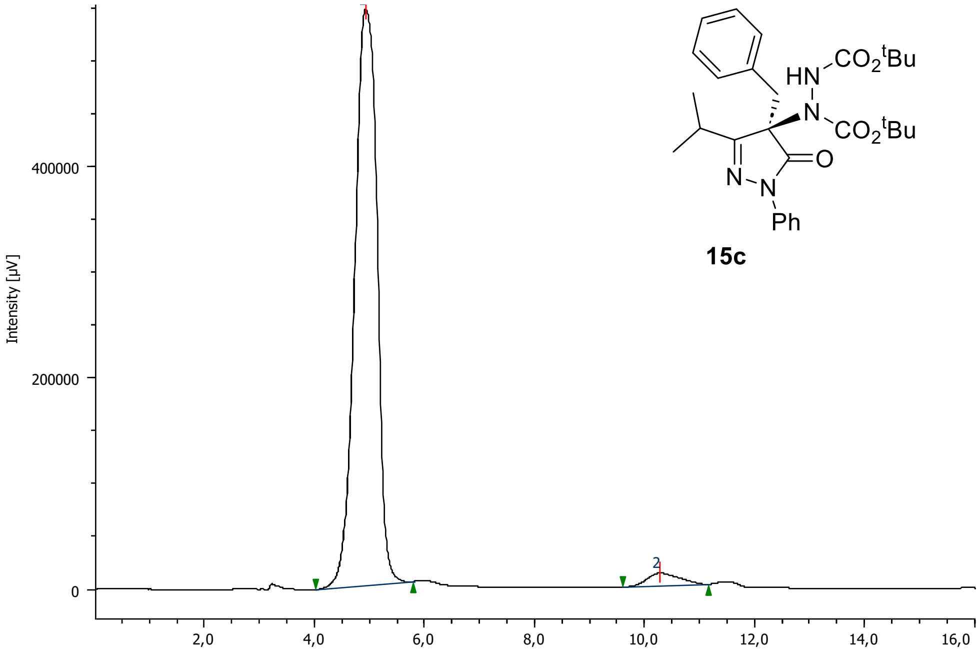

Peak Name	*t_R*	*Area*	*Height*	*Area%*	*Height%*	*Symmetry Factor*
1	**4,925**	15335068	543688	**96,870**	97,813	0,944
2	**10,275**	495475	12155	**3,130**	2,187	1,211

Di-*tert*-butyl (*R*)-1-(4-benzyl-5-oxo-1,3-diphenyl-4,5-dihydro-1*H*-pyrazol-4-yl)hydrazine-1,2-dicarboxylate (15d).

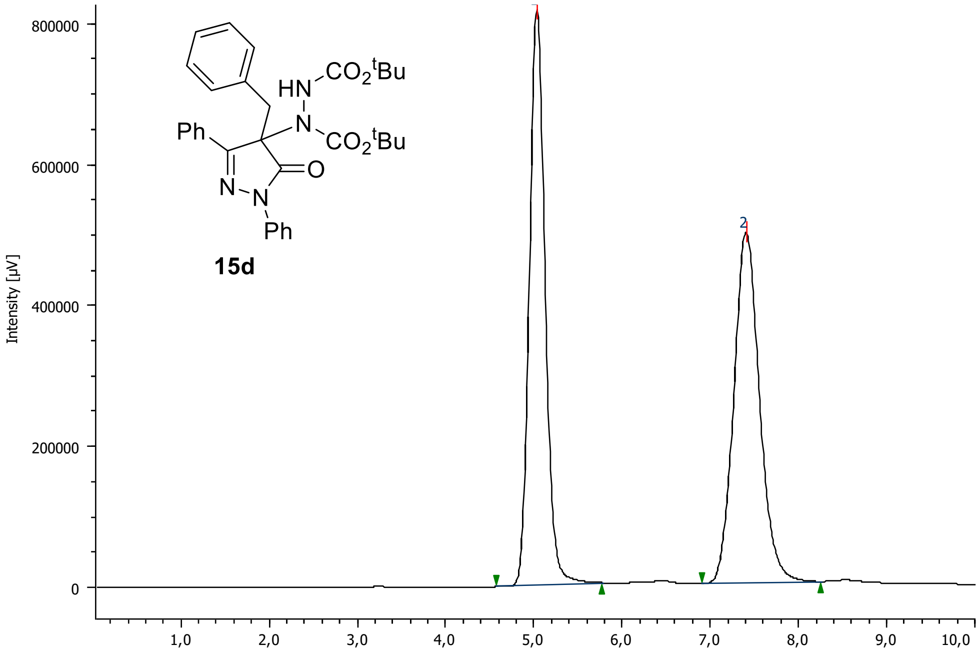

Peak Number	*t_R*	*Area*	*Height*	*Area%*	*Height%*	*Symmetry Factor*
1	**5,025**	10093563	815490	**50,135**	62,132	1,142
2	**7,400**	10039317	497019	**49,865**	37,868	1,113

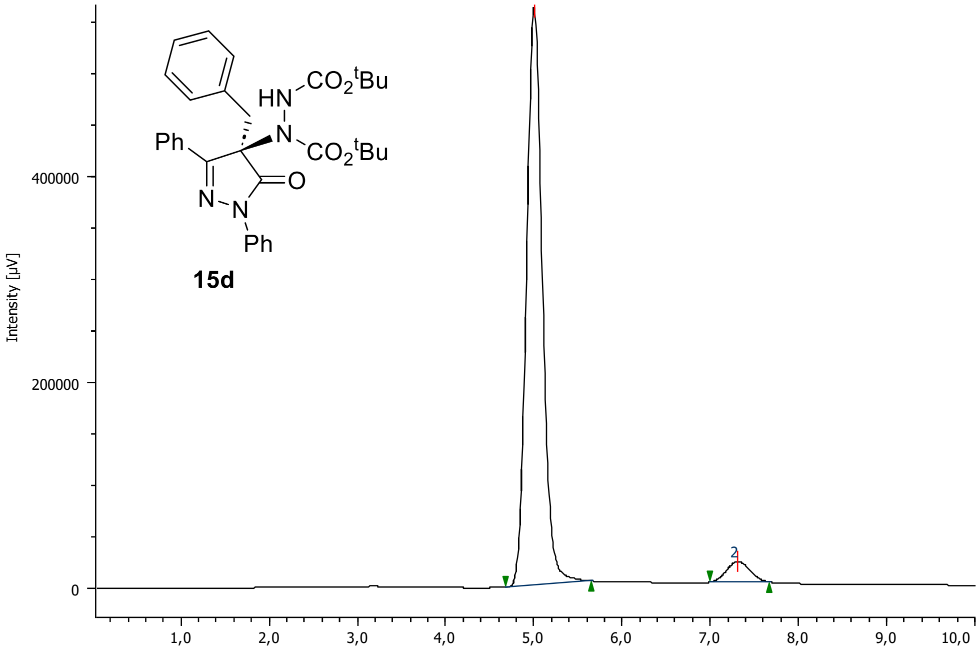

Peak Number	*t_R*	*Area*	*Height*	*Area%*	*Height%*	*Symmetry Factor*
1	**5,000**	7016855	560196	**95,022**	96,558	1,103
2	**7,300**	367590	19967	**4,978**	3,442	1,080

Di-*tert*-butyl (*R*)-1-(3-methyl-4-(4-methylbenzyl)-5-oxo-1-phenyl-4,5-dihydro-1*H*-pyrazol-4-yl)hydrazine-1,2-dicarboxylate (15e).

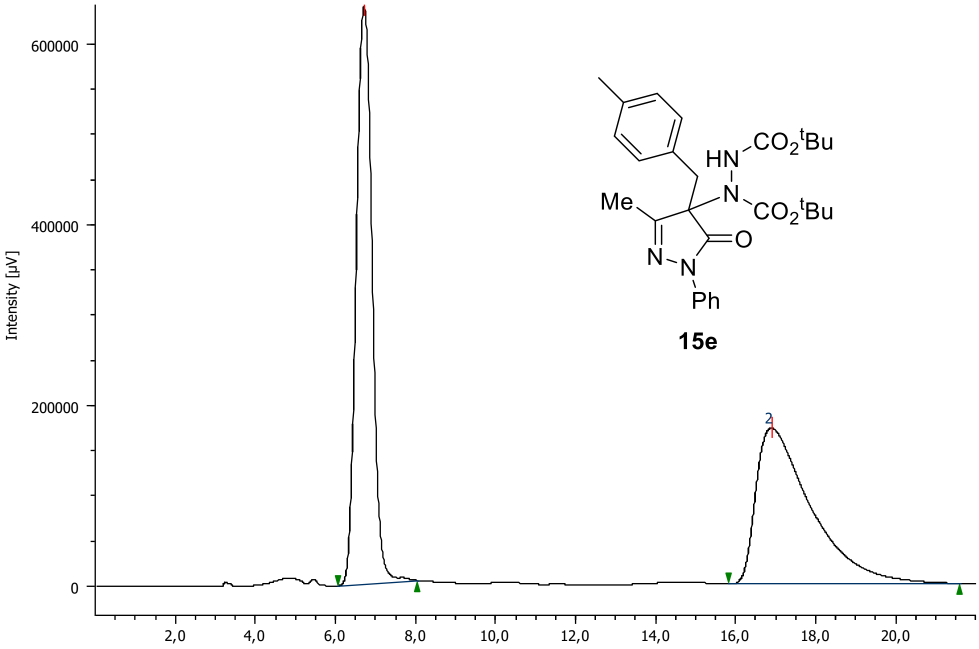

Peak Number	t_R	*Area*	*Height*	*Area%*	*Height%*	*Symmetry Factor*
1	**6,708**	17737273	638251	**51,743**	78,731	1,067
2	**16,875**	16542505	172426	**48,257**	21,269	2,399

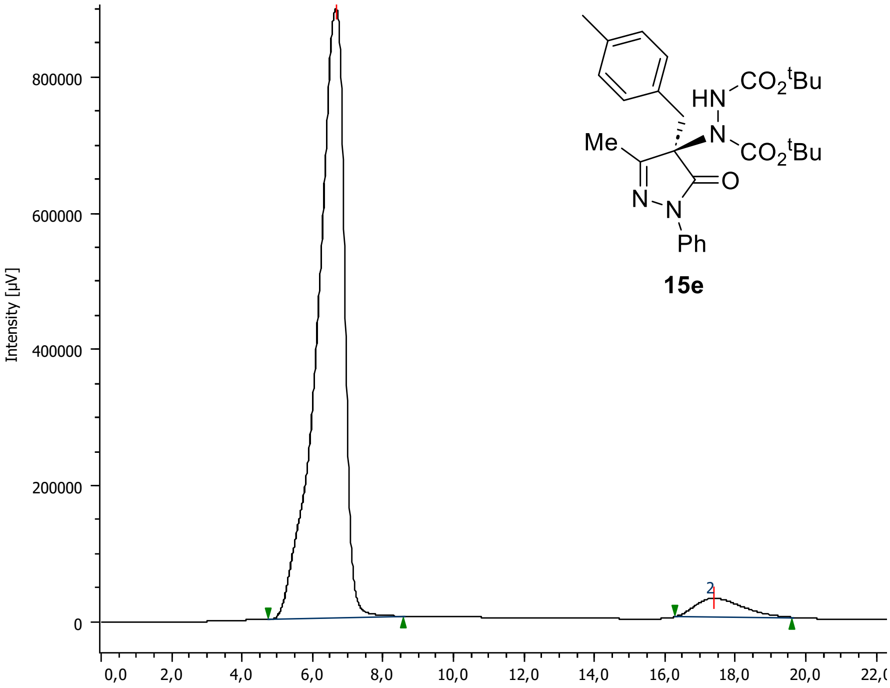

Peak Number	t_R	*Area*	*Height*	*Area%*	*Height%*	*Symmetry Factor*
1	**6,658**	47735784	892528	**94,856**	97,008	0,688
2	**17,367**	2588618	27533	**5,144**	2,992	1,426

Di-*tert*-butyl (*R*)-1-(4-(4-methoxybenzyl)-3-methyl-5-oxo-1-phenyl-4,5-dihydro-1*H*-pyrazol-4-yl)hydrazine-1,2-dicarboxylate (15f).

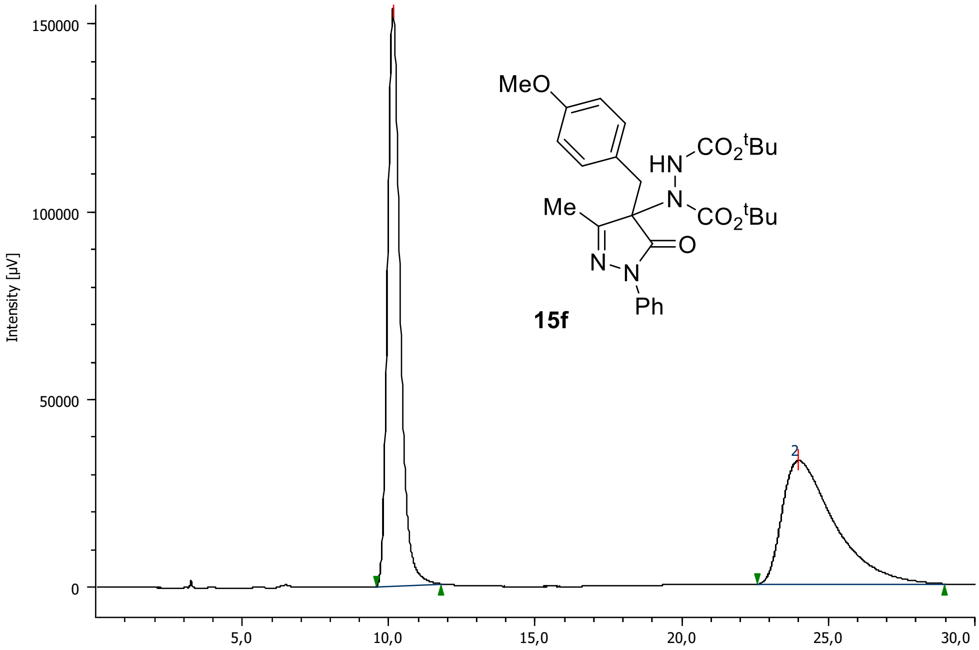

Peak Number	*tR*	*Area*	*Height*	*Area%*	*Height%*	*Symmetry Factor*
1	**10,133**	4578719	153524	**52,875**	82,365	1,306
2	**23,967**	4080815	32870	**47,125**	17,635	2,040

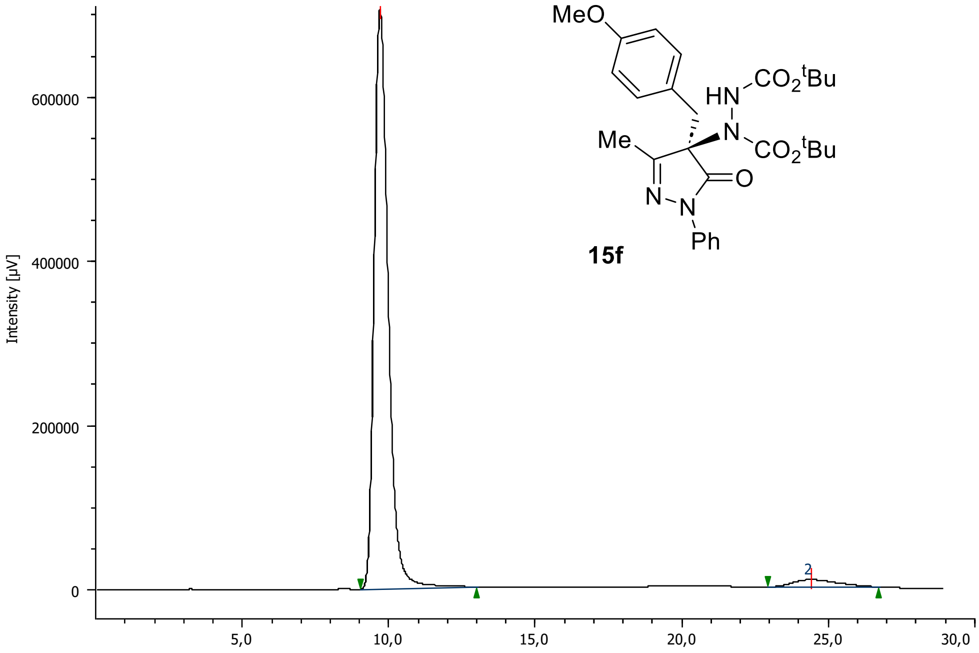

Peak Number	t_R	*Area*	*Height*	*Area%*	*Height%*	*Symmetry Factor*
1	**9,692**	23497125	704544	**96,058**	98,742	1,334
2	**24,392**	964293	8974	**3,942**	1,258	1,336

Di-*tert*-butyl (*R*)-1-(4-(4-bromobenzyl)-3-methyl-5-oxo-1-phenyl-4,5-dihydro-1*H*-pyrazol-4-yl)hydrazine-1,2-dicarboxylate (15g).

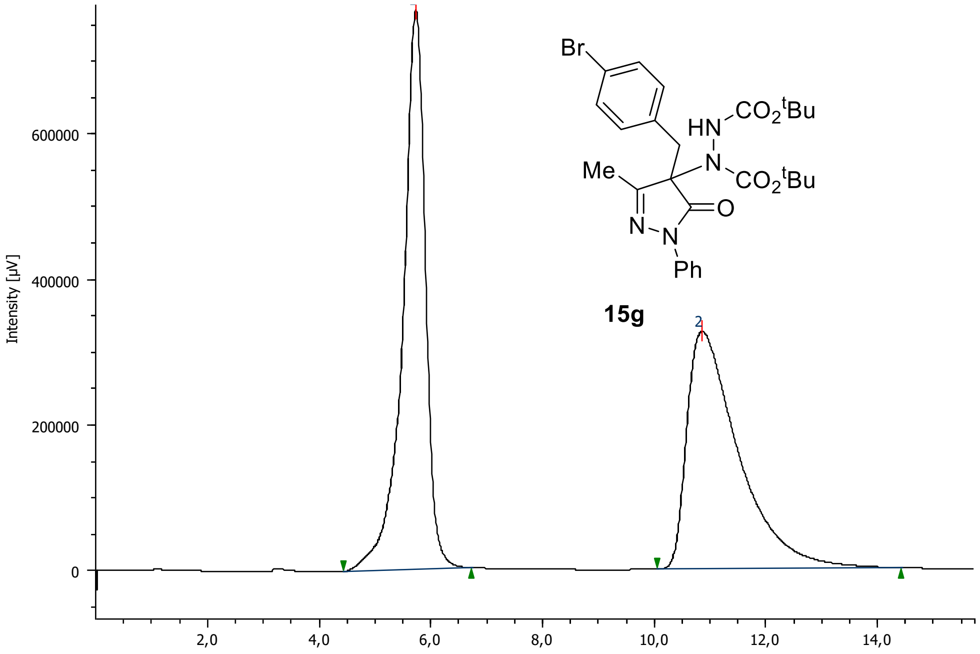

Peak Number	t_R	*Area*	*Height*	*Area%*	*Height%*	*Symmetry Factor*
1	**5,725**	21691120	767536	**50,783**	70,189	0,796
2	**10,842**	21022061	325996	**49,217**	29,811	2,282

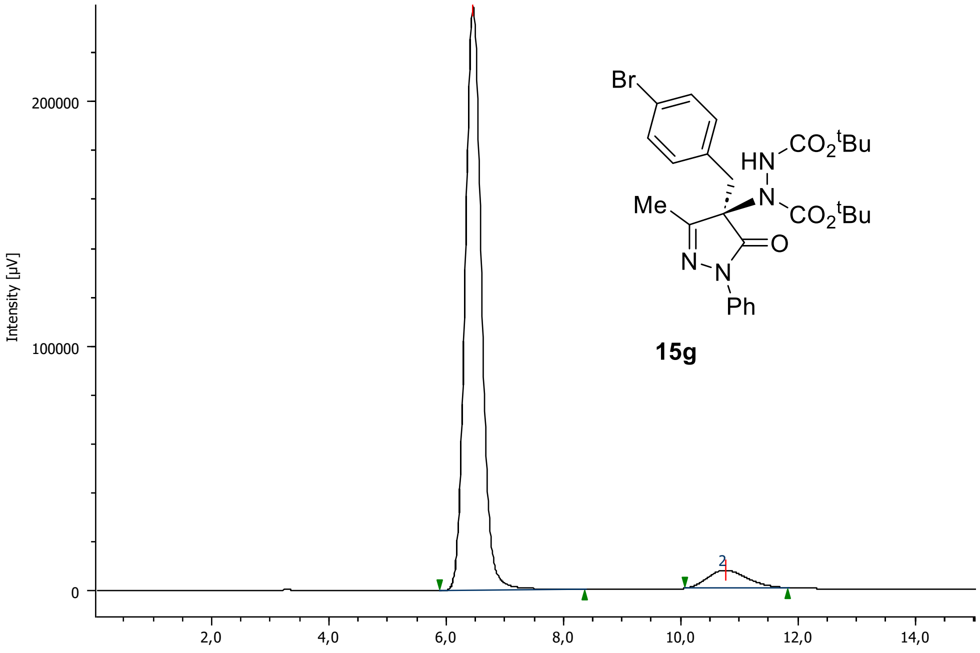

Peak Number	t_R	*Area*	*Height*	*Area%*	*Height%*	*Symmetry Factor*
1	**6,450**	4612247	238295	**93,025**	97,006	1,101
2	**10,750**	345819	7354	**6,975**	2,994	1,228

Di-*tert*-butyl (*R*)-1-(3-methyl-5-oxo-1-phenyl-4-(4-(trifluoromethyl)benzyl)-4,5-dihydro-1*H*-pyrazol-4-yl)hydrazine-1,2-dicarboxylate (15h).

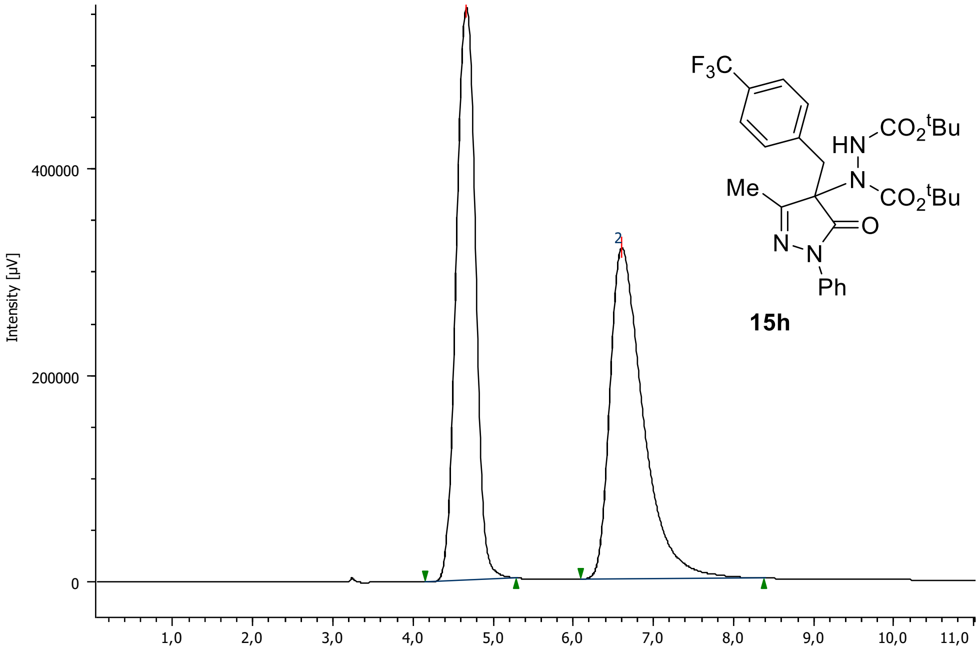

Peak Number	t_R	*Area*	*Height*	*Area%*	*Height%*	*Symmetry Factor*
1	**4,658**	9357183	552648	**50,378**	63,345	1,005
2	**6,600**	9216715	319790	**49,622**	36,655	1,700

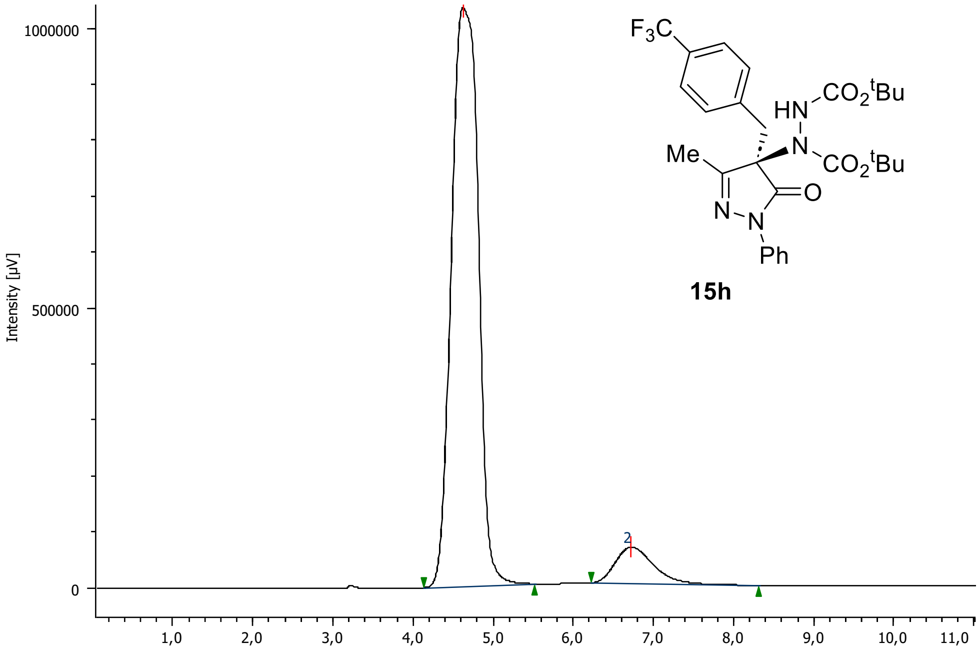

Peak Number	t_R	*Area*	*Height*	*Area%*	*Height%*	*Symmetry Factor*
1	**4,617**	24261314	1031652	**91,886**	94,092	1,101
2	**6,717**	2142481	64779	**8,114**	5,908	1,373

Di-*tert*-butyl (*R*)-1-(3-methyl-4-(4-nitrobenzyl)-5-oxo-1-phenyl-4,5-dihydro-1*H*-pyrazol-4-yl)hydrazine-1,2-dicarboxylate (15i).

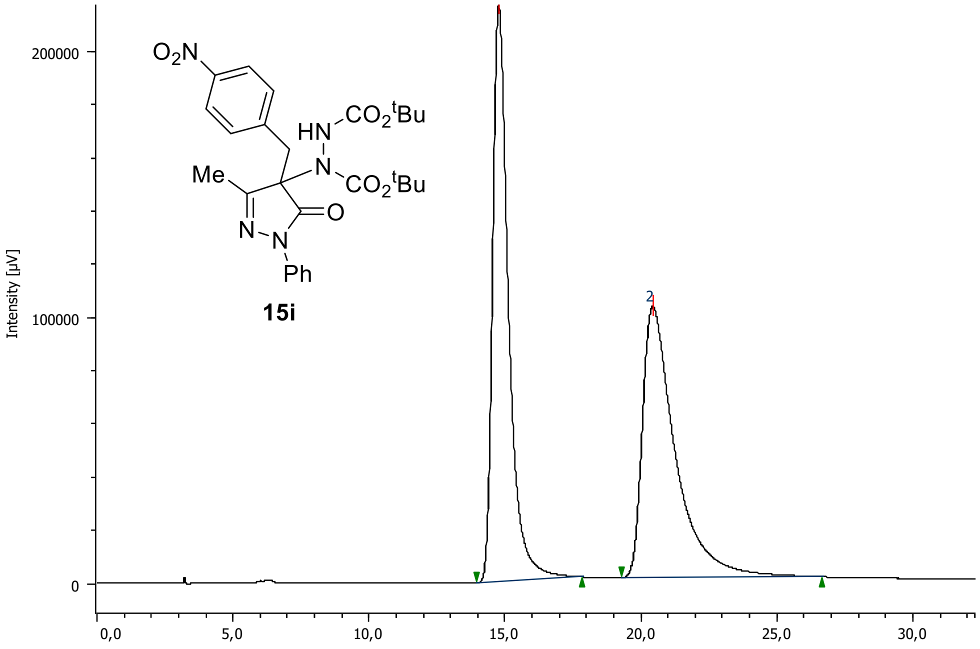

Peak Number	t_R	Area	Height	Area%	Height%	Symmetry Factor
1	**14,750**	8848162	216857	**50,719**	68,057	1,609
2	**20,408**	8597247	101782	**49,281**	31,943	2,168

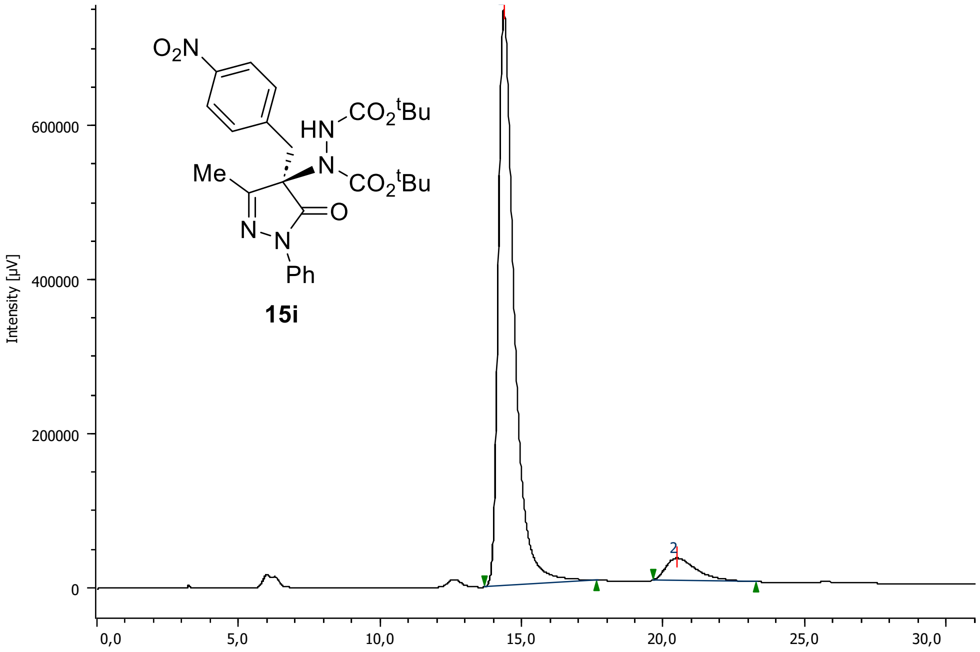

Peak Number	t_R	Area	Height	Area%	Height%	Symmetry Factor
1	**14,358**	29291275	747038	**92,972**	96,260	1,724
2	**20,467**	2214183	30208	**7,028**	3,740	1,783

Di-*tert*-butyl (*R*)-1-(3-methyl-4-(2-nitrobenzyl)-5-oxo-1-phenyl-4,5-dihydro-1*H*-pyrazol-4-yl)hydrazine-1,2-dicarboxylate (15j).

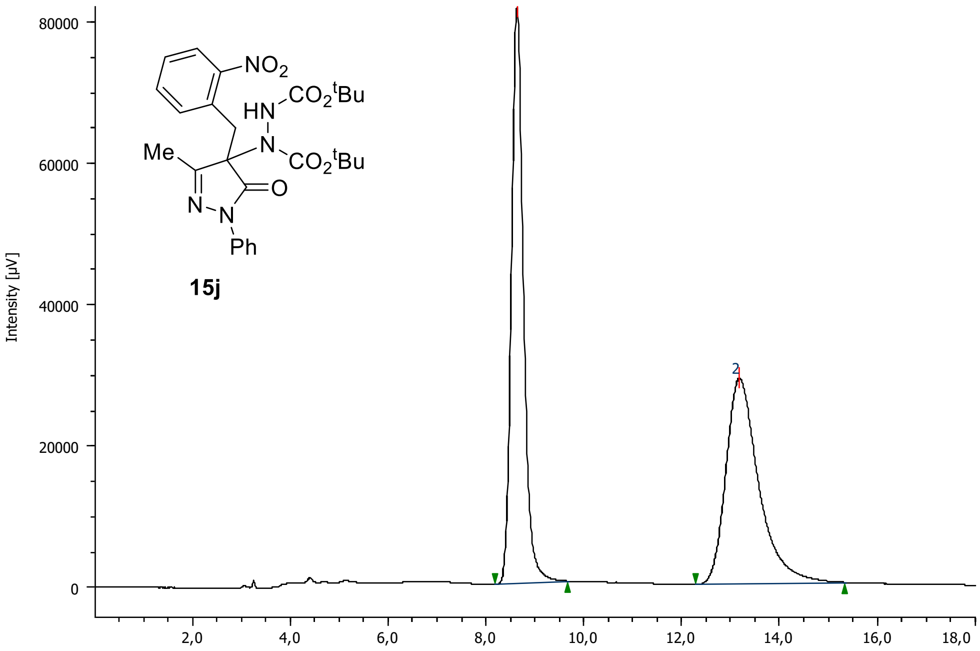

Peak Number	*t_R*	*Area*	*Height*	*Area%*	*Height%*	*Symmetry Factor*
1	**8,625**	1398039	81334	**50,729**	73,690	1,193
2	**13,167**	1357878	29040	**49,271**	26,310	1,461

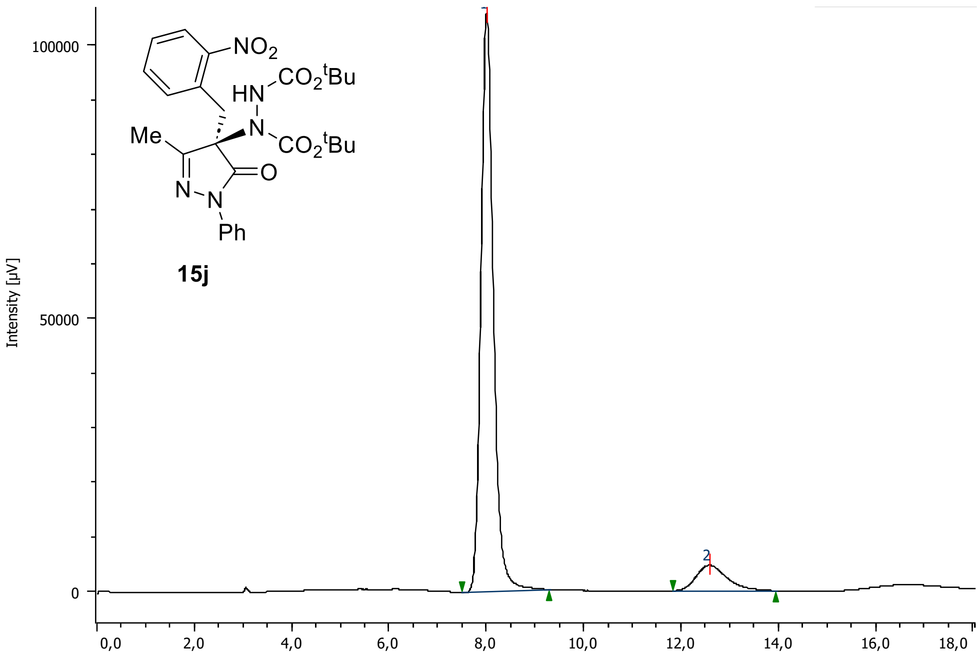

Peak Number	*t_R*	*Area*	*Height*	*Area%*	*Height%*	*Symmetry Factor*
1	**8,000**	1878710	105754	**90,056**	95,773	1,198
2	**12,575**	207459	4667	**9,944**	4,227	1,378

Di-*tert*-butyl (*R*)-1-(4-(2,6-dichlorobenzyl)-3-methyl-5-oxo-1-phenyl-4,5-dihydro-1*H*-pyrazol-4-yl)hydrazine-1,2-dicarboxylate (15k).

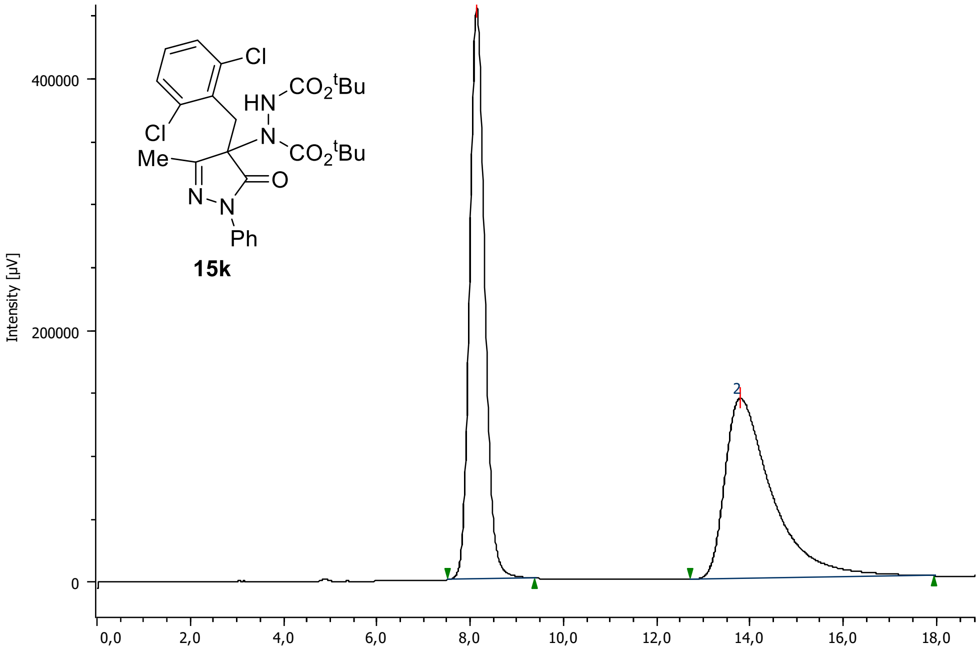

Peak Number	t_R	*Area*	*Height*	*Area%*	*Height%*	*Symmetry Factor*
1	**8,133**	10184042	453311	**50,080**	75,989	1,135
2	**13,775**	10151429	143238	**49,920**	24,011	2,055

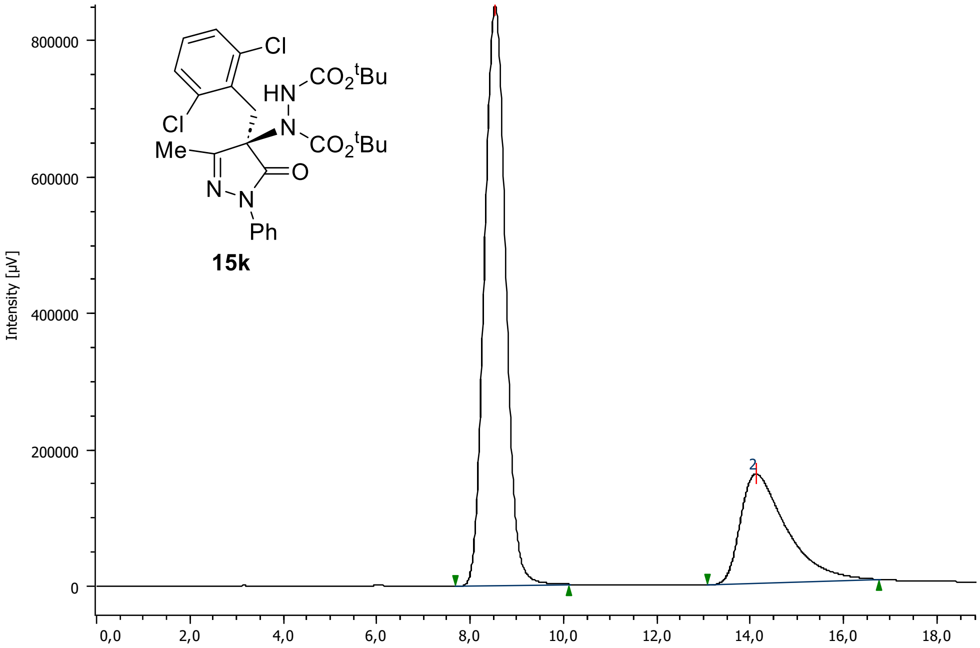

Peak Number	t_R	*Area*	*Height*	*Area%*	*Height%*	*Symmetry Factor*
1	**8,525**	26775395	848273	**70,859**	84,107	1,063
2	**14,108**	11011719	160290	**29,141**	15,893	1,879

Di-*tert*-butyl (*R*)-1-(4-benzyl-1-(4-chlorophenyl)-3-methyl-5-oxo-4,5-dihydro-1*H*-pyrazol-4-yl)hydrazine-1,2-dicarboxylate (15l).

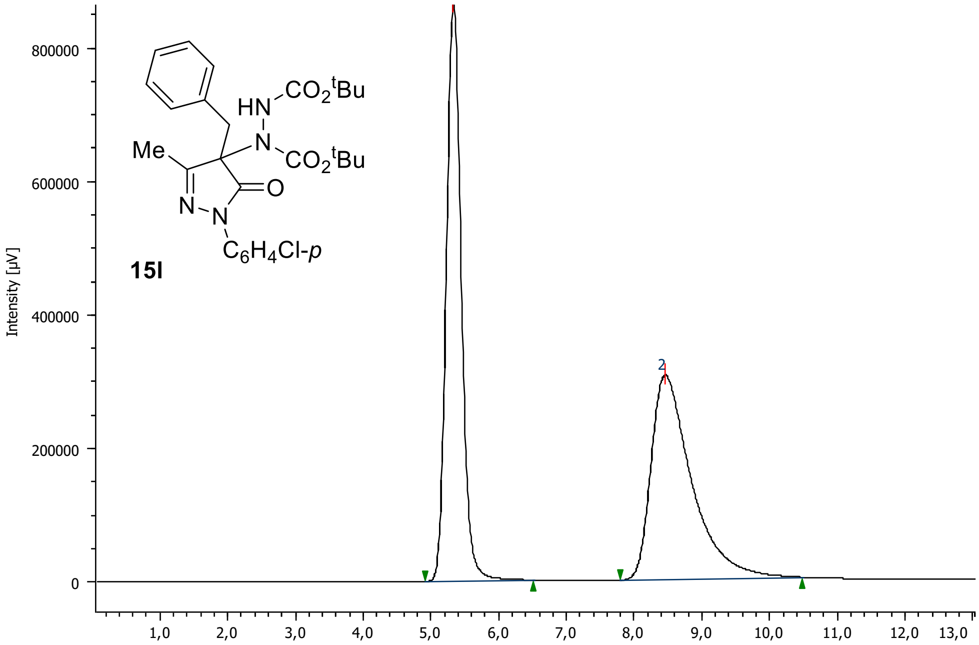

Peak Number	t_R	Area	Height	Area%	Height%	Symmetry Factor
1	**5,325**	13314463	865182	**51,124**	73,876	1,139
2	**8,450**	12728871	305950	**48,876**	26,124	1,809

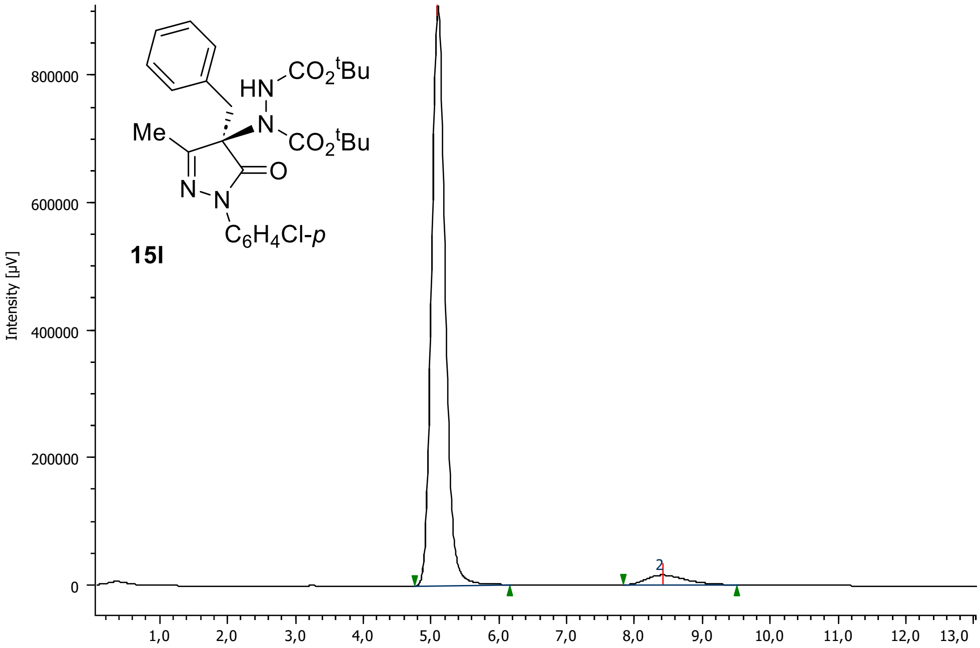

Peak Number	t_R	Area	Height	Area%	Height%	Symmetry Factor
1	**5,092**	12770080	907899	**95,138**	98,295	1,173
2	**8,400**	652616	15750	**4,862**	1,705	1,380

Di-*tert*-butyl (*R*)-1-(4-benzyl-1,3-dimethyl-5-oxo-4,5-dihydro-1*H*-pyrazol-4-yl)hydrazine-1,2-dicarboxylate (15m).

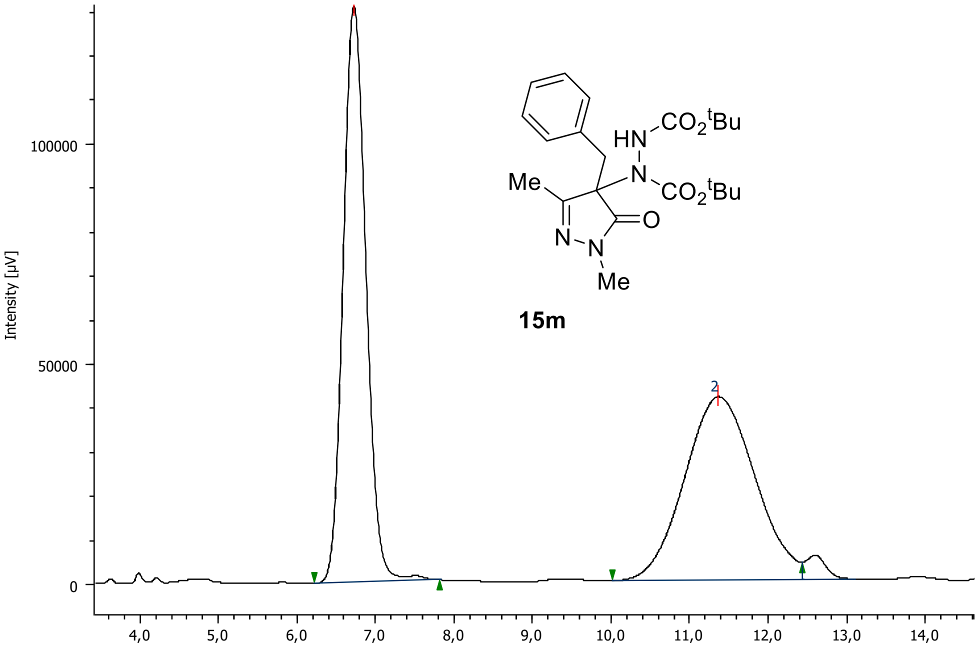

Peak Number	t_R	*Area*	*Height*	*Area%*	*Height%*	*Symmetry Factor*
1	**6,725**	2619152	130545	**50,222**	75,856	1,099
2	**11,358**	2595953	41551	**49,778**	24,144	N/A

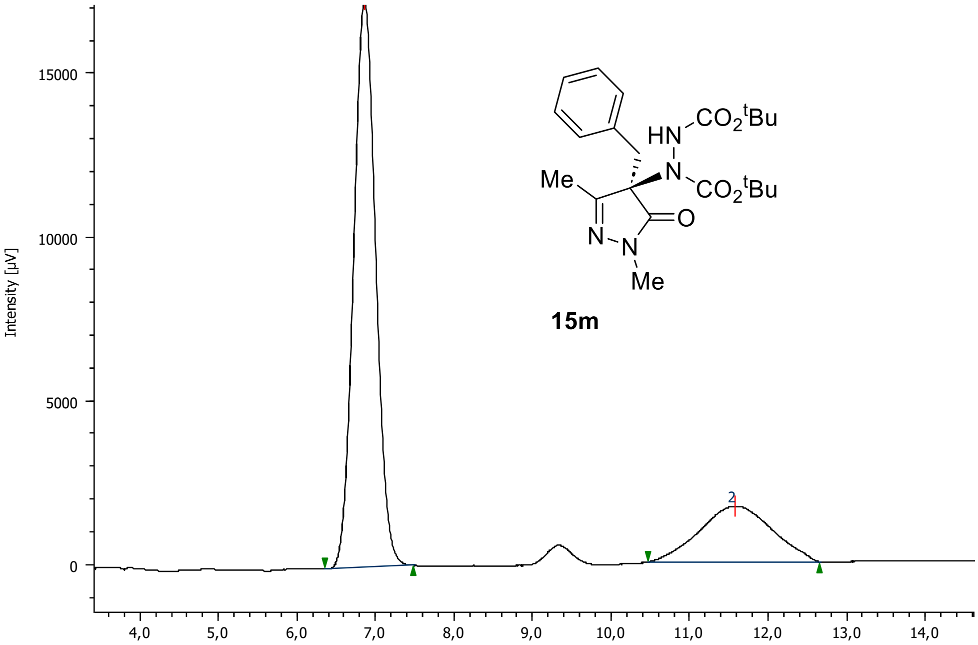

Peak Number	t_R	*Area*	*Height*	*Area%*	*Height%*	*Symmetry Factor*
1	**6,858**	335297	17276	**75,323**	91,022	1,043
2	**11,575**	109849	1704	**24,677**	8,978	0,996

Di-*tert*-butyl (*R*)-1-(4-allyl-3-methyl-5-oxo-1-phenyl-4,5-dihydro-1*H*-pyrazol-4-yl)hydrazine-1,2-dicarboxylate (15n).

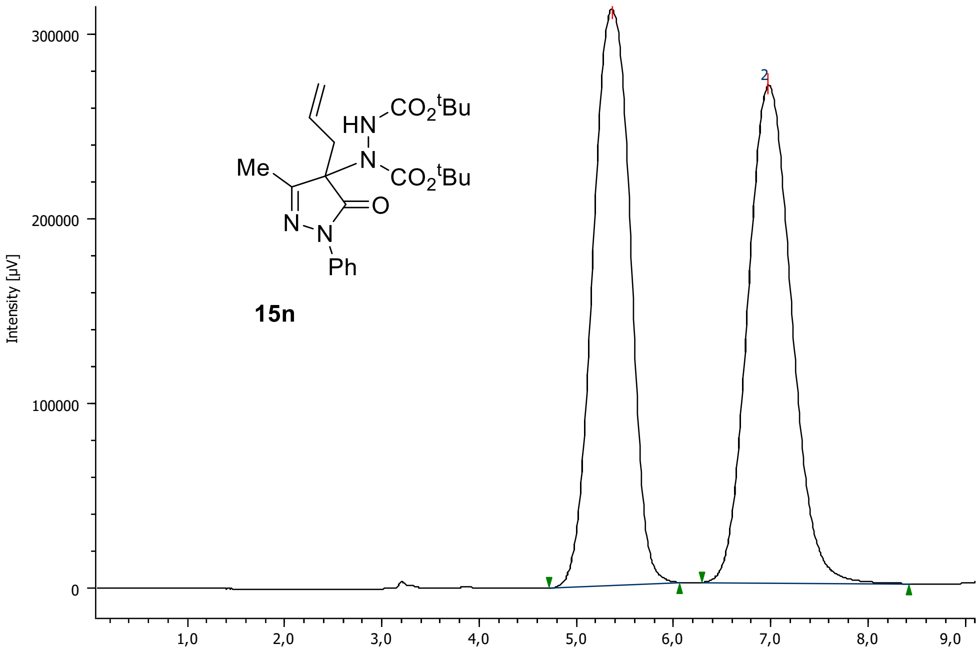

Peak Number	*t_R*	*Area*	*Height*	*Area%*	*Height%*	*Symmetry Factor*
1	**5,358**	8310603	311082	**49,815**	53,649	0,981
2	**6,967**	8372383	268762	**50,185**	46,351	1,119

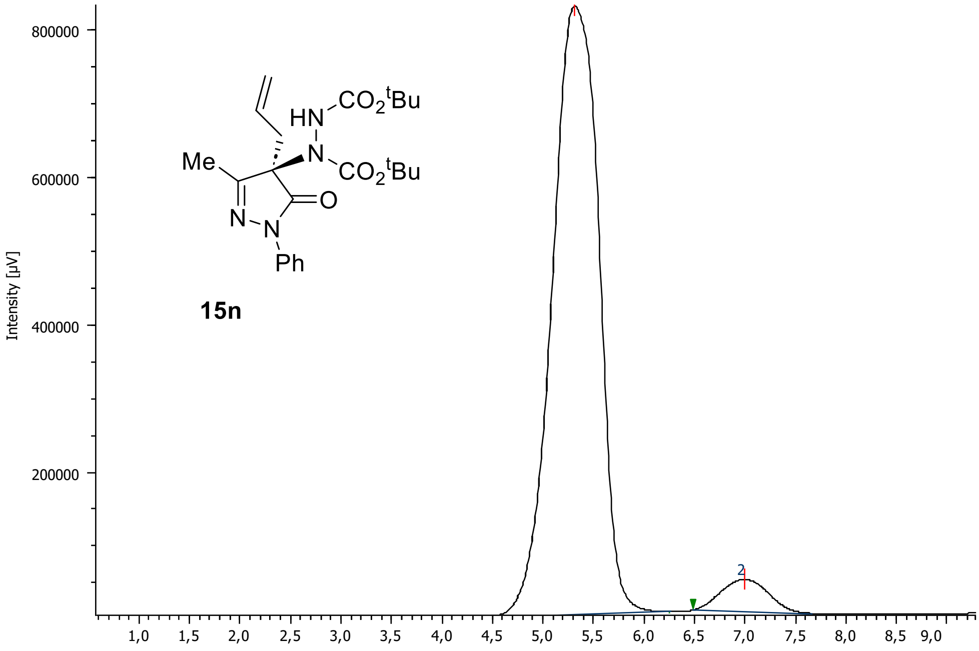

Peak Number	*t_R*	*Area*	*Height*	*Area%*	*Height%*	*Symmetry Factor*
1	**5,308**	26563430	825104	**94,963**	95,024	0,939
2	**6,983**	1409074	43204	**4,976**	4,976	1,060

Di-*tert*-butyl (*R*)-1-(4-(ethoxycarbonyl)-3-methyl-5-oxo-1-phenyl-4,5-dihydro-1*H*-pyrazol-4-yl)hydrazine-1,2-dicarboxylate (15o).

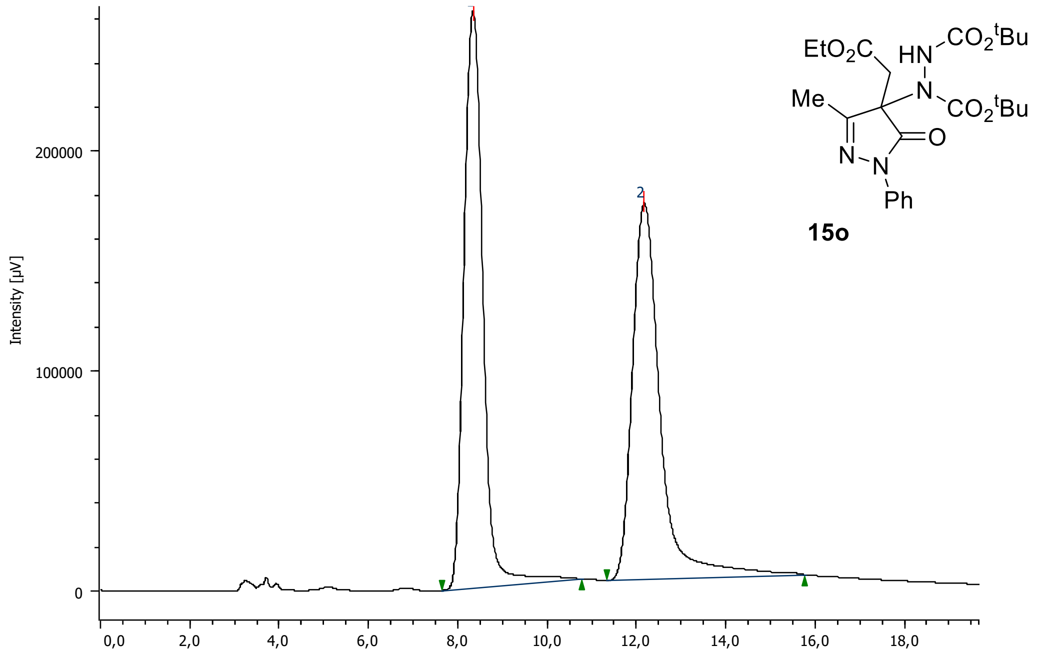

Peak Number	t_R	Area	Height	Area%	Height%	Symmetry Factor
1	**8,333**	7746957	261758	**51,064**	60,488	1,132
2	**12,158**	7424131	170983	**48,936**	39,512	1,468

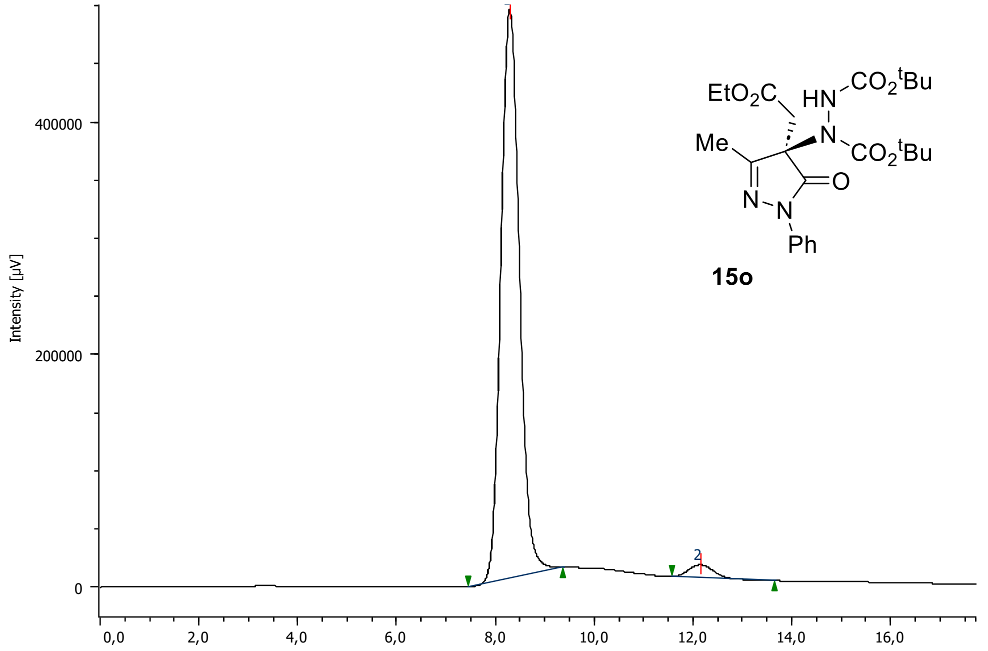

Peak Number	t_R	Area	Height	Area%	Height%	Symmetry Factor
1	**8,283**	12756947	488392	**97,021**	97,762	1,119
2	**12,133**	391748	11182	**2,979**	2,238	1,144

Di-*tert*-butyl (*R*)-1-(4-([1,1'-biphenyl]-4-ylmethyl)-3-methyl-5-oxo-1-phenyl-4,5-dihydro-1*H*-pyrazol-4-yl)hydrazine-1,2-dicarboxylate (16).

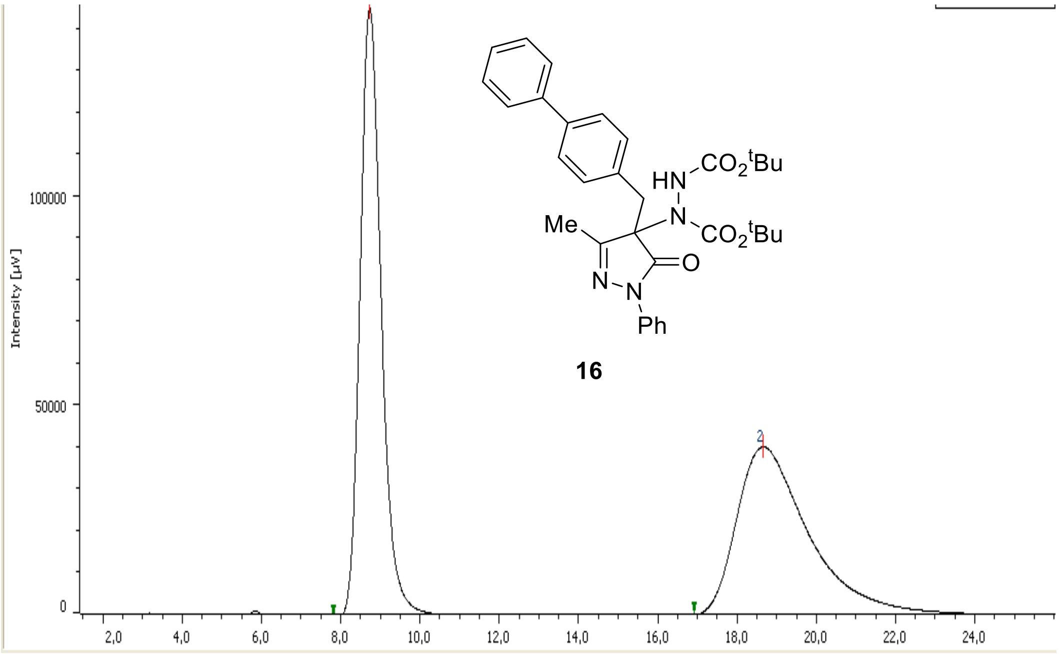

Peak Number	t_R	Area	Height	Area%	Height%	Symmetry Factor
1	**8,725**	5439623	145648	**51,164**	78,242	1,234
2	**18,650**	5192018	40503	**48,836**	21,758	1,716

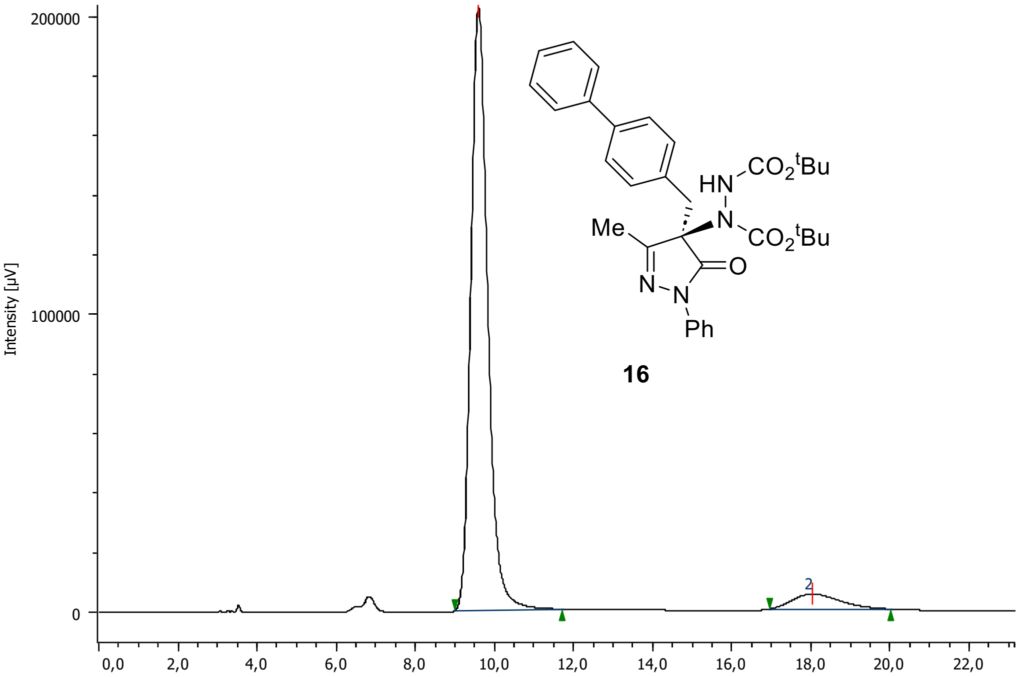

Peak Number	t_R	Area	Height	Area%	Height%	Symmetry Factor
1	**9,583**	6003559	202108	**93,423**	97,561	1,229
2	**18,017**	422651	5053	**6,577**	2,439	1,350
